# Necrotising enterocolitis biomarkers: a systematic review

**DOI:** 10.3389/fped.2025.1652566

**Published:** 2026-01-12

**Authors:** Muhammad Ashhad Faizan, Iffat Khalid, Asten Yeo, Magdalina Mazheda Fadel, Alannah Mcmahon, Philip Gavigan, Saffron O’Neill, Eman Isweisi, Gregana Semova, Edna F. Roche, Aoife Branagan, Judith Meehan, Eleanor J. Molloy

**Affiliations:** 1 Department of Paediatrics, University of Dublin, Trinity College, Dublin, Ireland; 2 Trinity College Dublin Trinity Translational Medicine Institute, Dublin, Ireland; 3 Neurodisability Children’s Health Ireland (CHI) at Temple Street, Dublin, Ireland; 4 Paediatric Endocrinology, Children’s Health Ireland (CHI) at Temple Street, Dublin, Ireland; 5 Neonatology, CHI at Crumlin, Dublin, Ireland; 6 The Coombe Hospital, Dublin, Ireland

**Keywords:** necrotising enterocolitis, biomarkers, interleukins, bell's staging, NEC (necrotizing enterocolitis), calprotectin, PEDF, DSNLT

## Abstract

**Background:**

Necrotising enterocolitis (NEC) is a severe acute inflammatory condition of the gastrointestinal tract that predominantly affects preterm neonates. The variable and often nonspecific clinical signs, followed by rapid progression into fulminant disease, and the lack of standardised definitions and biomarkers, make this condition notoriously difficult to diagnose. This systematic review aims to outline the inflammatory pathways involved in the pathogenesis of NEC and to identify potential biomarkers associated with the initial stages of disease progression.

**Methods:**

Following the PRISMA guidelines, we conducted an electronic search of the available literature using the PubMed, Embase, and Cochrane electronic databases with the following search terms (“necrotizing enterocolitis” OR “necrotising enterocolitis” OR “NEC”) AND (“biomarker*” OR “biological marker”). Studies reporting data on the diagnostic accuracy of biomarkers for NEC were included. Results were restricted to full-text articles in English, available up to November 2024. Risk of bias was assessed using the Quality Assessment of Diagnostic Accuracy Studies 2 (QUADAS-2) tool.

**Results:**

A total of 211 studies were screened, yielding 79 studies for analysis. Most studies evaluated the ability of biomarkers to differentiate Bell's stage ≥II NEC from controls or Bell's stage II from stage III. For identifying Bell's stage ≥II, faecal calprotectin (97.14% sensitivity, 100% specificity) and serum calprotectin (100% sensitivity, 96.4% specificity), as well as a panel consisting of urine proteins including Cystatin C (CST3), Pigment Epithelium Derived Factor (PEDF), and Retinol Binding Protein 4 (RET4) (96% sensitivity, 90% specificity), and maternal human milk oligosaccharide disialyllacto-N-tetraose DSNLT (90% sensitivity and specificity) demonstrated high sensitivity and specificity when sampled prior to or around the initial diagnosis of NEC. Interleukin 33 (IL-33) exhibited high accuracy.

**Systematic Review Registration:**

PROSPERO CRD42024307046.

## Introduction

Necrotising enterocolitis (NEC) is an acute gastrointestinal inflammatory condition characterised by pneumatosis intestinalis and necrosis of the intestinal mucosa usually secondary to infection. Neonates typically present with feeding intolerance, delayed gastric emptying, abdominal distention and tenderness, occult or gross blood in the stool, lethargy, apnoea, respiratory distress, and poor perfusion (1). Typically, a disease of prematurity, NEC is the most common gastrointestinal emergency in preterm infants, with approximately 7% of preterm infants with very low birth weight (VLBW) affected (2). Apart from VLBW and low gestational age, other notable risk factors for NEC include premature rupture of membranes (PROM) and invasive ventilation (3). Furthermore, NEC carries a considerable mortality, estimated to reach up to 42%, particularly in the VLBW preterm population (4).

The economic cost of NEC is high, and expenditure is estimated at 19% of total neonatal healthcare costs in the United States (5). Indeed, the associated sequelae of NEC, including short bowel syndrome (SBS), post-NEC strictures, adhesions, dysmotility, poor growth, and neurodevelopmental delay, also result in complex paediatric cases with significant implications for the healthcare system (6, 7). In addition, the incidence of NEC has remained largely unchanged in preceding decades, suggesting limited successo of current diagnostic and preventive strategies.

Currently, the gold standard for grading the severity of NEC is based on the modified Bell's criteria, which utilises clinical, pathophysiological, and radiological signs to classify the severity of NEC from stages I (suspicion of NEC) to IIIB (perforated NEC) (8). However, this diagnostic method is inherently limited, with an over-reliance on non-specific signs and symptoms such as feeding intolerance and abdominal distension, which can also be caused by other pathologies such as neonatal sepsis (9).

Due to limitations in current definitions, biomarkers for NEC and diagnostic criteria are required to complement clinical signs. Thus, the need to augment the modified Bell's staging criteria is essential to improve the management of NEC (10). Recent evidence supports the use of biomarkers as a supplement to Bell's criteria to aid clinicians in the diagnosis of NEC. As an early and accurate diagnosis is crucial for neonates with NEC due to severe complications, the need to identify a potential ideal biomarker has never been more urgent (11).

NEC biomarkers have been classified into three main categories: “non-specific”, “enhanced non-specific”, and “gut-specific” biomarkers (12). While non-specific biomarkers primarily reflect the activation of immune-mediated inflammatory pathways, enhanced non-specific biomarkers—which are derived from stool—provide a better indication of the location of tissue injury due to the nature of the sample, and gut-specific biomarkers allow for improved evaluation of gastrointestinal mucosal injury (7).

Not only is the pathogenesis of NEC a complex multifactorial phenomenon, with no precisely mechanism, but the lack of established markers coupled with non-specific biomarkers, makes a definitive diagnosis challenging (7, 50). Owing to the nature of the disease, there are a myriad of neonatal gastrointestinal pathologies that can mimic NEC or may represent a spectrum of the disease, including spontaneous intestinal perforation (SIP), intestinal malrotation with volvulus, and ileus associated with neonatal sepsis (13). There is an unmet need for rapid and accessible biomarkers to facilitate the diagnosis, which may significantly affect prognosis (12).

In this systematic review, we aimed to synthesise data on blood biomarkers for the detection of NEC. The primary objective was to compile available data on the diagnostic accuracy of both established and potential NEC biomarkers and critically appraise the quality and level of evidence of the studies, and to identify high-potential biomarkers that may aid in NEC diagnosis.

## Methods

### Search strategy and eligibility criteria

Studies were selected and systematically reviewed in accordance with the Preferred Reporting Items for Systematic Reviews and Meta-Analyses (PRISMA) statement. We conducted a review of PubMed, Embase, and Cochrane electronic databases using the search terms (“necrotizing enterocolitis” OR “necrotising enterocolitis” OR “NEC”) AND (“biomarker*” OR “biological marker”). Results were restricted to full-text articles of studies in the English language, available until November 2024. Biomarker studies were considered eligible if they reported data on the accuracy of biomarkers for identifying Bell's stage II and/or III NEC (definite and/or advanced NEC respectively). Studies that included Bell's stage I (suspected NEC) within their study populations were only included if they also reported independent accuracy data for the identification of Bell's stage II and/or III subgroups. Studies were excluded if they had a small sample size of ≤10 patients, if they used diagnostic criteria other than Modified Bell's criteria, or if they did not use or report a standardised criteria for the diagnostic confirmation of NEC due to low quality of evidence. Animal studies and articles not available for full-text retrieval were also excluded.

### Study selection

Titles and abstracts of studies identified were dually and independently assessed for eligibility by any two reviewers using Covidence. All authors were involved in the study selection. Disagreements were resolved by consensus or by discussion with other authors. Subsequently, articles were obtained for full-text screening and assessed for eligibility using the same methodology employed for the title/abstract screening.

### Data extraction and study assessment

Data extraction and quality assessment were performed by two independent reviewers (A.Y. and M.A.F). Any disagreements between reviewers were resolved by consensus or by discussion with other authors. The following data were extracted from each study: study design, type of biomarker used, criteria used for NEC diagnosis, characteristics of the study population, target group(s) and the controls used, and the timing of sampling. For each biomarker or biomarker profile identified by each study, statistical measures of biomarker accuracy (i.e., the area under the curve (AUC) value of the biomarker's receiver operating characteristics (ROC) curve and sensitivity, specificity, positive predictive value (PPV), and negative predictive value (NPV) and corresponding optimal cut-off value) were recorded (if reported). For medical NEC, the “day of diagnosis” was defined as the first day on which the diagnosis was made on clinical and/or radiological grounds (typically corresponding to Bell's stage II or higher). For surgical NEC, the term referred to the same initial diagnostic point, with surgical confirmation occurring subsequently in those requiring operative management. This definition was applied consistently across the included studies.

Quality and risk of bias assessment was performed using the Quality Assessment of Diagnostic Accuracy Studies-2 (QUADAS-2) tool (14). Studies were assessed using a standardised form containing review-specific signalling questions designed to assess risk of bias and applicability concerns across four key domains: “patient selection,” “index test,” “reference standard,” and “flow and timing.” Items were scored as “high,” “low” or “unclear”.

### Studies included and flow

**Figure 1:**
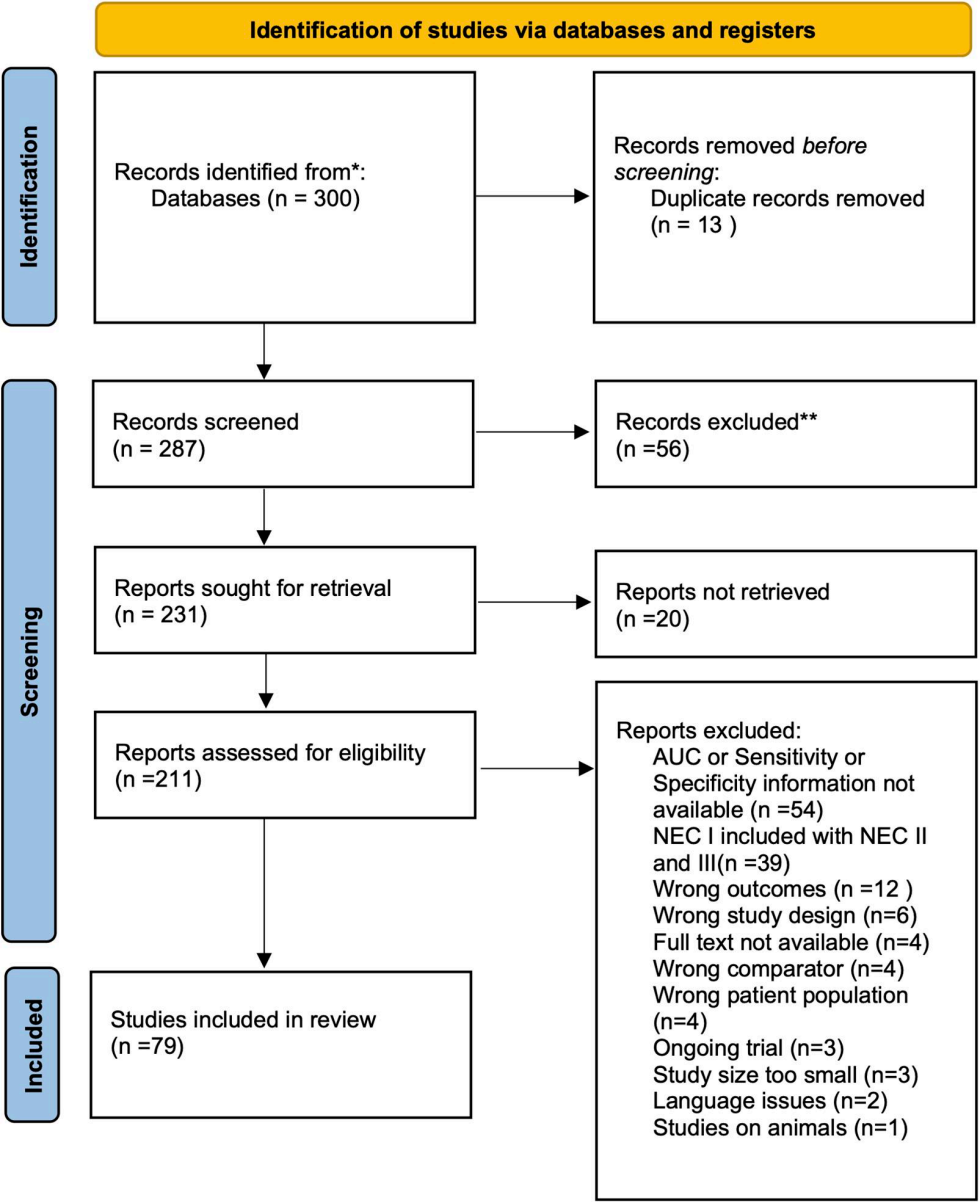
PRISMA flow diagram of study selection process. A flow diagram illustrating the study selection process, from initial database search to final inclusion. This figure shows the number of studies identified, screened, excluded, and included in the systematic review according to PRISMA guidelines.

After combining search results from PubMed (*n* = 194), Embase (*n* = 76), and Cochrane (*n* = 30) databases, we retrieved a total of 300 studies. Following the removal of 13 duplicates and excluding 56 articles through title and abstract screening, 287 articles were sought for retrieval, out of which 20 could not be retrieved. Finally, 211 articles were sought for eligibility screening. A further 132 were excluded for not meeting our eligibility criteria, resulting in a total of 79 included studies that reported accuracy data on biomarkers or biomarker profiles. The phases of the review, along with the number of records identified, included, and excluded (with reasons provided) at each stage, are presented using the PRISMA flow diagram in Figure 1. The main reasons for exclusion were the lack of reported accuracy data (e.g., sensitivity, specificity, AUC values) and the adoption of a study design that included Bell's stage I within their study populations but did not report accuracy data for the identification of their Bell's stage II and/or III subgroups. Any studies which used diagnostic criteria other than the Modified Bell's criteria were excluded in an effort to draw a fair comparison.

### General characteristics of biomarker studies

This review captured a global perspective of the disease prevalence (Table 1). Moreover, there was a good mix of study designs such as pilot and case control studies, prospective and retrospective cohort studies. Biomarkers assessed across these studies varied widely between non-invasive markers, inflammatory markers, blood markers, and even machine learning models incorporating various parameters.

**Table 1:** General characteristics of included studies evaluating biomarker performance.

Biomarker	Sample	Reference	Study design	Study population	Characteristics of controls	Target condition vs. control	Timing of sampling	Cut-off value (units)	AUC (95% CI)	Sensitivity (%)	Specificity (%)
Neutrophil CD64+	Blood	Lam et al. (15)	Prospective Cohort	*n* = 218 VLBW neonates with 33 proven sepsis/NEC cases, 22 clinical sepsis/NEC cases, 100 non-sepsis/non-NEC, and 63 asymptomatically activated controls	*n* = 163 Preterm Neonates without NEC/Sepsis	NEC II/III vs. Controls	Prior to diagnosis	5,655 antibody-PE molecules bound/cell (PE units)	0.95	89	98
TRAIL	Blood	Dong et al. (14)	Prospective Case Contol	*n* = 88, Preterm Neonates with 30 NEC cases (including NEC II (18) and NEC III (12), 29 Sepsis, and 29 controls, BW < 2,500 g, GA < 37weeks	*n* = 29 Premterm Healthy neonates without NEC or other infectious disease	NEC II/III vs. Controls	At onset of NEC symptoms	5.279	0.793 (0.666–0.920)	96.6	66.7
TSLP	Blood	Dong et al. (14)	Prospective Case Contol	*n* = 88, Preterm Neonates with 30 NEC cases (including NEC II (18) and NEC III (12), 29 Sepsis, and 29 controls, BW < 2,500 g, GA < 37weeks	*n* = 29 Premterm Healthy neonates without NEC or other infectious disease	NEC II/III vs. Controls	At onset of NEC symptoms	−0.177	0.814 (0.702–0.926)	96.6	63.3
TNFSF14	Blood	Dong et al. (14)	Prospective Case Contol	*n* = 88, Preterm Neonates with 30 NEC cases (including NEC II (18) and NEC III (12), 29 Sepsis, and 29 controls, BW < 2,500 g, GA < 37weeks	*n* = 29 Premterm Healthy neonates without NEC or other infectious disease	NEC II/III vs. Controls	At onset of NEC symptoms	5.024	0.675 (0.531–0.818)	72.4	70
IL-6	Blood	Wang et al. (16)	Retrospective Cohort	*n* = 150 Preterm and Term infants with 58 NEC II and 92 NEC III cases	*n* = 58 Preterm infants with NEC II	NEC II vs. NECIII	At the time of diagnosis	–	0.635	60.3	68.35
IL-8	Blood	Dong et al. (14)	Prospective Case Contol	*n* = 88, Preterm Neonates with 30 NEC cases (including NEC II (18) and NEC III (12), 29 Sepsis, and 29 controls, BW < 2,500 g, GA < 37weeks	*n* = 30 Pretem Neonates with NEC II (18) or NEC III (10)	NEC II vs. NEC III	At onset of NEC symptoms	9.334	0.778 (0.601–0.955)	61.5	88.2
IL-33	Blood	Cakir et al. (53)	Prospective Case Control	*n* = 84 Preterm infants, BW < 1,500 g, GA < 32weeks	*n* = 42, Preterm infants matched for GA and weight	NEC Stage > II vs. Controls	On day of diagnosis (d = 1)	>3.1 ng/mL	0.838 (0.658–1.000)	100	55.6
DSNLT	Maternal Breast Milk	Masi et al. (17)	Prospective Cohort	*n* = 77, Preterm infants, GA < 32weeks	*n* = 37, Preterm healthy ifnants matched by BW and GA	NEC > II vs. Controls	As close as possible to disease onset	241 nmol/mL	0.947 (0.88–0.981)	90	90
CRP	Blood	Guo et al. (18)	Retrospective	*n* = 90, Preterm Infants with 52 NEC II cases and 38 NEC III cases, GA < 37weeks	*n* = 52 Preterm infants diagnosied with NEC II	NEC II vs. NEC III	1 day after diagnosis (d = 1)	14.65 mg/l	0.67 (0.55–0.78)	79	67
Gal-4	Blood	Fundora et al. (19)	Prospective Cohort	*n* = 167, Preterm of Full-term infants	*n* = 28, matched controls	NEC Stage III vs. Controls	Within 24–48 h post diagnosis	>0.7 ng/mL	0.84	71	89
L-FABP	Blood	Benkoe Mechtler et al. (20)	Prospective	*n* = 29, BW < 2,000 g	*n* = 14, GA, BW, and age at diagnosis matached infants	NEC Stage > II vs. Controls	At onset of symptoms	–	0.95 (0.87–1.00)	–	–
I-FABP	Blood	Liu et al. (21)	Prospective Cohort	*n* = 70 Preterm Neonates with 18 NEC II cases, 12 NEC III, and 40 Controls,BW < 1,500 g,GA < 32weeks,	*n* = 40, Preterm Healthy infants without NEC	NEC II/III vs. Controls	Prior to diagnosis	2.54 ng/mL	0.897 (0.825–0.968)	76.7	87.5
I-FABPu	Urine	Coufal et al. (22)	Retrospective	*n* = 37, Preterm and Term infants admitted to Paediatric Surgery Department	*n* = 9 Infants with sepsis	NEC Stage > II vs. Sepsis	At time of enrolment or post surgery	–	0.831	–	–
IAP	Stool	Heath et al. (23)	Multicentre Prospective	*n* = 136 Preterm infants	*n* = 111, preterm infants with suspected NEC I/II (19) and Non-Nec (92)	NEC III vs. Controls	Biweekly	–	0.97 (0.93–1.00)	–	–
Lipocalin-2	Stool	Thiubalt et al. (24)	Multicentre Prospective	*n* = 134 VLBW infants with 8 NEC and Non-NEC cases	*n* = 126 preterm neonates with mean GA < 32 weeks and BW < 1,500 g	NEC III vs. Controls	Sample collected 10 days prior to NEC diagnosis	227 µg/g feces	0.82	70	77
Endocan	Blood	Cakir et al. (53)	Prospective Case Control	*n* = 84, Preterm infants, GA < 32 weeks, BW < 1,500 g	*n* = 42 infants matached for GA and BW	NEC Stage > II vs. Controls	On day of diagnosis (d = 1)	>1,414.65 ng/mL	1.000 (1.000–1.000)	100	83.3
Urinary Caveolin-1	Urine	Corebima et al. (25)	Single Centre Cohort	*n* = 34 Preterm and Term Neonates with 12 NEC II/III cases, 12 without NEC, and 10 Healthy controls	*n* = 24 Preterm and Term Neonates without NEC or anyother disease	NEC II/III vs. Controls	3rd day post diagnosis (d = 3)	≥17.81 ng/dL	–	87.5	75
Panel (IL-8, IL-24, CCL20)	Blood	Dong et al. (14)	Prospective Case Contol	*n* = 88, Preterm Neonates with 30 NEC cases (including NEC II (18) and NEC III (12), 29 Sepsis, and 29 controls, BW < 2,500 g, GA < 37weeks	*n* = 29 Premterm Healthy neonates without NEC or other infectious disease	NEC II/III vs. Controls	At onset of NEC symptoms	–	0.909 (0.825–1.00)	100	85
Fibrinogen	Blood	Feng et al. (13)	Retrospective	*n* = 114 Preterm and Term Neonates with 68 NEC II/ Medical Treatmet and 46 NEC III/Surgical intervention cases	*n* = 68 suspected NEC II or NEC III receving medical treatment only	NEC II vs. NEC III	Post diagnosis (12 h after)	1.09 (g/L)	0.715	50	85.29
Coagulant Factor XIII	Blood	Guo-Zhong et al. (26)	Case Control	*n* = 84 Preterm and Term Neonates with 43 NEC II, 41 NEC III cases, and 24 controls	*n* = 24 Preterm neonates without NEC, matached for GA and BW	NEC II/III vs. Controls	Prior to diagnosis	–	0.91	–	–
MCP-4	Blood	Dong et al. (14)	Prospective Case Contol	*n* = 88, Preterm Neonates with 30 NEC cases (including NEC II (18) and NEC III (12), 29 Sepsis, and 29 controls, BW < 2,500 g, GA < 37weeks	*n* = 29 Premterm Healthy neonates without NEC or other infectious disease	NEC II/III vs. Controls	At onset of NEC symptoms	15.661	0.737 (0.608–0.865)	51.7	90
CCL20	Blood	Dong et al. (14)	Prospective Case Contol	*n* = 88, Preterm Neonates with 30 NEC cases (including NEC II (18) and NEC III (12), 29 Sepsis, and 29 controls, BW < 2,500 g, GA < 37weeks	*n* = 30 Pretem Neonates with NEC II (18) or NEC III (10)	NEC II/III vs. Controls	At onset of NEC symptoms	9.994	0.851 (0.745–0.956)	96.6	66.7
OPG	Blood	Dong et al. (14)	Prospective Case Contol	*n* = 88, Preterm Neonates with 30 NEC cases (including NEC II (18) and NEC III (12), 29 Sepsis, and 29 controls, BW < 2,500 g, GA < 37weeks	*n* = 30 Pretem Neonates with NEC II (18) or NEC III (10)	NEC II vs. NEC III	At onset of NEC symptoms	9.365	0.851 (0.712–0.990)	61.5	94.1
IL-6	Blood	Wang et al. (16)	Retrospective Cohort	*n* = 150 Preterm and Term infants with 58 NEC II and 92 NEC III cases	*n* = 58 Preterm infants with NEC II	NEC II vs. NECIII	At the time of diagnosis	-	0.635	60.3	68.35
IL-8	Blood	Dong et al. (14)	Prospective Case Contol	*n* = 88, Preterm Neonates with 30 NEC cases (including NEC II (18) and NEC III (12), 29 Sepsis, and 29 controls, BW < 2,500 g, GA < 37weeks	*n* = 30 Pretem Neonates with NEC II (18) or NEC III (10)	NEC II vs. NEC III	At onset of NEC symptoms	9.334	0.778 (0.601–0.955)	61.5	88.2
CRP	Blood	Guo et al. (18)	Retrospective	*n* = 90, Preterm Infants with 52 NEC II cases and 38 NEC III cases, GA < 37weeks	*n* = 52 Preterm infants diagnosied with NEC II	NEC II vs. NEC III	1 day after diagnosis (d = 1)	14.65 mg/l	0.67 (0.55–0.78)	79	67
L-FABP	Blood	Benkoe Mechtler et al. (20)	Prospective	*n* = 29, BW < 2,000 g	*n* = 14, GA, BW, and age at diagnosis matached infants	NEC Stage > II vs. Controls	At onset of symptoms	-	0.95 (0.87–1.00)	-	-
I-FABP	Blood	Liu et al. (21)	Prospective Cohort	*n* = 70 Preterm Neonates with 18 NEC II cases, 12 NEC III, and 40 Controls,BW < 1,500 g, GA < 32weeks,	*n* = 40, Preterm Healthy infants without NEC	NEC II/III vs. Controls	Prior to diagnosis	2.54 ng/mL	0.897 (0.825–0.968)	76.7	87.5
A2ML1	Blood	Sylvester et al. (27)	Multicentre Prospective	*n* = 119 Preterm Neoantes with 85 NEC cases [inlclduding NEC II (59) NEC III (26)], 17 Sepsis cases, and 17 controls	*n* = 17 Preterm Healthy controls with matched GA and BW	NEC II/III vs. Controls	At time of diagnosis	-	0.849	80	76
PEDF	Blood	Sylvester et al. (27)	Multicentre Prospective	*n* = 119 Preterm Neoantes with 85 NEC cases [inlclduding NEC II (59) NEC III (26)], 17 Sepsis cases, and 17 controls	*n* = 17 Preterm Healthy controls with matched GA and BW	NEC II/III vs. Controls	At time of diagnosis	–	0.834	80	69
Sodium	Blood	Zhang et al. (28)	Retrospective Cohort	*n* = 249 Preterm Neonates with 22 NEC I, 91 NEC II, and 136 NEC III cases, BW < 2,000 g, GA < 36 weeks	*n* = 113 Preterm Neonates with NEC I/II	NEC II vs. NEC III	At day of diagnosis	≤135 mmol/L	0.875	0.84	0.8

QUADAS-2 was applied to the studies after screening to highlight the risk of bias (Supplementary Table S2.0). Overall, the risk of bias was high in most studies, particularly in the “patient selection” domain, due to a lack of consecutive sampling, the use of retrospective and case–control designs, and the use of controls that can result in an overestimation of accuracy. Other factors contributing to the high risk of bias include a lack of information on blinding methods used for both the index test and the reference standard (diagnosis of NEC based on Bell's criteria). Concerns about the applicability of the studies also arose from the inclusion of neonates with stage II/III NEC at the time of enrolment, and from the inclusion of diseases different from stage II/III NEC as target conditions—such as sepsis or Bell stage I NEC.

### Biomarker accuracy

Based on the preliminary categories proposed by Ng et al. (12), biomarkers were classified into six groups: (1) haematological indices, (2) cytokines and chemokines, (3) acute phase reactants, (4) enhanced non-specific markers, (5) other non-specific markers, and (6) gut-specific markers. Biomarker panels containing markers from multiple groups were included in both categories for comparison alongside studies investigating the use of scores and artificial intelligence-based machine models. Area Under Curve (AUC) represents the ability of a biomarker to distinguish between two groups. Being derived from the Receiver Operating Curve (ROC), a value <0.5 indicates poor discriminative power, while >0.7 represents moderate discrimination. AUC = 0.8 reflects a good discriminatory power, while a value of >0.9 implies excellent discrimination.

Sensitivity and specificity values were classified as high (>85%), moderate (70%–85%), and low (<70%), with overall biomarker accuracy deemed high if both sensitivity and specificity exceeded 85%.

The analysis focused on the ability of biomarkers or biomarker panels to distinguish or diagnose NEC across two broad domains: (1) distinguishing between both stages of NEC and controls (i.e., non-NEC cases), and (2) discriminating between Medical/NEC Stage II and Surgical/NEC Stage III. Following this categorisation, biomarkers were grouped based on the timing of sample collection into three categories: (1) performance prior to the day of diagnosis, (2) performance on the day of diagnosis, and (3) performance after the diagnostic day.

## Results

After screening and evaluation, 79 studies were included in the review (Table 1) and outlined in PRISMA flow diagram (Figure 1).

### Accuracy of NEC biomarkers by target condition vs. control/comparator

Most of the studies investigated the diagnostic ability of biomarkers to distinguish between NEC stage II and Controls, followed by studies investigating the accuracy of methods of discriminating between NEC stage II (medical NEC) and NEC stage III (surgical NEC). Furthermore, our results also generated studies considering either NEC II or NEC III individually in the context of infants with sepsis (Supplementary Table S1.1).

### Medical and surgical NEC vs. controls

**Figure 2:**
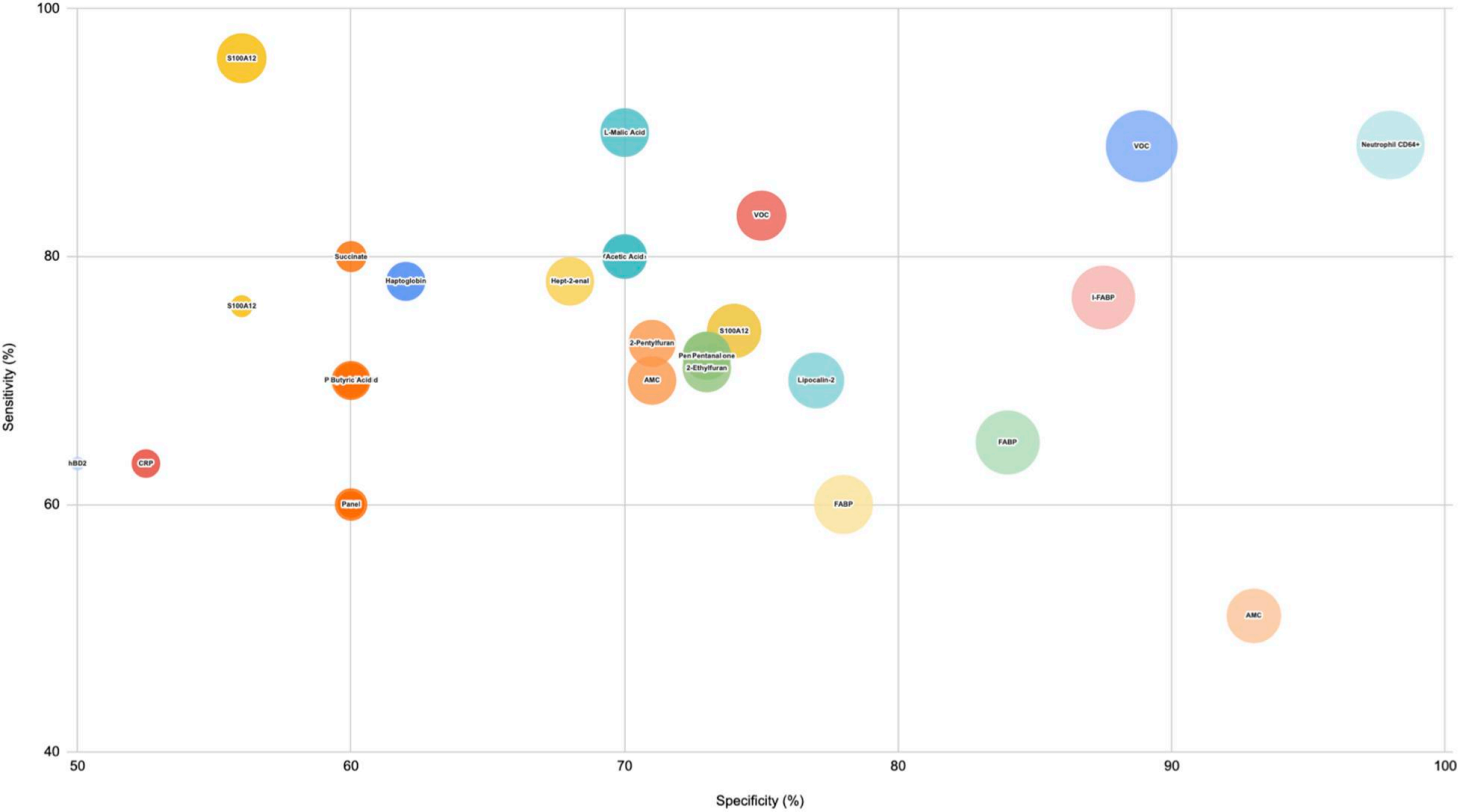
Biomarker performance for medical and surgical NEC vs. Controls Prior to Diagnosis. This bubble plot illustrates the performance of biomarkers from various categories in distinguishing medical and surgical NEC cases from controls before the day of diagnosis. Bubble sizes represent relative diagnostic effectiveness, with larger bubbles indicating stronger biomarker performance across sensitivity, specificity, and AUC values.

*Prior to the day of diagnosis*: The results of the biomarker performance from different categories have been represented in a bubble plot (Figure 2). Biomarkers with the highest sensitivity and specificity recorded to be Volatile Organic Compounds (0.99 AUC, 88.9% sensitivity and specificity for rising one day prior to diagnosis) and Neutrophils CD64+ (AUC 0.93, 89% sensitivity, 98% specificity). Biomarkers offering moderate sensitivity and specificity included a fall in L-malic Acid (AUC 0.76), I-FABP (76% sensitivity, 87.5% specificity). In contrast, biomarkers like increasing S100A12 and haptoglobin demonstrated high sensitivity (60%–80%) but lower specificity (50%–70%), while an increase in absolute monocyte count and S100A8/A9 fell short of sensitivity but had a high specificity value.

Under the acute phase reactant category, Calgranulin-C (S100A12) is reported to have AUC 0.77 with 96% sensitivity and 56% specificity to discriminate between Medical NEC and the controls, followed by AUC of 0.81 with 74% sensitivity and specificity to discriminate between Surgical NEC and the controls.

For the Haematological indices category, absolute monocyte count has been recorded to have an acceptable predictive value in terms of identifying both stages of NEC from controls (AUC 0.81). Moreover, neutrophil CD64 + level is reported to have excellent sensitivity and specificity (89% and 98%) towards predicting NEC. For the enhanced non-specific category, most of the studies included have reported values with regard to volatile organic compounds such as Pent-1-ene-3-one, Hept-2-enal, and 2-Ethyl-furan, and with AUC value of 0.76, respectively. However, Hept-2-enal was reported to have the highest sensitivity (78%) but the lowest specificity (68%). In contrast, TCA metabolite L-malic acid is reported to outperform the other markers in the category (AUC 0.76, with 90% sensitivity and 70% specificity).

**Figure 3:**
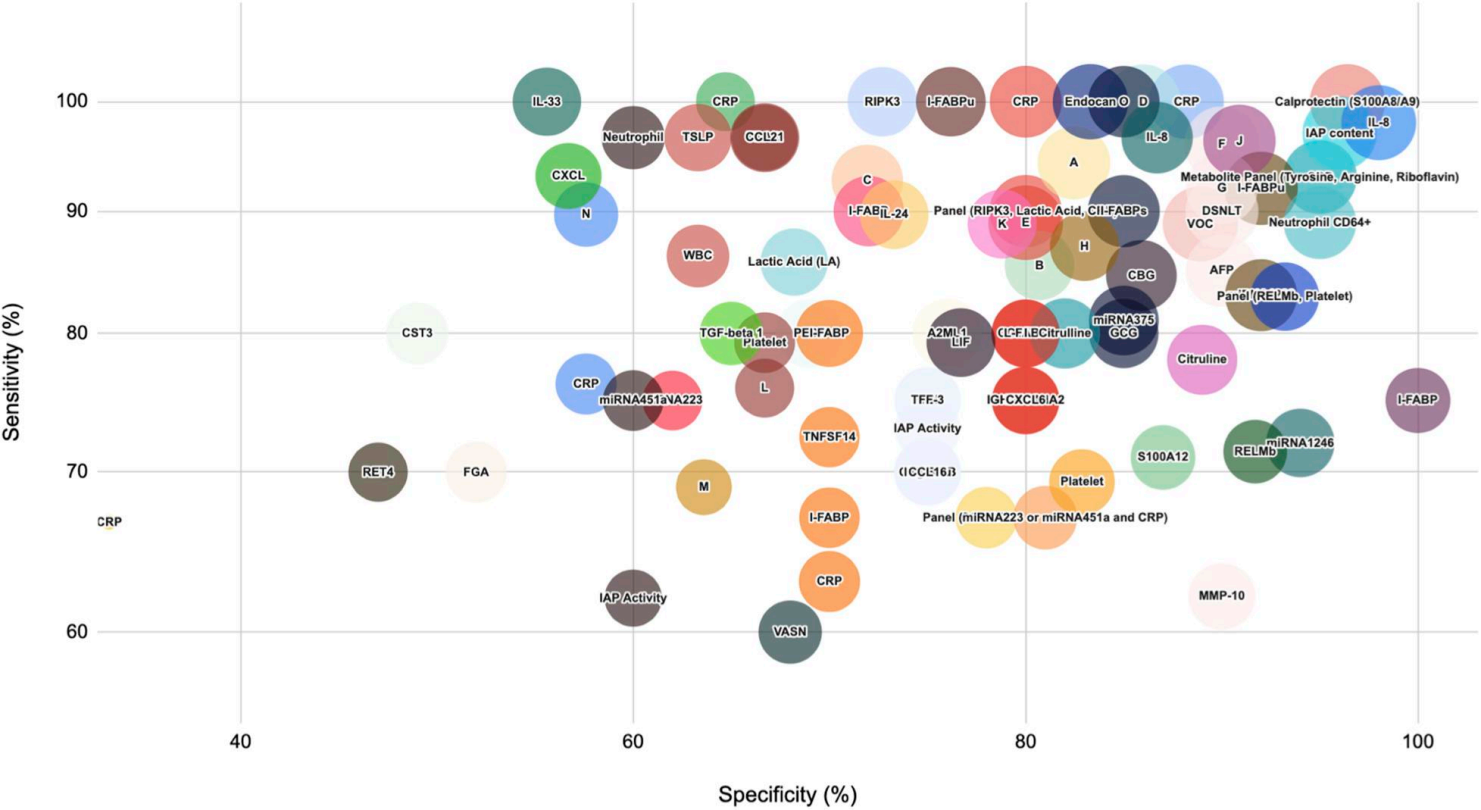
Biomarker performance for medical and surgical NEC vs. Controls At the day of Diagnosis. This bubble plot illustrates the performance of biomarkers from various categories in distinguishing medical and surgical NEC cases from controls at the day of diagnosis. Bubble sizes represent relative diagnostic effectiveness, with larger bubbles indicating stronger biomarker performance across sensitivity, specificity, and AUC values. **(A)** Machine learning model with training dataset (all features included). **(B)** Machine learning model with training dataset (abdominal radiographs only) **(C)** Machine learning model with training dataset (clinical parameters only). **(D)** Machine learning model with validation dataset (all features included) **(E)** Panel of biomarkers from one study: CST3, PEDF, and RET4. **(F)** Panel of biomarkers from a second study: CST3, PEDF, and RET4 **(G)** Prediction Model involving Microbiome Data set 1. **(H)** Prediction Model involving Microbiome Data set 2. **(I)** Panel of biomarkers: L-FABP, I-FABP, TFF-3 (LIT Score). J: Doppler Flowmetry of Superior Mesenteric Artery: Resistivity Index (RI) **(K)** Doppler flowmetry of Superior Mesenteric Artery: Pulsatility Index (PI) **(L)** Doppler flowmetry of Portal Vein: Systolic Velocity (SV). (M: Doppler flowmetry of Portal Vein: Mean Flow Velocity (MFV) N: Doppler flowmetry of Portal Vein: Volumetric Blood Flow (VBF) O: Panel of biomarkers: IL-8, IL-24, CCL20.

For the gut-specific biomarker category, increased I-FABP reported to have the highest AUC of 0.9 with excellent sensitivity and specificity values (76.7%, 87.5%), while a decrease in Short Chain Fatty Acid biomarker, Acetic Acid, reports promising predictive diagnostic value (AUC 0.73, Sensitivity 80%, Specificity 70%). A rise in Lipocalin-2, from another non-specific category, is reported to have AUC 0.82 with 70% sensitivity and 77% specificity. *On the day of diagnosis* (Figure 3): Biomarkers such as Calprotectin (S100A8/A9), RIPK3, Endocan, and Neutrophil CD64+, disialyllacto-N-tetraose (DSNLT), and a metabolomic panel comprising Tyrosine, Arginine, Riboflavin demonstrated very high sensitivity and specificity values, followed by IL-33, CRP, and CXCL1 with high sensitivity (90%–100%) but low specificity (60%–70%). All of the above are likely to be elevated with the exception of DSNLT, a human milk oligosaccharide. In contrast, a miRNA panel comprising miRNA223 or miRNA451a with CRP offered higher specificity (81%) with a moderate sensitivity (67%).

In terms of acute phase reactants, Calprotectin and CRP both have reported a very high AUC value (0.99, 0.98) with 100% sensitivity. Endocan from the nonspecific biomarker category is reported to have an AUC of 1.00 with 100% sensitivity. Panels of biomarkers such as proteins A2ML1, CD14 CST3, PDF4, RET4, and VASN have an AUC 0.997 with 96% sensitivity and 90% specificity—all of the above being raised with the exception of RET4, its synthesis being suppressed by inflammation.

Another metabolite panel consisting of Tyrosine, Arginine and Riboflavin levels has high diagnostic accuracy (AUC0.963). MiRNA 1290 is also reported to have excellent diagnostic potential (AUC 0.92 with 83% sensitivity and 92% specificity). For the gut-specific biomarkers category, DSNLT reveals a high diagnostic ability with 0.947 AUC and 90% sensitivity and specificity. Another non-invasive, gut-specific marker considered—measuring the resistivity index (RI) across the superior mesenteric artery: this reports a very high AUC of 0.93 with exceptional sensitivity (96.3%) and specificity (90%).

In terms of novel biomarkers, various studies have reported optimistic values for biomarkers such as IAP (AUC 0.97), Gal-4 and Gal-5 (AUC 0.84), and RELM-b (AUC 0.739). Most of the studies reported mixed values for I-FABP, with the urinary I-FABP reporting stronger values (0.864 with 100% sensitivity) compared to Liver/L-FABP (AUC 0.84).

Within the cytokines and chemokines category, a wide variety of molecules have been reported to demonstrate exceptional sensitivity such as TRAIL, TSLP, CCL21, IL-8 (each with 96.6% sensitivity) while some biomarkers performed well as a combination panel such as Interlukin-8 (IL-8), Interlukin-24 (IL-24) and Chemokine Ligand 20 (CCL-20) (AUC 0.909, 100% sensitivity and 85% specificity). Many novel biomarkers, such as Tissue Growth Factor beta one (TGF-beta 1) (0.738, 80% sensitivity and 65% specificity), IL-33 (0.838, 100% sensitivity and 55.6% specificity), and tumor necrosis factor superfamily member 14 (TNFSF14) (AUC 0.675, 72% sensitivity and 70% specificity) have also shown promising results. Between the interleukin family of cytokines, IL-8 (0.907) outperformed IL-6 (0.729). In the Hematological indices category, Neutrophils have been reported to have an exceptional diagnostic profile (0.93, 89% sensitivity and 95% specificity).

**Figure 4:**
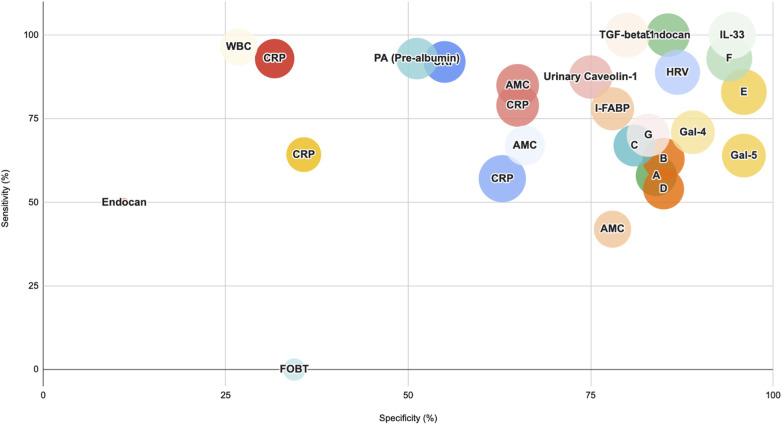
Biomarker performance for medical (stage II) and surgical (stage III) NEC vs. Controls Post-Diagnosis. This bubble plot illustrates the performance of biomarkers from various categories in distinguishing medical and surgical NEC cases from controls after diagnosis. Bubble sizes represent relative diagnostic effectiveness, with larger bubbles indicating stronger biomarker performance across sensitivity, specificity, and AUC values. A: Panel of biomarkers: CRP, miRNA223, B: Panel of biomarkers: CRP, miRNA451a, C: Combined Values for Panel A and Panel B, D:Panel of biomarkers: CRP, S100A8/A9 (Calprotectin), E:Panel of biomarkers: CRP, miRNA, F:Panel of biomarkers: SAA, I-FABP, G:N/L Ratio (Neutrophil/Lymphocyte).

*Post Diagnostic period* (Figure 4): Biomarkers such as Prealbumin, Urinary Caveolin- 1 (CAV1), Intestinal Fatty Acid Binding Protein (I-FABP) and Absolute Monocyte Count (AMC) offered a reasonable consensus between sensitivity and specificity while molecules from the Galectin family Gal-4, Gal-5, Tissue Growth Factor beta 1 (TGF-beta 1), and Interleukin-33 (IL-33) recorded moderate to high sensitivity and specificity. In contrast, Endocan did not present a promising profile for the post-diagnostic period.

In the acute phase reactant category (such as CRP, miRNA), most of the studies have reported biomarkers with moderate prognostic value. For instance, one study reports AUC 0.789 with 79% sensitivity and 65% specificity one day after diagnosis, while another study reports AUC of 0.486 with 64.3% sensitivity and 35.7% specificity. However, combining CRP with a miRNA and using it as a panel improves the prognostic value (83% sensitivity and 96% specificity). In contrast to this, biomarkers such as prealbumin levels have been reported with better prognostic profile (AUC 0.743 with 93% sensitivity and 51.2% specificity) one day after diagnosis, while novel molecules like Endocan (100% sensitivity, 85% specificity) and Urinary Caveolin-1 (87.5% sensitivity and 75% specificity) appear to perform better on 3rd day post diagnosis. This trend indicates that some biomarkers may be more effective at disease progression over time.

For gut-specific biomarkers, a panel consisting of I-FABP and SAA reports AUC of 0.941, while Gal-5 outperformed Gal-4 with better specificity (96% vs. 89%) despite having the same AUC values (0.84).

### Medical NEC vs. surgical NEC

The biomarker performance to discriminate between NEC Stage III/ surgical NEC and NEC Stage II/ Medical NEC. Each biomarker is assessed by its molecular “biomarker category”, sample timing, confidence interval, sensitivity, and specificity.

**Figure 5:**
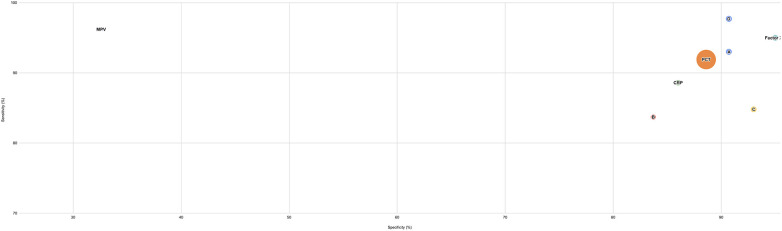
Biomarker performance to differentiate medical NEC (stage II) from surgical NEC (stage III) prior to diagnosis. This figure presents the performance of biomarkers in distinguishing NEC Stage III (surgical NEC) from NEC Stage II (medical NEC) across molecular biomarker categories. Each biomarker is evaluated based on sample timing, sensitivity, specificity, and confidence intervals, highlighting effectiveness in discrimination prior to the day of diagnosis. A: Panel of biomarkers: rintSO2, PCT B: Panel of biomarkers: rintSO2, MPV C: Panel of biomarkers: PCT, MPV. D: Panel of biomarkers: rintSO2, PCT, MPV.

*Prior to the day of diagnosis* (Figure 5): A panel consisting of rintSO2, PCT, and MPV demonstrated the highest sensitivity and specificity, alongside other biomarkers like PCT and Coagulant Factor XIII. In contrast, a panel involving just the rintSO2 and PCT reflects moderate sensitivity and specificity, while MPV by itself promises high sensitivity but lacks strong specificity.

In the acute phase reactant category, PCT has excellent discriminatory power between the two stages (PCT: 0.919, 88.6%, 86%) while combining it with other factors such as MPV and rintSO2 improves the diagnostic ability further (AUC 0.986, 97.7% sensitivity and 90.7% specificity). In the haematological indices category, MPV by itself also reports a promising ability to discriminate between the stages with 96.2% sensitivity, but poor specificity, 32.6%. Coagulant Factor XIII was an effective pre-diagnostic biomarker with an AUC of 0.958.

**Figure 6:**
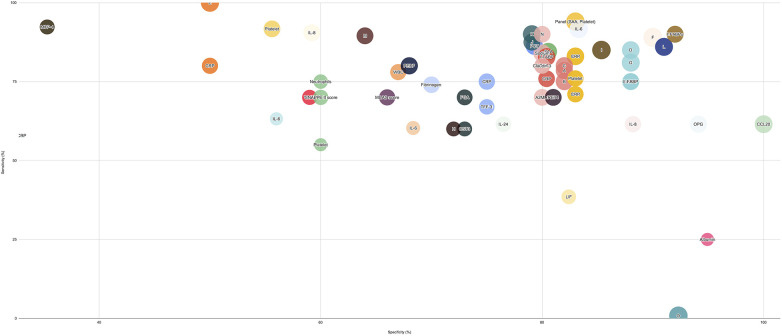
Biomarker performance to differentiate medical NEC (stage II) from surgical NEC (stage III) at the day of diagnosis. This figure presents the performance of biomarkers in distinguishing NEC Stage III (surgical NEC) from NEC Stage II (medical NEC) across molecular biomarker categories. Each biomarker is evaluated based on sample timing, sensitivity, specificity, and confidence intervals, highlighting effectiveness in discrimination prior at the day of diagnosis. **(A)** Panel (Nomogram): Hypothermia, absent bowel sounds, WBC > 20 × 10^9^/L or <5 × 10^9^/L, CRP > 50 mg/L, pneumatosis intestinalis, and ascites (Training Set). **(B)** Panel (Nomogram): Hypothermia, absent bowel sounds, WBC > 20 × 10^9^/L or <5 × 10^9^/L, CRP > 50 mg/L, pneumatosis intestinalis, and ascites (Validation Set). **(C)** Panel (Nomogram): PVG, IMV, PLT, pH < 7.35 (Training Set) **(D)** Panel (Nomogram): PVG, IMV, PLT, pH < 7.35 (Validation Set) **(E)** Panel of biomarkers: IL-8, IL-24, CCL20. **(F)** Panel of biomarkers: A2ML1, CD14, CST3, PEDF, RET4, VASN **(G)** Prediction model using preexisting data for training. **(H)** Prediction model using preexisting data for validation. **(I)** Machine learning model with training dataset (all features included). **(J)** Machine learning model with training dataset (abdominal radiographs only) **(K)** Machine learning model with training dataset (clinical parameters only) **(L)** Machine learning model with validation dataset (all features included). **(M)** Modified Albumin (MA). **(N)** Panel of biomarkers: HBD-2 and Claudin 3. **(O)** RELM-b with Abdominal Tenderness or Guarding.

*At the day of diagnosis* (Figure 6): Biomarkers like MCP-4 and IL-8 demonstrate high sensitivity, whereas CCL20, OPG, RELM-b, I-FABP, and L-FABP recorded high specificity values. Finally, combination panels involving biomarkers such as HBD-2 and Claudin-3 demonstrated moderate sensitivity and specificity values, while a panel constituting PVG, IMV, PLT, and pH 7.35 demonstrated higher specificity.

Two studies have reported poor discriminatory power for IL-6 with AUC values ranging from 0.593-0.695, while one study reports promising results with AUC 0.931 and sensitivity 91.7%. For IL-8, although two studies report conflicting results in terms of the specificity (88.2% vs. 59.2%), both studies do report a good discriminatory power with AUC values ranging from 0.778-0.82).

In the non-specific biomarker category, high AUC values are found for novel biomarkers like A2ML1 (0.804 with 80% sensitivity and 70% specificity), PEDF (0.839, 80% sensitivity and 68% specificity), and RET 4 (0.81 AUC with 70% sensitivity and 81% specificity). Furthermore, studies have also reported the value of using scores such as MDAS (AUC 0.77, 70% sensitivity and 66% specificity) and SNAPPE II (AUC 0.71, 70% sensitivity and 60% specificity) to discriminate between stage II & III.

Within the gut-specific marker category, a panel constituting HBD-2 and Claudin-3 demonstrated excellent sensitivity (90%) and specificity (80%) with AUC 0.805, while RELM-b also reported a promising diagnostic profile (AUC 0.943, 82.6% sensitivity and 92.3% specificity).

Within the acute phase reactants category, biomarkers such as platelets used in combination with SAA report AUC of 0.93 with 94% sensitivity and 83% specificity. Neutrophils (AUC 0.66, with 75% sensitivity and 60% specificity) and white blood cells (AUC 0.767 with 78% sensitivity and 67% specificity) maintain a moderate discriminatory profile in terms of differentiating between medical and surgical NEC cases. Moreover, other haematological markers like Fibrinogen (AUC 0.74, 74% sensitivity and 60% specificity) and Sodium (AUC 0.875, 84% sensitivity and 80% specificity) have also been reported.

**Figure 7:**
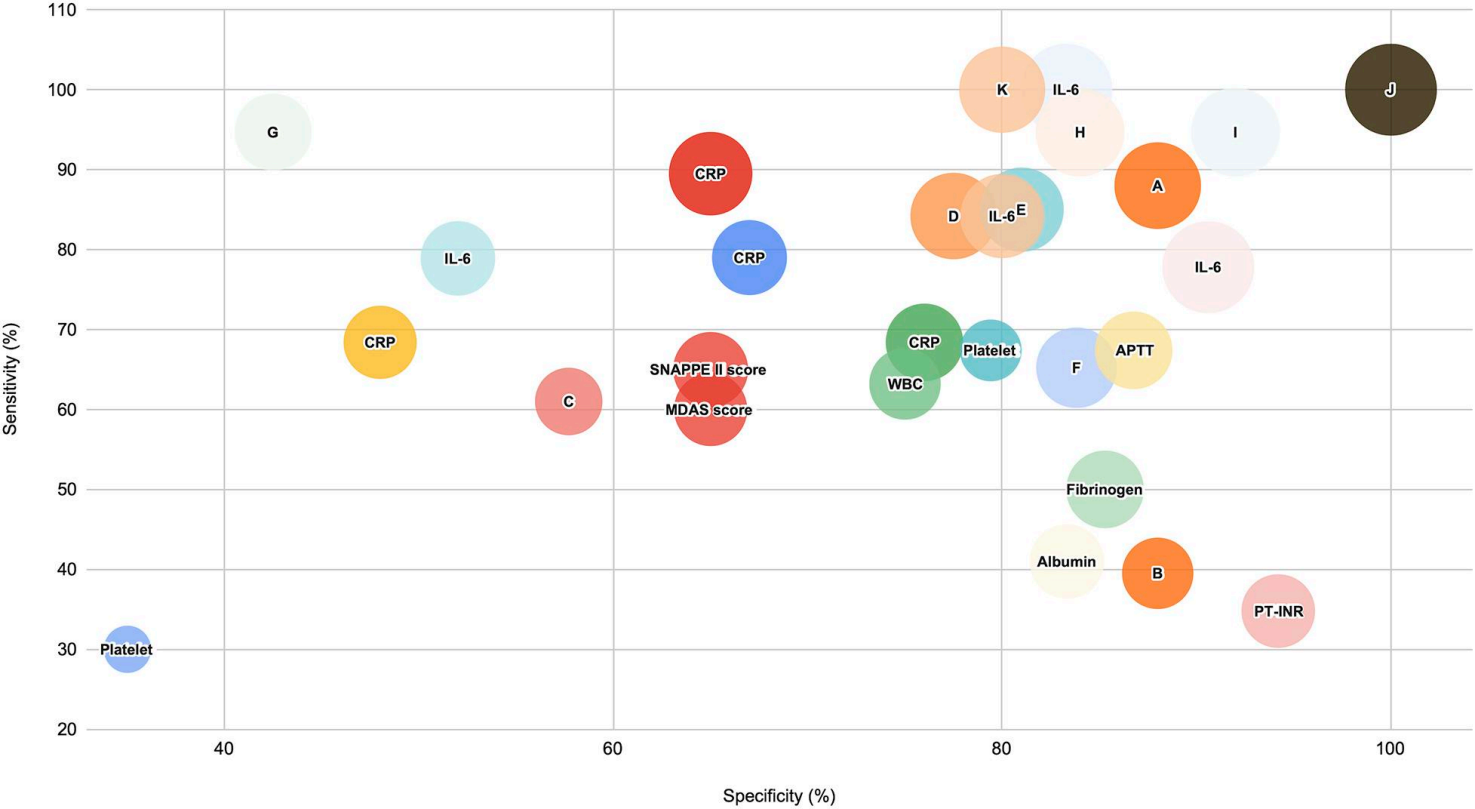
Biomarker performance to differentiate medical NEC (stage II) from surgical NEC (stage III) after diagnosis. This figure presents the performance of biomarkers in distinguishing NEC Stage III (surgical NEC) from NEC Stage II (medical NEC) across molecular biomarker categories. Each biomarker is evaluated based on sample timing, sensitivity, specificity, and confidence intervals, highlighting effectiveness in discrimination post diagnosis. **(A)** Panel involving: Ultrasound markers, respiratory and hemodynamic instability, abdominal wall cellulitis, and C-reactive protein > 16 mg/L. **(B)** Platelet to Lymphocyte Ratio **(C)** Absolute Neutrophil Count. **(D)** Neutrophil to Lymphocyte Ratio. **(E)** Panel of biomarkers: WBC and Platelet. **(F)** Prothrombin **(G)** Pre-albumin. **(H)** Modified Albumin from one study. **(I)** Modified Albumin from a second study. **(J)** FTOE (Fractional Tissue Oxygenation Extraction) **(K)** rSO2 (regional tissue Oxygen Saturation).

*Post diagnostic period* (Figure 7): IL-6 recorded a high sensitivity but moderate specificity value for this time period, followed by scoring methods such as MDAS and SNAPPEE II, depicting moderate sensitivity and specificity, while Modified Albumin and Activated Thromboplastin Time (APTT) offered higher specificity with a moderate sensitivity.

CRP did not demonstrate a very promising profile post diagnosis, with AUC values ranging from 0.67 to 0.85 coupled with weaning specificity values between 48% and 76%. However, using CRP in combination with other factors such as hemodynamic instability and abdominal wall cellulitis does improve the discriminatory profile (AUC 0.89). In the haematological indices category, Neutrophil to Lymphocyte ratio has a good diagnostic value (AUC 0.886, 84.2% sensitivity and 77.5% specificity) one day after diagnosis, in contrast to Platelet to Lymphocyte ratio, which did not present a very strong diagnostic profile (AUC 0.61 with 39.5% sensitivity and 88% specificity).

Where clotting factors are considered, APTT (0.715, 67.39% sensitivity, 86.76% specificity) outperforms prothrombin time (AUC 0.769 with 65.2% sensitivity and 83.82% specificity) in terms of discriminating between the two types of NEC. For other non-specific biomarker categories, modified albumin (MA) reports good biomarker activity level on the 3rd (AUC 0.93 with 94.7% sensitivity and 92% specificity) and 7th day (AUC 0.935, 94.7% sensitivity and 92% specificity) post diagnosis.

**Figure 8:**
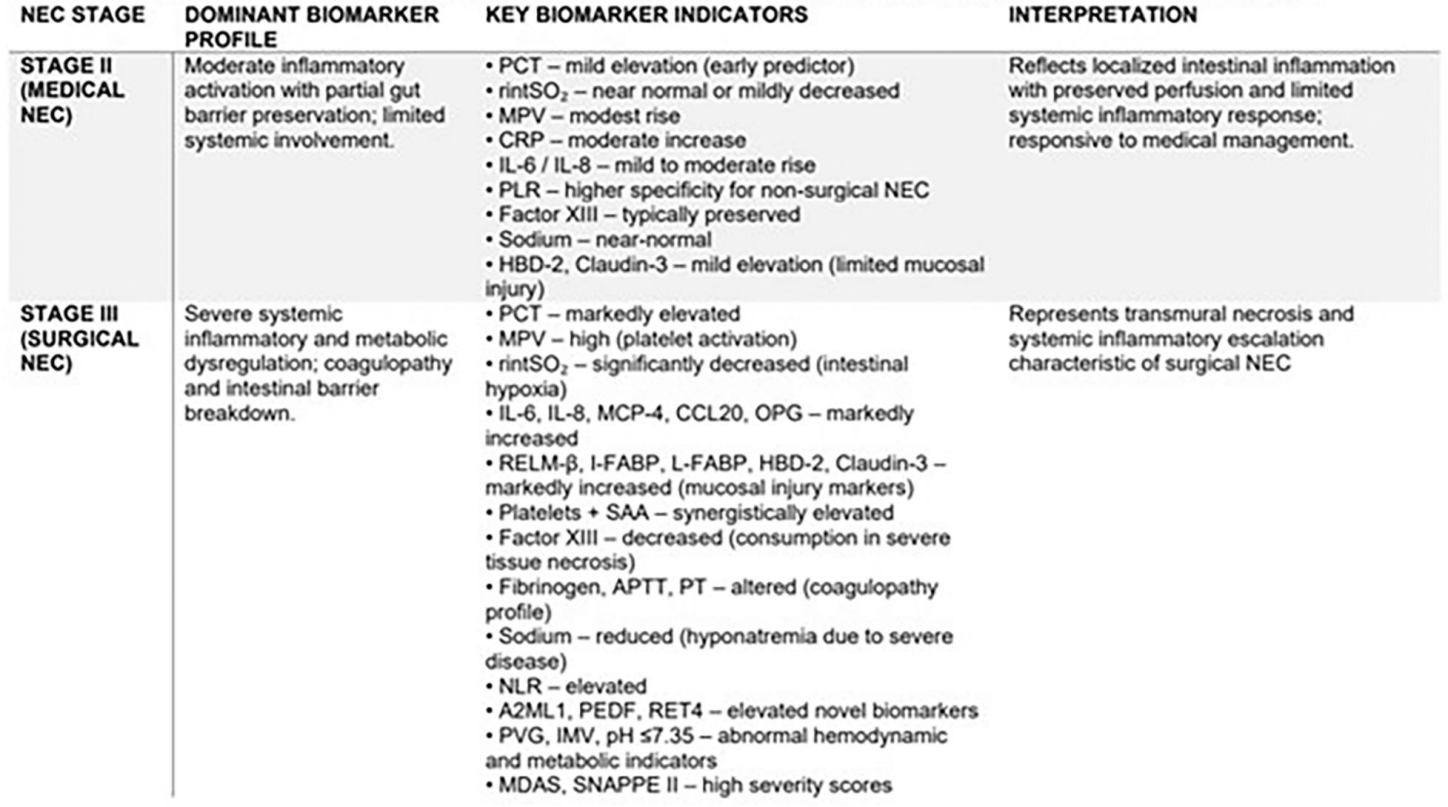
Stage-Oriented summary trends: biomarker profiles in NEC stage II (medical) or stage III (surgical). This figure provides a concise summary of the discussed biomarkers, and what their significance/ applicability may be in the context of differing between stage II & stage III NEC.

Please find Figure 8 below, demonstrating a summary of biomarker trends in NEC stages II & III. Also, Supplementary Table S1.2, summarising the above biomarker profiles in the context of their trend, timing & molecule type.

## Discussion

While a high diagnostic accuracy for already established biomarkers such as S100A8/A9, L-FABP, SAA, and IL-33 was reported here, the review also highlighted novel biomarkers such as CD64+, TGF-beta 1, Urinary caveolin-1, and Volatile Organic Compounds (VOCs), among others.

### NEC biomarkers

#### Haematological indices

Neutrophils, specifically CD64+, have been shown to provide excellent diagnostic and early screening ability and a remarkable sensitivity. Their use in accurately identifying NEC is further complemented by using the Neutrophil/Lymphocyte Ratio. Necrotising Enterocolitis involves a whole plethora of inflammatory responses, and many studies have identified the critical role played by inflammatory key players such as neutrophils and lymphocytes in this condition (29–31). In terms of differentiating between the two stages of NEC, our review identified a novel biomarker, Factor XII,I with a very high AUC (0.958) for the pre-diagnostic period. Factor XIIIa is reported to be involved in the process of linking fibrinogen-*γ* dimers, which serves as the last step towards clot formation. In the absence of transglutaminase Factor XIII, the clot undergoes premature breakdown, resulting in profuse bleeding (26). Hence, monitoring the levels of this factor can not only help identify the advanced stages of NEC, but it can also serve as a therapeutic target (26). In this review, a risk model suggested by Chen et al. (32) comprising characteristic signs such as Portal Venous Gas (PVG), IMV (Invasive Mechanical Ventilation), PLT (Platelet count), and pH below 7.35 shows a promising diagnostic profile.

#### Cytokines and chemokines

A panel consisting of IL-8, IL-24 and CCL20, have been shown to perform quite well during the diagnostic phase of NEC. For post-diagnosis, markers such as IL-33 and TGF-beta were the optimal candidates. In terms of differentiating between stages of NEC, CCL20, either used by itself or as a panel, demonstrated an ideal diagnostic profile, while IL-6 was more suited for monitoring disease progression CCL20 is predominantly expressed in intestinal epithelium against an inflammatory response, and the levels of CCL20 expressed in intestinal epithelium and may have utility to differentiate between stages of NEC (33). IL-24 is a proinflammatory, proapoptotic cytokine belonging to the IL-10 family (14). Although its role has been investigated in other intestinal conditions like IBD (34), and Coeliac Disease (35), the role of IL-24 towards the pathogenesis of NEC still needs to be further investigated (14).

### Acute phase reactants

Acute Phase Reactants are proteins whose plasma concentrations increase or decrease during inflammation, infection, or tissue injury. Since NEC is an inflammatory process, there is promise in terms of using these molecules to diagnose NEC or monitor disease progression. Although the role of some classic biomarkers such as CRP have been well established in literature, we have highlighted how its use can be manifested as a diagnostic marker between NEC and other conditions This is in keeping with the reported literature about high levels of intervariability and intravariability between Calprotectin levels, making it a very non specific diagnostic marker for NEC For disease risk stratification and early identification. In contrast, Calgranulin-C (S100A12) demonstrated a promising profile to distinguish NEC III from other similar presentations, such as Sepsis (36). S100A12 belongs to the damage-associated molecular pattern proteins that are released by damaged cells under stressful conditions, such as those seen in Necrotising Enterocolitis (36). As opposed to Calprotectin, Calgranulin-C has been shown to be resistant to biodegradation by fecal bacteria, thereby pointing to its potential use as a biomarker for diagnosing and discriminating between the stages of NEC (36).

### Enhanced Non-specific markers

Although faecal proteins are themselves non-specific biomarkers of inflammation, the nature of the specimen in which they are found, i.e., stool, gives us greater clues as to the site of tissue injury, and so these biomarkers are enhanced. Enhanced non-specific biomarkers are especially relevant to NEC because their presence alone indicates increased intestinal mucosal permeability and/or disruption of mucosal integrity (12). The gut microbiome is the main catalyst in the process of colonic fermentation that produces VOCs (37). Fewer VOCs are found in the gut of neonates, suggesting the relative simplicity of their gut flora. There are changes in the patterns of colonisation leading up to NEC, which is reflected in a decrease in the quantity of VOCs (37). This can potentially be used to detect those at risk earlier. The potential utility of volatile Organic compounds has been documented in this review as well. For instance, Pent-1.ene.3-one recorded for its pre-diagnostic use, while another TCA metabolite, L-malic acid, exhibited an excellent pre-diagnostic profile with high sensitivity. On the day of diagnosis, a novel marker RIPK3 demonstrated a promising candidate profile with very high sensitivity. Receptor-interacting-protein-kinase-3 (RIPK3) is a key player in a regulated cell death pathway called “Necroptosis” by phosphorylating mixed lineage kinase domain (38) (MLKL). Although apoptosis has been largely agreed to be responsible for the intestinal cell death seen in NEC, a vast majority of effects evident in NEC can still not be explained by apoptosis alone (39). Murine and animal models have revealed cell death in intestinal villi instead of just the crypts (39). At the same time, surgical specimens have also confirmed the role of necroptosis in NEC (39). Using this biomarker in conjunction with other established markers, such as CRP and Lactic Acid, further adds to the diagnostic accuracy.

For the pre-diagnostic period, Lipocalin-2 demonstrated 70% sensitivity to differentiate advanced NEC (Stage III). While on the day of diagnosis, a panel composed of amino acids Tyrosine, Arginine, and Riboflavin recorded a high AUC value, which points to their use in the diagnosis of NEC. The proteomic panel markers like A2ML1 reported in our results are a part of the coagulation cascade, and evidence exists with regard to the role of coagulation necrosis in NEC resection specimens (27, 40). CD14 is a pattern recognition receptor molecule that initiates an innate immune response against bacterial LPS, which has been seen in NEC III cases (40). Consistent with these findings, our results report a better discriminatory power for these biomarkers, which likely suggests their role in deciding between conservative or surgical treatment courses for NEC patients. Endocan is another molecule identified in this category as a highly sensitive biomarker with perfect diagnostic accuracy for NEC at the onset of symptoms.

### Gut-Specific markers

Specific biomarkers for intestinal mucosal injury and inflammation have been the primary focus of recent research. This category of “gut-specific” biomarkers comprises proteins that are exclusively or predominantly produced by cells lining the bowel lumen. I-FABP is solely synthesised by enterocytes, and L-FABP is expressed mainly by enterocytes or hepatocytes (41). TFF3 is predominantly expressed in the intestinal goblet cells and mucin-producing epithelial cells (41). Based on the studies recorded, I-FABP has been shown to consistently perform well in terms of its screening and diagnostic ability prior to the day of diagnosis, and a promising profile when used as a panel with TFF-3 on the day of diagnosis. Another molecule belonging to the Resistin family of proteins also demonstrated an optimistic diagnostic profile. For the post-diagnostic period, Galectin-4, another novel molecule, has been identified with high specificity towards monitoring the progression of NEC through its advanced stages.

However, it would appear that while these markers provide vital information in the setting of advanced NEC (stage III), they are unlikely to be used as an early screening tool or diagnostic biomarker for mild cases. This is because the degree of mucosal damage needed to release an adequate quantity of these proteins to significantly raise their concentrations in either serum or urine is simply too great to be of use in early or less severe cases of NEC (12).

### Novel biomarker approach

Human-beta-defensin-2 protein (HBD-2) belongs to the defensin family of cysteine rich amphipathic polypeptides that are preferentially expressed in response to inflammatory response triggered by inflammatory cytokines (42). Various studies have pointed to their potential role in inflammatory processes such as Crohn's disease (43). In the same thread, Claudin-3 forms an important component of tight junctions that constitute intestinal epithelial permeability and exchange (43). Experiments on rat models infected with NEC have revealed heightened Claudin expression levels, thus pointing towards its potential use as a diagnostic biomarker (43). Resistin is a cytokine that promotes an inflammatory response, and its expression is significantly increased in sepsis and septic shock. RELMb maintains the functional barrier protein of the gastrointestinal tract, offering protection (9). Recent evidence suggests that RELMb is capable of recruiting macrophages to produce proinflammatory mediators such as TNF-alpha and IL-15 (9). Consistent with this literature, our findings regarding improved RELMb diagnostic accuracy with Platelets during the first 6 h of suspected NEC may point to its potential role as an early marker for NEC diagnosis.

The evidence supports receipt of the mother's own breast milk as the most protective factor against NEC in preterm infants. However, infants receiving this still developed NEC, suggesting a variance in the composition of the breast milk may play a role in its protective qualities (17). DSNLT in particular was found to be associated with reduced incidence of NEC development and improved NEC-associated mortality rate (17). While it is still early to comment on the diagnostic role of this human oligosaccharide, it is worth mentioning that HMO may be a promising candidate for early detection of NEC since it demonstrated characteristics of an ideal biomarker. Lastly, Inter-alpha inhibitor protein (IaIp) belongs to a family of serine protease inhibitors that are known to combat the damaging effects of proteases during inflammation by various mechanisms, such as binding to the extracellular, damage-inducing histone proteins (44). The identity and potential use of IaIp has been documented in other conditions such as Dengue and neonatal seps**is (**44)**.**

### Artificial intelligence and machine learning models

Lin et al. (51) suggested a machine learning based NEC prediction system using microbiome data obtained from sequencing DNA from infant stool samples, and the results have been very promising (sensitivity 86% specificity 90%). In the same thread, the model suggested by Gao and colleagues (52), incorporating radiological and clinical features to diagnose NEC and to distinguish it from advanced stages, has shown promising results (AUC 0.98, 100% sensitivity and 86% specificity). The added advantage of incorporating these models is that the data set for training can be updated based on evolving clinical needs (45). However, the overall applicability and generalisability of these models might be hindered by the fact that there is no universally accepted definition of NEC, which may lead to variations in diagnosing NEC or distinguishing it between advanced stages based on individual institution guidelines (45). Moreover, these models can also encounter biases in the input data and its interpretation (45). Lastly, these models can struggle to cope with missing data, which lends greater responsibility to clinicians to exclude a few features, or input data manually by deciding the best way to fill in each gap (45). Subsequently, this can induce bias and likely skew the prediction basis of the model (45).

While these models demonstrate strong internal performance, most remain exploratory, with validation limited to small, single-centre cohorts, raising concerns about overfitting and limited generalisability. To advance towards clinical adoption, multicentre external validation and harmonisation of input variables are essential. Successful deployment will also require seamless integration with neonatal electronic health records, interpretable model outputs for clinicians, and adherence to regulatory frameworks. These challenges underscore that although ML/AI approaches are promising, they are not yet ready for routine use in NEC diagnosis and risk stratification.

Additionally, the efficacy of using scoring systems—such as the Metabolic Derangement Acuity Score (MDAS), and Scores for neonatal-acute-physiology-perinatal-extension (SNAPE-II)—has also been included in this review. While these systems can provide a structured approach towards clinical decision making, their diagnostic accuracy represented a low profile in our review. This points to the efficacy of using them alongside other diagnostic tools and with sound clinical judgement. This area still needs to be tapped with further studies and literature to be added in order to investigate the effectiveness of using these scoring systems (46).

### Limitations of studies

The results of the QUADAS-2 assessment revealed a high risk of bias in most studies, particularly within the “patient selection” domain. This was largely attributable to the lack of consecutive sampling, reliance on retrospective and case–control designs, and the inclusion of controls that may overestimate diagnostic accuracy. Additional sources of bias included insufficient reporting of blinding methods for both the index test and the reference standard (diagnosis of NEC based on Bell's criteria).

Significant concerns about the applicability of the studies arose from the inclusion of neonates without risk factors or signs and symptoms of NEC, and from the inclusion of target conditions other than Bell stage II/III NEC. Several studies, particularly the case–control studies, recruited term neonates, healthy neonates, and neonates diagnosed with NEC at the time of enrolment. The incorporation of neonates without risk factors (e.g., preterm, low birth weight) or signs and symptoms of NEC is likely to skew the clinical characteristics of the study population to be less representative of the clinical population at-risk for NEC, and raises the risk of bias. Furthermore, the inclusion of target conditions other than NEC is also likely to skew the data, with there being significant overlap between NEC Stage I and conditions such as SIP, septic ileus, and sepsis—meaning that, as per these studies, those conditions can often be diagnosed under the criteria of NEC Stage I (47).

These issues surrounding the diagnostic criteria for NEC Stage I have, in some studies, resulted in the usage of diagnostic criteria other than Bell's criteria, which remains the diagnostic “gold-standard” for NEC. Heath et al. (23) constructed a criteria to differentiate patients with NEC from those with focal or spontaneous intestinal perforation (SIP).

Some minor applicability concerns were noted due to inconsistencies between the use of either Bell's criteria or (Kliegman's) modified Bell's criteria. Studies where NEC diagnosis was based on ultrasonographic findings could potentially lead to errors in terms of interpretation. Inconsistencies between the timing of sampling (e.g., at time of diagnosis vs. at onset of symptoms) also raised concerns with the applicability and validity of the data as symptom recognition is often a very subjective judgement.

Moreover, there was a lack of complete accuracy data (i.e., AUC values, sensitivity, specificity, negative and positive predictive values) provided by the studies. Although the included studies had a fair sample size, the applicability of the data still warrants caution, as it is difficult to generalise the results to a widespread population on a larger scale. Furthermore, many of the included studies used ELISA for the biomarker assay. While this assay is highly efficient, simple to operate, and reports higher sensitivity and specificity, it has limitations such as a high possibility of false positives/negatives due to insufficient blocking of immobilised antigens (48). More importantly, there was widespread variation in definitions used to classify different stages of NEC.

### Limitations of the review

Limitations of this review include the exclusion of studies that did not fit our eligibility criteria and the lack of any meta-analysis. The exclusion of secondary research, specifically meta-analyses, meant that relevant papers reporting statistical analyses of biomarker accuracy were not incorporated in this review. Similarly, due to a high level of heterogeneity between studies and the limited amount of data for each biomarker, we were unable to perform a meta-analysis.

For this review, we only included studies with NEC Bell's Stage II and/or III (definite and advanced NEC) as target conditions and reporting accuracy data on biomarkers. Current clinical trials and cohort studies vary in their inclusion of NEC Stage I, or “preterm NEC”. Although our decision to restrict the target conditions was made to increase comparability between studies, a notable portion of studies were excluded, such as those that included NEC I within their population but did not report independent accuracy data for their NEC Bell Stage II and/or III groups, limiting our sources for biomarker data.

Similarly, several studies assessing diagnostic biomarkers identified by genome-wide expression studies (GWAS) or by a metabolomic/proteomic approach were excluded as limited data were reported on their accuracy and performance characteristics. Nonetheless, we included a few studies that incorporated a biomarker validation phase into their methodology and reported data on biomarker accuracy. However, the results have not been promising and warrant further research.

Across our review, AUC was used as a metric of biomarker performance. The AUC provides an aggregate measure of diagnostic accuracy, however it does not reflect the threshold-dependent balance between biomarker sensitivity and specificity. In clinical practice, applying such biomarkers without taking into account the clinical context could lead to overdiagnosis and unnecessary intervention where a biomarker with a high sensitivity is measured (such as MPV, S100A12). Conversely, relying on highly-specific biomarkers such as RELM-β or Claudin-3 may, where yielding a negative result, lead to missing early or evolving disease. Thus the clinical applicability of each biomarker should rely on not just the AUC value, but also on how sensitivity and specificity thresholds align with intended use.

While including positive and negative predictive values (PPV & NPV) would certainly add to the clinical relevance of these findings, this was beyond the scope of this systematic review. PPV & NPV calculations depend heavily on disease prevalence and study population characteristics, which varied widely and were often not reported in the included studies. In this context, calculating PPV & NPV may have produced misleading or non-generalisable results. Future reviews or studies which use pooled or individual patient-level data may be better positioned to explore this data, which would further analyse the real-world applicability of these biomarkers in NEC.

Lastly, our review focused on the clinical properties of NEC biomarkers, focusing on parameters such as accuracy, the ability to discriminate between NEC Bell Stage II and III, and the ability to discriminate between NEC and other neonatal intestinal pathologies. As such, we did not evaluate laboratory properties such as cost, availability in clinical laboratories, turnaround time, and stable time window for specimen collection, which remain crucial factors when evaluating “ideal” biomarkers (49).

## Conclusion and future directions

Our literature review highlights the potential of both established and emerging biomarkers for diagnosing NEC, including TGF-*β*1, IL-33, L-FABP, SAA, Calgranulin-C, volatile organic compounds (VOCs), Gal-4, and others. We also reviewed studies evaluating the accuracy of artificial intelligence and machine learning in diagnosing NEC, distinguishing it from similar conditions, and differentiating its advanced stages. Although these biomarkers demonstrate promising diagnostic accuracy, further validation is needed. A combined biomarker panel approach—integrating multiple biomarkers into diagnostic algorithms or scoring systems—could improve the accuracy of NEC diagnosis and stage differentiation. Importantly, there is an urgent need for a consensus definition to standardise the description of NEC stages, enabling meaningful comparisons across studies investigating biomarker utility.

In addition to validating individual biomarkers, future research must address key limitations in current studies. Small sample sizes and the retrospective or case-control design prevalent in many NEC biomarker studies hinder the ability to draw robust conclusions. To improve research outcomes, future studies should employ larger cohort designs and adhere to standardised guidelines for conducting and reporting NEC biomarker research. This approach would enhance methodological quality, facilitate meaningful comparisons, and enable more reliable synthesis of findings through meta-analyses. Moving forward, integrating genomic, metabolomic, and proteomic approaches, coupled with thorough validation in clinical settings, holds significant promise for advancing NEC diagnostics.

## Data Availability

The original contributions presented in the study are included in the article/Supplementary Material, further inquiries can be directed to the corresponding author.
