# Supplementary material for: Necrotising enterocolitis biomarkers: a systematic review
Source: Front Pediatr. 2026 Jan 12;13:1652566. doi: 10.3389/fped.2025.1652566 (PMC12833235; doi:10.3389/fped.2025.1652566)
Supplement: Supplementary file 1 [file Table1.docx]

**Supplementary Table 1.1: Summary of Study Characteristics and Key Findings for NEC Biomarker Evaluation including all biomarkers**

| **Biomarker** | **Reference** | **Study design** | **Study Population** | **Characteristics of Controls** | **Target Condition vs Control** | **Timing of Sampling** | **Cut-off Value (units)** | **AUC (95% CI)** | **Sensitivity (%)** | **Specificity (%)** |
| --- | --- | --- | --- | --- | --- | --- | --- | --- | --- | --- |
| **Platelets** | Feng et al. (2022) | Retrospective | N=114 preterm and term neonates: 68 NEC II (medical treatment) and 46 NEC III (surgical intervention) cases. | n=68 suspected NEC II or NEC III receving medical treatment only | NEC II vs NEC III | Post diagnosis (12 hr after) | 252 (x10^9/L) | 0.763 | 67.39 | 79.41 |
|  |  |  |  |  |  |  |  |  |  |  |
|  |  |  |  |  |  |  |  |  |  |  |
|  |  |  |  |  |  |  |  |  |  |  |
|  |  |  |  |  |  |  |  |  |  |  |
|  |  |  |  |  |  |  |  |  |  |  |
|  |  |  |  |  |  |  |  |  |  |  |
|  |  |  |  |  |  |  |  |  |  |  |
|  | Dong et al. (2023) | Prospective Case Contol | n=88, Preterm Neonates with 30 NEC cases (including NEC II (18) and NEC III (12), 29 Sepsis, and 29 controls, BW<2500g, GA<37weeks | n=30 Pretem Neonates with NEC II (18) or NEC III (10) | NEC II vs NEC III | Diagnostic | 237.5 X10^9/L | 0.808 (0.645–0.970) | 91.7 | 55.6 |
|  |  |  |  |  |  |  |  |  |  |  |
|  |  |  |  |  |  |  |  |  |  |  |
|  |  |  |  |  |  |  |  |  |  |  |
|  |  |  |  |  |  |  |  |  |  |  |
|  |  |  |  | n=29 Premterm Healthy neonates without NEC or other infectious disease | NEC II/III vs Controls |  | 241.5 x 10^9/L | 0.682 (0.540–0.824) | 79.3 | 66.7 |
|  | Luo et al. (1) | Case Control | n=58, Preterm infants | n=29, preterm infants matached for GA and GW | NEC II/III vs Controls | Diagnostic | 378.3ng/L | NA | 69.34 | 82.87 |
|  | Yu et al. (2) | Retrospective | n=84 Preterm and Term Neonates with 43 NEC II and 41 NEC III cases | n=84 Preterm and Term Neonates with 43 NEC II and 41 NEC III cases | NEC II vs NEC III | Post-diagnosis | NA | 0.31 (0.21–0.41) | NA | NA |
|  | Benkoe Reck et al. (12) | Retrospective | n=113, Preterm infants diagnosed with NEC (and had undergone medical/surgical treatment) | n=63, infants with Bell Stage II NEC | NEC II vs NEC IlI | Diagnostic | NA | 0.585 (0.47-0.70) | NA | NA |
|  | Reisinger et al. (3) | Cohort | n=29, Preterm infants with 13 NEC II and 16 NEC III cases, BW<1500g, GA<32 weeks | n=13 Preterm infants with diaognosed NEC II | NEC II vs NEC III | Diagnostic | <273×10^9 cells/l) | 0.75 (0.57–0.94) | 76 | 83 |
|  |  |  |  |  |  | Diagnostic followed by surgery | <267×10^9 cells/l) | 0.78 (0.61–0.96) | 76 | 83 |
| **Mean Platelet Volume (MPV)** | Meng et al. (4) | Retrospective Cohort | n=122 Preterm Neonates with 79 NEC I/II cases and 43 NEC III cases | n=79 Preterm infants with NEC I/II | NEC II vs NEC III | Pre-diagnostic | 9.395 (fL) | 0.85 | 96.2 | 32.6 |
| **Platelet to Lymphocyte ratio (PLR)** | Guo et al. (2024) | Retrospective | n=90, Preterm Infants with 52 NEC II cases and 38 NEC III cases, GA<37weeks | n=52 Preterm infants diagnosied with NEC II | NEC II vs NEC III | Post-diagnosis | 271.53 | 0.61 (0.48–0.73) | 39.5 | 88 |
| **Plasma Anti-Myosin Autoantibodies** | Chen et al. (5) | Prospective Cohort | n=38 Preterm neonates with 17 NEC I, 11 NEC II, 10 NEC III cases, and 13 controls, GA<32weeks, BW<1500g | n=13 GA and BW matched Healthy preterm infants without NEC | NEC vs Controls | Pre-diagnostic | 14.68 ng/ml | 0.8856 | 81.58 | 76.93 |
| **Plasma Anti-Myosin Autoantibodies** | Chen et al. (5) | Prospective Cohort | n=38 Preterm neonates with 17 NEC I, 11 NEC II, 10 NEC III cases, and 13 controls, GA<32weeks, BW<1500g | n=13 GA and BW matched Healthy preterm infants without NEC | NEC II vs Controls | Pre-diagnostic | NA | 0.83 (0.6697 - 0.9947) | NA | NA |
|  |  |  |  |  | NEC III vs Controls |  | NA | 0.84 (0.6824 -1.000) | NA | NA |
| **Absolute Monocyte Count (AMC)** | Moroze et al. (6) | Retrospective | n=130, Preterm Infants with 53 NEC Cases (including NEC I (20), NEC II (23), NEC III (10)), 10 SIP, 10 Dead post enrollment, and 7 Post cx), GA<32 weeks and BW<1100g | n=27 Preterm Neonates wtihout NEC | NEC vs Controls | Post-diagnostic (d=3) | NA | 0.57 | 42 | 78 |
|  |  |  |  | n=27 Preterm Neonates wtihout NEC | NEC II/III vs Controls | Post-diagnostic (d=3) | NA | 0.68 | 67 | 66 |
|  |  |  |  | n=20 Preterm Neonates with NEC I | NEC II/III vs NEC I | Post-diagnostic (d=3) | NA | 0.788 | 85 | 65 |
|  | Desiraju et al. (7) | Retrospective | n=143, Preterm infants with 76 NEC caes (including NEC II (61) and NEC III (15), 38 Bacteremia, and 29 Healthy controls, GA<33weeks | n=61 NEC II diagnosed cases | NEC II vs NEC III | Pre-diagnostic | NA | 0.83 | 73 | 87 |
|  |  |  | n=143, Preterm infants with 76 NEC caes (including NEC II (61) and NEC III (15), 38 Bacteremia, and 29 Healthy controls, GA<33weeks | n=29 Preterm Healthy infants | NEC II/III vs Controls |  | NA | 0.81 | 51 | 93 |
|  | Remon et al. (8) | Retrospective | n=326, VLBW infants | n=257, infants matched for GA and BW presenting with Feeding intolerance without signs of NEC | NEC II/III vs Controls | Pre-diagnostic (d=3.5) | 0.8 | 0.76 (0.69-0.83) | 70 | 71 |
| **White Blood Cell Count (WBC)** | Dong et al. | Prospective Case Contol | n=88, Preterm Neonates with 30 NEC cases (including NEC II (18) and NEC III (12), 29 Sepsis, and 29 controls, BW<2500g, GA<37weeks | n=29 Premterm Healthy neonates without NEC or other infectious disease | NEC II/III vs Controls | Diagnostic | 8.27 x 10^9/L | 0.720 (0.580–0.860) | 86.2 | 63.3 |
|  | Yang et al. (9) | Cross-sectional | n=161 Preterm Neonates with 41 NEC I, 34 NEC II, 28 NEC III cases, and 58 controls, GA<34 weeks | n=34 Preterm Neonates with NEC II | NEC II vs NEC III | Post-diagnostic (d=1) | NA | 0.612 (0.432-0.792) | 63.2 | 75 |
|  |  |  |  |  | NEC II/III vs Controls |  | NA | 0.574 (0.453-0.694) | 96.5 | 26.8 |
| **White Blood Cell Count (WBC)** | Yu et al. (2) | Retrospective | n=84 Preterm and Term Neonates with 43 NEC II and 41 NEC III cases | n=84 Preterm and Term Neonates with 43 NEC II and 41 NEC III cases | NEC II vs NEC III | Post diagnosis | NA | 0.18 (0.10–0.26) | NA | NA |
| **Neutrophil** | Dong et al. (2023) | Prospective Case Contol | n=88, Preterm Neonates with 30 NEC cases (including NEC II (18) and NEC III (12), 29 Sepsis, and 29 controls, BW<2500g, GA<37weeks | n=29 Premterm Healthy neonates without NEC or other infectious disease | NEC II/III vs Controls | Diagnostic | 58.85 (%) | 0.730 (0.585–0.876) | 96.6 | 60 |
|  | Xaio Chen et al. (2022) | Prospective | n=60, Preterm and Term neonates with 33 NEC II and 27 NEC III cases | n=33 Preterm and Term neonates with NEC II | NEC II vs NEC III | Diagnostic | NA | 0.66 | 75 | 60 |
| **Absolute Neutrophil Count (ANC)** | Guo et al. (2024) | Retrospective | n=90, Preterm Infants with 52 NEC II cases and 38 NEC III cases, GA<37weeks | n=52 Preterm infants diagnosied with NEC II | NEC II vs NEC III | Post diagnosis (d=1) | 3.17 × 10^9 (/microL) | 0.55 (0.42–0.67) | 61 | 57.7 |
| **Neutrophil CD64+** | Lam et al. (2013) | Prospective Cohort | n=218 VLBW neonates with 33 proven sepsis/NEC cases, 22 clinical sepsis/NEC cases, 100 non-sepsis/non-NEC, and 63 asymptomatically activated controls | n=163 Preterm Neonates without NEC/Sepsis | NEC II/III vs Controls | Pre-diagnostic | 5655 antibody-PE molecules bound/cell (PE units) | 0.95 | 89 | 98 |
|  |  |  |  |  |  | Diagnostic | 5655 antibody-PE molecules bound/cell (PE units) | 0.93 (0.88–0.99) | 89 | 95 |
| **Neutrophil to Lymphocyte Ratio (N/L)** | Yang et al. (9) | Cross-sectional | n=161 Preterm Neonates with 41 NEC I, 34 NEC II, 28 NEC III cases, and 58 controls, GA<34 weeks | n=58 Preterm Neonates without NEC | NEC II/III vs Controls | Post-diagnostic (d=1) | NA | 0.812 (0.727-0.897) | 70.1 | 82.9 |
|  |  |  |  | n=34 Preterm Neonates with NEC II | NEC II vs NEC III |  |  | 0.886 (0.790-0.983) | 84.2 | 77.5 |
| **Prothrombin Time (PT)** | Feng et al. (2022) | Retrospective | n=114 Preterm and Term Neonates with 68 NEC II/ Medical Treatmet and 46 NEC III/Surgical intervention cases | n=68 suspected NEC II or NEC III receving medical treatment only | NEC II vs NEC III | Post-diagnostic (d=0.5) | 16.7(s) | 0.769 | 65.22 | 83.82 |
| **International Normalized Ration (PT-INR)** | Feng et al. (2022) | Retrospective | n=114 Preterm and Term Neonates with 68 NEC II/ Medical Treatmet and 46 NEC III/Surgical intervention cases | n=68 suspected NEC II or NEC III receving medical treatment only | NEC II vs NEC III | Post-diagnostic (d=0.5) | 1.23 | 0.647 | 34.78 | 94.2 |
| **Activated Partial Thromboplastin Time (APTT)** | Feng et al. (2022) | Retrospective | n=114 Preterm and Term Neonates with 68 NEC II/ Medical Treatmet and 46 NEC III/Surgical intervention cases | n=68 suspected NEC II or NEC III receving medical treatment only | NEC II vs NEC III | Post-diagnostic (d=0.5) | 41.3 (s) | 0.715 | 67.39 | 86.76 |
| **Fibrinogen** | Feng et al. (2022) | Retrospective | n=114 Preterm and Term Neonates with 68 NEC II/ Medical Treatmet and 46 NEC III/Surgical intervention cases | n=68 suspected NEC II or NEC III receving medical treatment only | NEC II vs NEC III | Post-diagnostic (d=0.5) | 1.09 (g/L) | 0.715 | 50 | 85.29 |
| **Fibrinogen** | Zhang et al. (2024) | Retrospective Cohort | n=249 Preterm Neonates with 22 NEC I, 91 NEC II, and 136 NEC III cases, BW<2000g, GA<36weeks | n= 113 Preterm Neonates with NEC I/II | NEC II vs NEC III | Diagnostic | ≥4.0 g/L | 0.797 | 0.74 | 0.7 |
| **Fibrinogen Peptides (FGA)** | Sylvester et al. (2014) | Multicentre Prospective | n=119 Preterm Neoantes with 85 NEC cases (inlclduding NEC II (59) NEC III (26)), 17 Sepsis cases, and 17 controls | n=17 Preterm Healthy controls with matched GA and BW | NEC II/III vs Controls | Diagnostic | NA | 0.684 | 70 | 52 |
|  |  |  |  | n=59 Preterm infants with NEC II | NEC II vs NECIII |  |  | 0.744 | 70 | 73 |
| **Coagulant Factor XIII** | Guo-Zhong et al. (2015) | Case Control | n=84 Preterm and Term Neonates with 43 NEC II ,41 NEC III cases, and 24 controls | n= 24 Preterm neonates without NEC, matached for GA and BW | NEC II/III vs Controls | Pre-diagnostic | NA | 0.91 | NA | NA |
|  |  |  |  |  |  |  |  |  |  |  |
|  |  |  |  |  |  |  |  |  |  |  |
|  |  |  |  |  |  |  |  |  |  |  |
| **TRAIL** | Dong et al. (2023) | Prospective Case Contol | n=88, Preterm Neonates with 30 NEC cases (including NEC II (18) and NEC III (12), 29 Sepsis, and 29 controls, BW<2500g, GA<37weeks | n=29 Premterm Healthy neonates without NEC or other infectious disease | NEC II/III vs Controls | Diagnostic | 5.279 | 0.793 (0.666–0.920) | 96.6 | 66.7 |
| **TSLP** | Dong et al. (2023) | Prospective Case Contol | n=88, Preterm Neonates with 30 NEC cases (including NEC II (18) and NEC III (12), 29 Sepsis, and 29 controls, BW<2500g, GA<37weeks | n=29 Premterm Healthy neonates without NEC or other infectious disease | NEC II/III vs Controls | Diagnostic | − 0.177 | 0.814 (0.702–0.926) | 96.6 | 63.3 |
| **MCP-4** | Dong et al. (2023) | Prospective Case Contol | n=88, Preterm Neonates with 30 NEC cases (including NEC II (18) and NEC III (12), 29 Sepsis, and 29 controls, BW<2500g, GA<37weeks | n=29 Premterm Healthy neonates without NEC or other infectious disease | NEC II/III vs Controls | Diagnostic | 15.661 | 0.737 (0.608–0.865) | 51.7 | 90 |
|  |  |  |  |  |  |  |  |  |  |  |
|  |  |  |  |  |  |  |  |  |  |  |
|  |  |  |  |  |  |  |  |  |  |  |
|  |  |  |  | n=30 Pretem Neonates with NEC II (18) or NEC III (10) | NEC II vs NEC III |  | 15.5 | 0.690 (0.512–0.868) | 92.3 | 35.3 |
|  |  |  |  |  |  |  |  |  |  |  |
|  |  |  |  |  |  |  |  |  |  |  |
| **TNFSF14** | Dong et al. (2023) | Prospective Case Contol | n=88, Preterm Neonates with 30 NEC cases (including NEC II (18) and NEC III (12), 29 Sepsis, and 29 controls, BW<2500g, GA<37weeks | n=29 Premterm Healthy neonates without NEC or other infectious disease | NEC II/III vs Controls | Diagnostic | 5.024 | 0.675 (0.531–0.818) | 72.4 | 70 |
| **LIF** | Dong et al. (2023) | Prospective Case Contol | n=88, Preterm Neonates with 30 NEC cases (including NEC II (18) and NEC III (12), 29 Sepsis, and 29 controls, BW<2500g, GA<37weeks | n=30 Pretem Neonates with NEC II (18) or NEC III (10) | NEC II vs NEC III | Diagnostic | 0.5 | 0.674 (0.487–0.862) | 38.5 | 82.4 |
|  |  |  |  |  |  |  |  |  |  |  |
|  |  |  |  |  |  |  |  |  |  |  |
| **LIF** |  |  |  | n=29 Premterm Healthy neonates without NEC or other infectious disease | NEC II/III vs Controls |  | − 1.141 | 0.846 (0.748–0.944) | 79.3 | 76.7 |
|  |  |  |  |  |  |  |  |  |  |  |
|  |  |  |  |  |  |  |  |  |  |  |
| **CCL20** | Dong et al. (2023) | Prospective Case Contol | n=88, Preterm Neonates with 30 NEC cases (including NEC II (18) and NEC III (12), 29 Sepsis, and 29 controls, BW<2500g, GA<37weeks | n=30 Pretem Neonates with NEC II (18) or NEC III (10) |  |  |  |  |  |  |
|  |  |  |  |  | NEC II vs NEC III | Diagnostic | 11.5 | 0.887 (0.775–0.999) | 61.5 | 100 |
|  |  |  |  |  |  |  |  |  |  |  |
|  |  |  |  | n=29 Premterm Healthy neonates without NEC or other infectious disease | NEC II/III vs Controls |  | 9.994 | 0.851 (0.745–0.956) | 96.6 | 66.7 |
|  |  |  |  |  |  |  |  |  |  |  |
|  |  |  |  |  |  |  |  |  |  |  |
| **OPG** | Dong et al. (2023) | Prospective Case Contol | n=88, Preterm Neonates with 30 NEC cases (including NEC II (18) and NEC III (12), 29 Sepsis, and 29 controls, BW<2500g, GA<37weeks | n=30 Pretem Neonates with NEC II (18) or NEC III (10) | NEC II vs NEC III | Diagnostic | 9.365 | 0.851 (0.712–0.990) | 61.5 | 94.1 |
| **IL-6** | Wang et al. (2023) | Retrospective Cohort | n=150 Preterm and Term infants with 58 NEC II and 92 NEC III cases | n=58 Preterm infants with NEC II | NEC II vs NECIII | Diagnostic | NA | 0.635 | 60.3 | 68.35 |
|  | Wisgril et al. (10) | Retrospective | n=40, neonates with 24 NEC cases (6 NECI, 6 NEC II, 12 NEC III, and 16 culture proven LOS as controls | n=6 Infants with NEC II | NEC Stage III vs NEC Stage II | Diagnostic and 6 hr prior to surgery | 1400 pg/ml | 0.931 (0.83-1.0) | 91.7 | 83.3 |
|  |  |  |  |  |  |  |  |  |  |  |
|  |  |  |  |  |  |  |  |  |  |  |
|  | Cakir et al. (2018) | Prospective Case Control | n=84 Preterm infants, BW<1500g, GA<32weeks | n=42, Preterm infants matched for GA and weight | NEC II/III vs Controls | Diagnostic | >107pg/ml | 0.667 (0.449-0.884) | 100 | 73.4 |
|  |  |  |  |  |  | 3rd day of diagnosis (d=3) |  |  |  |  |
|  |  |  |  |  |  | Post-diagnostic (d=3) | >250.5pg/ml | 1.000 (1.000-1.000) | 100 | 83.3 |
|  |  |  |  |  |  | Post-diagnostic (d=7) | >222.50pg/ml | 1.000  (1.000-1.000) | 77.8 | 90.6 |
|  | Yakut et al. (11) | Prospective Case Control | n= 73 Preterm infants with 37 NEC cases and 36 controls, GA<32 weeks, BW<1500g | n=20 Preterm NEC II cases | NEC II vs NECIII | Diagnostic | 73.2 (pg/ml) | 0.593 | 63.2 | 56 |
|  |  |  |  |  |  |  |  |  |  |  |
|  |  |  |  |  |  | Post-diagnosis (d=3) | 136.5 (pg/ml) | 0.839 | 84.2 | 80 |
|  |  |  |  |  |  |  |  |  |  |  |
|  |  |  |  |  |  | Post-diagnosis (d=7) | 231 (pg/ml) | 0.662 | 78.9 | 52 |
|  |  |  |  |  |  |  |  |  |  |  |
| **IL-8** | Dong et al. (2023) | Prospective Case Contol | n=88, Preterm Neonates with 30 NEC cases (including NEC II (18) and NEC III (12), 29 Sepsis, and 29 controls, BW<2500g, GA<37weeks | n=30 Pretem Neonates with NEC II (18) or NEC III (10) | NEC II vs NEC III | Diagnostic | 9.334 | 0.778 (0.601–0.955) | 61.5 | 88.2 |
|  |  |  |  | n=29 Premterm Healthy neonates without NEC or other infectious disease | NEC II/III vs Controls |  | 6.87 | 0.907 (0.813–1.0) | 96.6 | 86.7 |
|  | Benkoe Mechtler et al. (2014) | Prospective | n=29, BW<2000g | n=14, GA, BW, and age at diagnosis matached infants | NEC II/III vs Controls | Diagnostic | NA | 0.99 (0.97-1.00) | NA | NA |
|  | Benkoe, Reck, et al. (12) | Retrospective | n=113, Preterm infants diagnosed with NEC (and had undergone medical/surgical treatment) | n=63 infants with Bell Stage II NEC | NEC III vs NEC II | Diagnostic and 6 hr prior to surgery | >1783pg/ml | 0.82 (0.74-0.90) | 90.5 | 59.2 |
| **IL-24** | Dong et al. (2023) | Prospective Case Contol | n=88, Preterm Neonates with 30 NEC cases (including NEC II (18) and NEC III (12), 29 Sepsis, and 29 controls, BW<2500g, GA<37weeks | n=29 Premterm Healthy neonates without NEC or other infectious disease | NEC II/III vs Controls | Diagnostic | − 0.147 | 0.844 (0.737–0.950) | 89.7 | 73.3 |
|  |  |  |  | n=30 Pretem Neonates with NEC II (18) or NEC III (10) | NEC II vs NEC III |  | 1.5 | 0.735 (0.552–0.918) | 61.5 | 76.5 |
| **IL-33** | Cakir et al. (2018) | Prospective Case Control | n=84 Preterm infants, BW<1500g, GA<32weeks | n=42, Preterm infants matched for GA and weight | NEC II/III vs Controls | Diagnostic | >3.1ng/ml | 0.838  (0.658-1.000) | 100 | 55.6 |
|  |  |  |  |  |  | Post-diagnostic (d=3) | >3.59ng/ml | 0.991  (0.000-1.000) | 100 | 94.4 |
|  |  |  |  |  |  | Post-diagnostic (d=7) | >4.01ng/ml | 1.000  (1.000-1.000) | 100 | 94.4 |
| **TGF-beta 1** | ER Abd Al Monaem et al. (13) | Prospective Case Contol | n=102,  Preterm Neonates with 52 NEC cases and 50 controls,  BW<1500g, GA<32weeks | n=50 preterm infants, matched for GA and BW, without NEC | NEC II vs controls | Diagnostic | ≤996.3 pg/ml | 0.738 (0.59–0.89) | 80 | 65 |
|  |  |  |  |  |  | Post-diagnostic (d=7) | ≥1019.7 pg/ml | 0.899 (0.77–1.0) | 100 | 80 |
| **DSNLT** | Masi et al. (2020) | Prospective Cohort | n=77, Preterm infants, GA<32weeks | n=37, Preterm healthy ifnants matched by BW and GA | NEC >II vs Controls | Pre-diagnostic | 241 nmol/mL | 0.947 (0.88-0.981) | 90 | 90 |
| **RELMb** | Luo et al. (1) | Case Control | n=58, Preterm infants | n=29, preterm infants matached for GA and GW | NEC >II vs Controls | Diagnostic | 378.3 ng/L | 0.739 | 71.4 | 91.7 |
|  | Xiao Chen Leu et al. (2022) | Prospective Cohort | n=49, Preterm and Term Neonates with 26 NEC II and 23 NEC III cases | n=26 Preterm infants with NEC II | NEC II vs NEC III | Diagnostic | 19.7 μmol/L | 0.723 (0.582–0.865) | 56.5 | 80.8 |
| **CD14** | Sylvester et al. (2014) | Multicentre Prospective | n=119 Preterm Neoantes with 85 NEC cases (inlclduding NEC II (59) NEC III (26)), 17 Sepsis cases, and 17 controls | n=59 Preterm infants with NEC II | NEC II vs NECIII | Diagnostic | NA | 0.775 | 80 | 60 |
| **CD14** |  |  |  | n=17 Preterm Healthy controls with matched GA and BW | NEC II/III vs Controls |  |  | 0.651 | 60 | 64 |
| **CCL16** | Mackay et al. (14) | Prospective | n=18 Preterm and Term infants with 12 NEC cases and 6 matched controls | n=6 Preterm and Term infants wihtout NEC | NEC II/III vs Controls | Diagnostic | NA | 0.744 (0.535–0.953) | NA | NA |
| **CXCL** | Dong et al. (2023) | Prospective Case Contol | n=88, Preterm Neonates with 30 NEC cases (including NEC II (18) and NEC III (12), 29 Sepsis, and 29 controls, BW<2500g, GA<37weeks | n=29 Premterm Healthy neonates without NEC or other infectious disease | NEC II/III vs Controls | Diagnostic | 11.023 | 0.787 (0.670–0.905) | 93.1 | 56.7 |
| **CXCL6** | Mackay et al. (14) | Prospective | n=18 Preterm and Term infants with 12 NEC cases and 6 matched controls | n=6 Preterm and Term infants wihtout NEC | NEC II/III vs Controls | Diagnostic | NA | 0.802 (0.587–0.966) | NA | NA |
| **COLEC12** | Mackay et al. (14) | Prospective | n=18 Preterm and Term infants with 12 NEC cases and 6 matched controls | n=6 Preterm and Term infants wihtout NEC | NEC II/III vs Controls | Diagnostic | NA | 0.826 (0.650–1.00) | NA | NA |
| **MICA** | Mackay et al. (14) | Prospective | n=18 Preterm and Term infants with 12 NEC cases and 6 matched controls | n=6 Preterm and Term infants wihtout NEC | NEC II/III vs Controls | Diagnostic | NA | 0.802 (0.611–0.992) | NA | NA |
| **CRP** | Guo et al. (2024) | Retrospective | n=90, Preterm Infants with 52 NEC II cases and 38 NEC III cases, GA<37weeks | n=52 Preterm infants diagnosied with NEC II | NEC II vs NEC III | Post-diagnosis (d=1) | 14.65 mg/l | 0.67 (0.55–0.78) | 79 | 67 |
|  | Liu et al. (2024) | Prospective Cohort | n=70 Preterm Neonates with 18 NEC II cases, 12 NEC III, and 40 Controls,BW<1500g,GA<32weeks, | n=40, Preterm Healthy infants without NEC | NEC II/III vs Controls | Pre-diagnostic | 0.575 mg/dL | 0.656 (0.529–0.784) | 63.3 | 52.5 |
|  | Meng et al. (4) | Retrospective Cohort | n=122 Preterm Neonates with 79 NEC I/II cases and 43 NEC III cases | n=79 Preterm infants with NEC I/II | NEC II vs NEC III | Pre-diagnostic | 7.635 (mg/L) | 0.919 | 88.6 | 86 |
|  | Wang et al. (2023) | Retrospective Cohort | n=150 Preterm and Term infants with 58 NEC II and 92 NEC III cases | n=58 Preterm infants with NEC II | NEC II vs NECIII | Diagnostic | NA | 0.805 | 75.9 | 80.4 |
|  | Shen et al. (2024) | Prospective | n=108 Preterm Neonates with 42 NEC cases (inlcuding NEC (24) and NEC III (18) and 66 controls, BW<1500g, GA<30weeks | n=66 Preterms matached for GA and BW | NEC II/III vs controls | Diagnostic | >6.25 mg/l | 0.69 | 76.2 | 57.6 |
|  | Dong et al. (2023) | Prospective Case Contol | n=88, Preterm Neonates with 30 NEC cases (including NEC II (18) and NEC III (12), 29 Sepsis, and 29 controls, BW<2500g, GA<37weeks | n=29 Premterm Healthy neonates without NEC or other infectious disease | NEC II/III vs Controls | Diagnostic | 12.53mg/L | 0.917 (0.839–0.994) | 100 | 80 |
|  | Zhang et al. (2024) | Retrospective Cohort | n=249 Preterm Neonates with 22 NEC I, 91 NEC II, and 136 NEC III cases, BW<2000g, GA<36weeks | n= 113 Preterm Neonates with NEC I/II | NEC II vs NEC III | Diagnostic | ≥10 mg/L | 0.771 | 0.8 | 0.5 |
| **CRP** | P.C. Ng et al. (15) | Prospective Cohort | n=152, Preterm infants, GA<34weeks | n=128, Preterm infants without NEC (GA matched within 2 weeks) | NEC II/III vs Controls | Diagnostic | 10mg/L | 0.694 (0.567-0.821) | 63 | 70 |
|  |  |  |  |  |  | Post-diagnosis (d=1) | 10mg/L | 0.789  (0.675-0.896) | 79 | 65 |
|  | Coufal et al. (2020) | Retrospective | n=37, Preterm and Term infants admitted to Paediatric Surgery Department | N=11, Infants with NEC Bell Stage I | NEC Stage III vs NEC Stage II | Diagnostic or Post-surgery | NA | 0.779 | NA | NA |
|  | Ng et al. (16) | Prospective Cohort | n=301 Preterm infants including NEC, Septicimea, Non-NEC, and Healthy infantss | Preterm Non-NEC and Healthy infants | NEC II/III vs Controls | Post-diagnosis (d=1) | 10mg/L | NA | 92 | 55 |
|  | Cakir et al. (2018) | Prospective Case Control | n=84 Preterm infants, BW<1500g, GA<32weeks | n=42, Preterm infants matched for GA and weight | NEC II/III vs Controls | Diagnostic | >15.7mg/L | 0.250  (0.000-1.000) | 66.7 | 33.3 |
|  |  |  |  |  |  | Post-diagnosis (d=3) | >31.5mg/L | 0.486  (0.203-0.769) | 64.3 | 35.7 |
|  |  |  |  |  |  | Post-diagnostic (d=7) | >69.5mg/L | 1.000  (1.000-1.000) | 57.1 | 62.9 |
|  | Yang et al. (9) | Cross-sectional | n=161 Preterm Neonates with 41 NEC I, 34 NEC II, 28 NEC III cases, and 58 controls, GA<34 weeks | n=34 Preterm Neonates with NEC II | NEC II vs NEC III | Post-diagnostic (d=1) | NA | 0.825 (0.712-0.938) | 89.5 | 65 |
|  |  |  |  | n=58 Preterm Neonates without NEC | NEC II/III vs Controls | Post-diagnostic (d=1) | NA | 0.65 (0.538-0.762) | 93 | 31.7 |
|  | Shah et al. (2017) | Prospective Case Control | n=53, 14 Infants with NEC (including NEC II (10) and NEC III (4)), 13 Infants with SIP, and 26 infants as matched control | n=39, Preterm infants with SIP (13) and matched controls (26) | NEC II/III vs Controls | Diagnostic | <4mg/L | 0.65 (0.54-0.90) | 100 | 64.7 |
|  | Reisinger et al. (3) | Cohort | n=29, Preterm infants with 13 NEC II and 16 NEC III cases, BW<1500g, GA<32 weeks | n=13 Preterm infants with diaognosed NEC II | NEC II vs NEC III | Diagnostic | >27.8 ng/ml | 0.78 (0.61–0.95) | 71 | 83 |
|  |  |  |  |  |  | Diagnostic or Post-surgery | >34.4 ng/ml | 0.87 (0.73–1.00) | 83 | 83 |
|  | Yakut et al. (11) | Prospective Case Control | n= 73 Preterm infants with 37 NEC cases and 36 controls, GA<32 weeks, BW<1500g | n=20 Preterm NEC II cases | NEC II vs NECIII | Diagnostic | 16.05 (mg/l) | 0.346 | 57.9 | 33 |
|  |  |  |  |  |  | Post-diagnostic (d=3) | 33.75 (mg/l) | 0.641 | 68.4 | 48 |
|  |  |  |  |  |  | Post-diagnostic (d=7) | 61.55 (mg/l) | 0.713 | 68.4 | 76 |
| **Procalcitonin (PCT)** | Wang et al. (2023) | Retrospective Cohort | n=150 Preterm and Term infants with 58 NEC II and 92 NEC III cases | n=58 Preterm infants with NEC II | NEC II vs NECIII | Diagnostic | NA | 0.844 | 86.2 | 79.3 |
|  | Meng et al. (4) | Retrospective Cohort | n=122 Preterm Neonates with 79 NEC I/II cases and 43 NEC III cases | n=79 Preterm infants with NEC I/II | NEC II vs NEC III | Pre-diagnostic | 7.635 (ng/mL) | 0.919 | 88.6 | 86 |
| **IaIp** | Shah et al. (2017) | Prospective Case Control | n=53, 14 Infants with NEC (including NEC II (10) and NEC III (4)), 13 Infants with SIP, and 26 infants as matched control | n=39, Preterm infants with SIP (13) and matched controls (26) | NEC II/III vs Controls | Diagnostic | <207mg/L | 0.98 (0.84-0.99) | 100 | 88.2 |
| **SAA** | Wang et al. (2023) | Retrospective Cohort | n=150 Preterm and Term infants with 58 NEC II and 92 NEC III cases | n=58 Preterm infants with NEC II | NEC II vs NECIII | Diagnostic | NA | 0.864 | 84.5 | 80.55 |
|  | Coufal et al. (2020) | Retrospective | n=37, Preterm and Term infants admitted to Paediatric Surgery Department | N=11, Infants with NEC Bell Stage I | NEC Stage III vs NEC Stage II | Diagnostic or Post-surgery | NA | 0.779 | NA | NA |
| **SAA** | Reisinger et al. (3) | Cohort | n=29, Preterm infants with 13 NEC II and 16 NEC III cases, BW<1500g, GA<32 weeks | n=13 Preterm infants with diaognosed NEC II | NEC II vs NEC III | Diagnostic | >27.8 ng/ml | 0.78 (0.61–0.95) | 71 | 83 |
|  |  |  |  |  |  | Diagnostic followed by surgery | >34.4 ng/ml | 0.87 (0.73–1.00) | 83 | 83 |
| **Fecal Calprotectin** | Liu et al. (2024) | Prospective Cohort | n=70 Preterm Neonates with 18 NEC II cases, 12 NEC III, and 40 Controls,BW<1500g,GA<32weeks, | n=40, Preterm Healthy infants without NEC | NEC II/III vs Controls | Pre-diagnostic | 428.99 ug/g | 0.787 (0.679–0.895) | 76.7 | 67.5 |
| **Calprotectin (S100A8/A9)** | Bareketain et al. (17) | Case control | n=70, Preterm infants, BW<1500g | n=35, infants who didnot develop NEC | NEC II/III vs Controls | Diagnostic | >176 µg/g | 0.999 | 97.14 | 100 |
|  | P.C. Ng et al. (15) | Prospective Cohort | n=152, Preterm infants, GA<34weeks | n=130, Preterm infants without NEC (GA matched within 2 weeks) | NEC Stage >II and Sepsis vs Controls | Diagnostic | 308 µg/g feces | 0.726  (0.638-0.814) | 75 | 62 |
|  |  |  |  |  |  |  |  |  |  |  |
|  |  |  |  |  |  |  |  |  |  |  |
|  |  |  |  |  |  |  |  |  |  |  |
|  | Thiubalt et al. (2021) | Multicentre Prospective | 133VLBW infants with 8 NEC and Non-NEC cases | n= 125 preterm neonates with mean GA<32 weeks and BW<1500g | NEC III vs Controls | Pre-diagnostic (d=10) | 6.2 µg/g feces | 0.81 | 74 | 74 |
|  | Terrin et al. (18) | Multicentre Prospective | n=72 neonates, GA<32weeks | n=41 with stable profiles | NEC II/III vs Controls | Post-diagnostic (d=3) | 3.0 mg/ml | 0.996 | 100 | 96.4 |
| **FOBT** | Pickering et al. (19) | Retrospective Cohort | n=273 preterm infants with 8 NEC II/III cases and 195 controls, | n=195 preterm infants without NEC | NEC II/III vs Controls | Post-diagnostic (d=2) | NA | NA | 0 | 34.4 |
| **TFF-3** | E.W.Y. Ng et al. (2013) | Case Control | n=100, Preterm infants, BW<1500g, GA<30weeks | n=80, with Septicimea (40) and Non-Sepsis/NEC (40) controls | NEC II/III vs Controls | Diagnostic | >12.5 ng/mL | 0.83 (0.73-0.94) | 50 | 98 |
|  |  |  |  | n=40 with Non-Sepsis/non-NEC (40) controls | NEC Stage >II vs Controls |  | >12.5 ng/mL | 0.79 (0.67-0.92) | NR | NR |
|  |  |  |  | n=8, Infants with NEC II | NEC III vs NEC II |  | >12.5 ng/mL | NA | 67 | 75 |
|  |  |  |  | n=80, with Septicimea (40) and Non-Sepsis/NEC (40) controls | NEC III vs Septicimea/Controls |  | >12.5 ng/mL | NA | 67 | 98 |
|  |  |  |  |  |  |  |  |  |  |  |
|  |  |  |  |  |  |  |  |  |  |  |
|  |  |  |  |  |  |  |  |  |  |  |
| **CBG** | Gómez-Chaparro Moreno et al. (20) | Prospective Case Control | n=205, Premature Infants, BW<1500g, GA<35weeks | n=128 premature infants with other patholgies except NEC | NEC II/III vs Controls | Diagnostic | 15.6 mU/mg | 0.89 | 84.6 | 85.9 |
| **Gal-4** | Fundora et al. (2021) | Prospective Cohort | n=167, Preterm of Full-term infants | n=28, matched controls | NEC Stage III vs Controls | Post-diagnostic (d=1 or 2) | >0.7 ng/ml | 0.84 | 71 | 89 |
| **Gal-4** | Fundora et al. (2021) | Prospective Cohort | n=167, Preterm of Full-term infants | n=28, matched controls | NEC Stage III vs Controls | Post-diagnostic (d=1 or 2) | 1.38 ng/ml | 0.84 | 64 | 96 |
| **FABP** | Gregory et al. (21) | Case Control | n=70 Preterm Neonates with 18 NEC I cases, 21 NEC II, and 31 NEC III Cases, GA<29 weeks | n=70 Preterm Neonates with 18 NEC I cases, 21 NEC II, and 31 NEC III Cases, GA<29 weeks | NEC II/III vs Controls | Pre-diagnostic (d=7) | >13.3 ng/mL | 0.85 | 60 | 78 |
|  |  |  |  |  |  | Pre-diagnostic (d=3) | >13.9 ng/mL | 0.9 | 65 | 84 |
| **L-FABP** | Benkoe Mechtler et al. (2014) | Prospective | n=29, BW<2000g | n=14, GA, BW, and age at diagnosis matached infants | NEC II/III vs Controls | Diagnostic | NA | 0.95 (0.87-1.00) | NA | NA |
|  | E.W.Y. Ng et al. (2013) | Case Control | n=100, Preterm infants, BW<1500g, GA<30weeks | n=80, with Septicimea (40) and Non-Sepsis/NEC (40) controls | NEC Stage >II vs Septicimea/Controls | Diagnostic | >296 ng/ml | 0.79 (0.66-0.91) | 50 | 96 |
|  |  |  |  | n=40 with Septicimea (40) cases | NEC Stage >II vs Septicimea |  | >296 ng/ml | 0.74 (0.60-0.89) | NA | NA |
|  |  |  |  | n=40 with Non-Sepsis/non-NEC (40) controls | NEC II/III vs Controls |  | >296 ng/ml | 0.84 (0.72-0.95) | NA | NA |
|  |  |  |  | n=8, Infants with NEC II | NEC III vs NEC II |  | >296 ng/ml | NA | 75 | 88 |
|  |  |  |  | n=80, with Septicimea (40) and Non-Sepsis/NEC (40) controls | NEC III vs Septicimea/Controls |  | >296 ng/ml | NA | 75 | 96 |
| **I-FABP** | Liu et al. (2024) | Prospective Cohort | n=70 Preterm Neonates with 18 NEC II cases, 12 NEC III, and 40 Controls,BW<1500g,GA<32weeks, | n=40, Preterm Healthy infants without NEC | NEC II/III vs Controls | Pre-diagnostic | 2.54 ng/mL | 0.897 (0.825–0.968) | 76.7 | 87.5 |
|  | Wang et al. (2023) | Retrospective Cohort | n=150 Preterm and Term infants with 58 NEC II and 92 NEC III cases | n=58 Preterm infants with NEC II | NEC II vs NECIII | Diagnostic | NA | 0.872 | 82.8 | 80.4 |
|  | Benkoe Mechtler et al. (2014) | Prospective | n=29, BW<2000g | n=14, GA, BW, and age at diagnosis matached infants | NEC II/III vs Controls | Diagnostic | NA | 0.81 (0.66-0.97) | NA | NA |
|  | El Abd Ahmed et al. (19) | Prospective Cohort | n=78, Preterm or Fullterm infants, GA<37weeks | n=23 heathy infants matached for GA and sex | NEC II/III vs Controls | Diagnostic | >6.95 nl/ml | 0.768 | 75 | 100 |
|  |  |  |  |  |  |  |  |  |  |  |
|  |  |  |  |  |  |  |  |  |  |  |
|  |  |  |  |  |  |  |  |  |  |  |
|  |  |  |  | n=10 premature infants with NEC IIa | NEC Stage IIb vs NEC Stage IIa |  | >3.24 ng/ml | 0.881 | 90 | 72 |
|  |  |  |  |  |  |  |  |  |  |  |
|  |  |  |  |  |  |  |  |  |  |  |
|  |  |  |  |  |  |  |  |  |  |  |
| **I-FABP** | E.W.Y. Ng et al. (2013) | Case Control | n=100, Preterm infants, BW<1500g, GA<30weeks | n=80, with Septicimea (40) and Non-Sepsis/NEC (40) controls | NEC Stage >II vs Septicimea/Controls | Diagnostic | >7.7 ng/ml | 0.68 (0.52-0.85) | 50 | 95 |
|  |  |  |  | n=40 with Non-Sepsis/non-NEC (40) controls | NEC Stage >II vs Controls |  | >7.7 ng/ml | 0.68 (0.51-0.85) | NA | NA |
|  |  |  |  | n=8, Infants with NEC II | NEC III vs NEC II |  | >7.7 ng/ml | NA | 75 | 88 |
|  |  |  |  | n=80, with Septicimea (40) and Non-Sepsis/NEC (40) controls | NEC III vs Septicimea/Controls |  | >7.7 ng/ml | NA | 75 | 95 |
| **I-FABP** | Shaaban et al. (22) | Case Control | n=80 Preterm Neonates with 20 NEC !, 12 NEC II, 8 NEC III cases, and 40 controls GA<29weeks, BW<1700g | n=40, Preterm Healthy infants without NEC matched for GA and BW | NEC II/III vs Controls | Diagnostic | NA | 0.92 | NA | NA |
|  |  |  |  |  |  | Post-diagnositc | NA | 0.81 | NA | NA |
| **I-FABPu** | El Abd Ahmed et al. (23) | Prospective Cohort | n=78, Preterm or Fullterm infants, GA<37weeks | n=24 heathy infants matached for GA and sex | NEC II/III vs Controls | Diagnostic | >4.13 ng/g | 0.864 | 100 | 76.19 |
|  |  |  |  |  |  |  |  |  |  |  |
|  |  |  |  |  |  |  |  |  |  |  |
|  |  |  |  |  |  |  |  |  |  |  |
|  |  |  |  | n=11 Infants with NEC IIIa | NEC Stage IIb vs NEC IIIa |  | >2.93 ng/g | 0.821 | 90 | 92 |
|  |  |  |  |  |  |  |  |  |  |  |
|  |  |  |  |  |  |  |  |  |  |  |
|  |  |  |  |  |  |  |  |  |  |  |
| **HBD2** | Xaio Chen et al. (2022) | Prospective | n=60, Preterm and Term neonates with 33 NEC II and 27 NEC III cases | n=33 Preterm and Term neonates with NEC II | NEC II vs NEC III | Diagnostic | ≥ 1649.02 ng/g | 0.754 | 80 | 80 |
| **HBD2** | Liu et al. (2024) | Prospective Cohort | n=70 Preterm Neonates with 18 NEC II cases, 12 NEC III, and 40 Controls,BW<1500g,GA<32weeks, | n=40, Preterm Healthy infants without NEC | NEC II/III vs Controls | Pre-diagnostic | 339.60 ng/g | 0.633 (0.474–0.790) | 63.3 | 50 |
| **UDCA** | Gao et al. (2024) | Cohort | n=160 Preterm Neonates with 32 NEC cases and 128 controls matached for GA<37weeks | n=128 Healthy Preterm infants without NEC | NEC II/III vs Controls | Diagnostic | NA | 0.68 (0.57–0.79) | NA | NA |
| **GCDCA** |  |  |  |  |  |  | NA | 0.68 (0.57–0.80) | NA | NA |
| **GCA** |  |  |  |  |  |  | NA | 0.69 (0.57–0.81) | NA | NA |
| **Secondary BAs** |  |  |  |  |  |  | NA | 0.66 (0.56–0.75) | NA | NA |
| **TCA** |  |  |  |  |  |  | NA | 0.72 (0.61–0.83) | NA | NA |
| **TCDCA** |  |  |  |  |  |  | NA | 0.76 (0.65–0.86) | NA | NA |
| **DCA** |  |  |  |  |  |  | NA | 0.86 (0.80–0.93) | NA | NA |
| **Primary/Secondary BA Ratio** |  |  |  |  |  |  | 62.9 | 0.90 (0.84–0.97) | 94.5 | 78.1 |
|  |  |  |  | n=26 Preterm infants with NEC II | NEC II vs NEC III |  | 28.7 | 0.91 (0.80-1.00) | 100 | 76.9 |
| **Claudin-3** | Xaio Chen et al. (2022) | Prospective | n=60, Preterm and Term neonates with 33 NEC II and 27 NEC III cases | n=33 Preterm and Term neonates with NEC II | NEC II vs NEC III | Diagnostic | ≥ 2488.71 pg/g | 0.755 | 80 | 80 |
| **Citruline** | Jawale et al. (24) | Prospective Case Control | n=27, Preterm Infants, GA<32weeks | n=18, Preterm infants with NEC I like presentation (9) and healthy (9) GA matched infants | NEC II/III vs Controls | Diagnostic | 15 µmol/L | 0.88 | 78 | 89 |
|  | Celik et al. (25) | Prospective | n=36 Preterm neoantes with 20 NEC II/III cases and 16 controls, BW<1500g, GA<32 weeks | n=16 Preterm Healthy Neonates without NEC matched for GA and BW | NEC II/III vs Controls | Diagnostic | 13.15 μmol/l | 0.88 (0.77–0.99) | 80 | 82 |
| **Arginine** | Celik et al. (25) | Prospective | n=36 Preterm neoantes with 20 NEC II/III cases and 16 controls, BW<1500g, GA<32 weeks | n=16 Preterm Healthy Neonates without NEC matched for GA and BW | NEC II/III vs Controls | Diagnostic | 28.52 μmol/l | 0.79 (0.64–0.94) | 70 | 75 |
| **IAP** | Heath et al. (2019) | Multicentre Prospective | n=136 Preterm infants with 25 NEC cases and 42 controls, GA<30.9weeks BW<1350g | n=111, preterm infants with suspected NEC I/II (19) and Non-Nec (92) | NEC III vs Controls | Diagnostic | NA | 0.97 (0.93-1.00) | NA | NA |
|  |  |  |  |  |  |  |  |  |  |  |
|  |  |  |  |  | NEC III vs Controls |  | NA | 0.76 (0.64-0.86) | NA | NA |
|  |  |  |  |  |  |  |  |  |  |  |
| **IAP Content** |  |  |  |  | NEC II vs controls |  | NA | 0.97 (0.93-1.00) | NA |  |
|  |  |  |  |  |  |  | NA | 0.97 (0.93-1.00) | NA | NA |
| **IAP Activity** |  |  |  |  | NEC II vs controls |  | NA | 0.62 (0.48-0.77) | NA | NA |
|  |  |  |  |  |  |  | NA | 0.76 (0.64-0.86) | NA | NA |
| **Acetic Acid (SCFA)** | Xiao Chen Liu et al. (2022) | Prospective Cohort | n=34 Preterm infants with 17 NEC cases and 17 controls, GA<34 weeks | n=17 Preterm Healthy controls with matched GA and BW | NEC II/III vs Controls | Pre-diagnostic | NA | 0.73 (0.55-0.90) | NA | NA |
| **Propionoic Acid (SCFA)** |  |  |  |  |  |  | NA | 0.7 (0.52-0.88) | NA | NA |
| **Butyric Acid (SCFA)** |  |  |  |  |  |  | NA | 0.68 (0.50-0.86) | NA | NA |
| **Succinate (TCA metabolite)** | Ting Du et al. (26) | Prospective Cohort | n=32 Preterm infants with 16 NEC and 16 controls | n=16 Preterm infants without NEC | NEC II/III vs Controls | Prior to diagnosis | NA | 0.6641 (0.4645-0.8636) | NA | NA |
| **L-Malic Acid (TCA metabolite)** |  |  |  |  |  |  | NA | 0.7617 (0.5946-0.9289) | NA | NA |
| **Oxaloacetate (TCA metabolite)** |  |  |  |  |  |  | NA | 0.7344 (0.5538 -0.9149) | NA | NA |
| **IGHA1 IGHA2** | Mackay et al. (14) | Prospective | n=18 Preterm and Term infants with 12 NEC cases and 6 matched controls | n=6 Preterm and Term infants wihtout NEC | NEC II/III vs Controls | Diagnostic | NA | 0.826 (0.630–1.00) | NA | NA |
| **Haptoglobin** | Thiubalt et al. (2021) | Multicentre Prospective | n=134 VLBW infants with 8 NEC and Non-NEC cases | n= 126 preterm neonates with mean GA<32 weeks and BW<1500g | NEC III vs Controls | Pre-diagnostic (d=10) | 14301 µg/g feces | 0.7 | 78 | 62 |
| **Lipocalin-2** | Thiubalt et al. (2021) | Multicentre Prospective | n=134 VLBW infants with 8 NEC and Non-NEC cases | n= 126 preterm neonates with mean GA<32 weeks and BW<1500g | NEC III vs Controls | Pre-diagnostic (d=10) | 227 µg/g feces | 0.82 | 70 | 77 |
| **Calgranulin-C (S100A12)** | Däbritz et al. (2012) | Prospective Case Control | n=145 Preterm infants with NEC (18) or without NEC or anyother GI distress (127), BW<1500g | n=127 without any GI distress | NEC II/III vs Controls | Diagnostic | 210 µg/kg | 0.70 (0.54-0.86) | 67 | 78 |
|  |  |  |  |  | NEC II/III vs Controls | Pre-diagnostic (d=7) | 65 µg/kg | 0.64 (0.53-0.75) | 76 | 56 |
|  |  |  |  |  | NEC II vs Controls | Diagnostic | 468 µg/kg | 0.76 (0.59-0.93) | 71 | 87 |
|  |  |  |  |  | NEC II vs Controls | Pre-diagnostic (d=7) | 65 µg/kg | 0.77 (0.68-0.86) | 96 | 56 |
| **Lactic Acid  (LA)** | Shen et al. (2024) | Prospective | n=108 Preterm Neonates with 42 NEC cases (inlcuding NEC (24) and NEC III (18) and 66 controls, BW<1500g, GA<30weeks | n=66 Preterms matached for GA and BW | NEC II/III vs controls | Diagnostic | <1.05mmol/L | 0.813  (0.730-0.897) | 85.7 | 68.2 |
| **Endocan** | Cakir et al. (2018) | Prospective Case Control | n=84, Preterm infants, GA<32 weeks, BW<1500g | n=42 infants matached for GA and BW | NEC II/III vs Controls | Diagnostic | >1414.65 ng/ml | 1.000 (1.000-1.000) | 100 | 83.3 |
|  |  |  |  |  |  | Post-diagnostic (d=3) | 1600.10 ng/ml | 0.833 (0.655-1.000) | 100 | 85.6 |
|  |  |  |  |  |  | Post-diagnostic (d=7) | >206.08 ng/ml | 0.056 (0.000-1.000) | 50 | 11.1 |
| **RIPK3** | Shen et al. (2024) | Prospective | n=108 Preterm Neonates with 42 NEC cases (inlcuding NEC (24) and NEC III (18) and 66 controls, BW<1500g, GA<30weeks | n=66 Preterms matached for GA and BW | NEC II/III vs controls | Diagnostic | <20.52ng/mL | 0.864 (0.797-0.932) | 100 | 72.7 |
| **MMP10** | Dong et al. (2023) | Prospective Case Contol | n=88, Preterm Neonates with 30 NEC cases (including NEC II (18) and NEC III (12), 29 Sepsis, and 29 controls, BW<2500g, GA<37weeks | n=29 Premterm Healthy neonates without NEC or other infectious disease | NEC II/III vs Controls | Diagnostic | 9.24 | 0.799 (0.682–0.916) | 62.1 | 90 |
| **MMP13** | Mackay et al. (14) | Prospective | n=18 Preterm and Term infants with 12 NEC cases and 6 matched controls | n=6 Preterm and Term infants wihtout NEC | NEC II/III vs Controls | Diagnostic | NA | 0.777 (0.574–0.980) | NA | NA |
| **A2ML1** | Sylvester et al. (2014) | Multicentre Prospective | n=119 Preterm Neoantes with 85 NEC cases (inlclduding NEC II (59) NEC III (26)), 17 Sepsis cases, and 17 controls | n=17 Preterm Healthy controls with matched GA and BW | NEC II/III vs Controls | Diagnostic | NA | 0.849 | 80 | 76 |
|  |  |  |  | n=59 Preterm infants with NEC II | NEC II vs NECIII |  | NA | 0.804 | 70 | 80 |
| **CST3** |  |  |  | n=17 Preterm Healthy controls with matched GA and BW | NEC II/III vs Controls | Diagnostic | NA | 0.702 | 80 | 49 |
|  |  |  |  | n=59 Preterm infants with NEC II | NEC II vs NECIII |  | NA | 0.684 | 60 | 73 |
| **PEDF** |  |  |  | n=17 Preterm Healthy controls with matched GA and BW | NEC II/III vs Controls | Diagnostic | NA | 0.834 | 80 | 69 |
|  |  |  |  | n=59 Preterm infants with NEC II | NEC II vs NECIII |  | NA | 0.839 | 80 | 68 |
| **RET4** |  |  |  | n=17 Preterm Healthy controls with matched GA and BW | NEC II/III vs Controls | Diagnostic | NA | 0.655 | 70 | 47 |
|  |  |  |  | n=59 Preterm infants with NEC II | NEC II vs NECIII |  | NA | 0.81 | 70 | 81 |
| **VASN** |  |  |  | n=17 Preterm Healthy controls with matched GA and BW | NEC II/III vs Controls | Diagnostic | NA | 0.733 | 60 | 68 |
|  |  |  |  | n=59 Preterm infants with NEC II | NEC II vs NECIII |  | NA | 0.7 | 70 | 59 |
| **AFP** | Mackay et al. (14) | Prospective | n=18 Preterm and Term infants with 12 NEC cases and 6 matched controls | n=6 Preterm and Term infants wihtout NEC | NEC II/III vs Controls | Diagnostic | NA | 0.926 (0.813–1.00) | NA | NA |
| **miRNA1290** | Ng et al. (16) | Prospective Cohort | n=301 Preterm infants including NEC, Septicimea, Non-NEC, and Healthy infantss | Preterm Non-NEC and Healthy infants | NEC II/III vs Controls | Diagnostic | >220 copies/µL | 0.92 (0.866-0.968) | 83 | 92 |
| **miRNA1290** |  |  |  |  |  |  | > 650 copies/µL | NA | 42 | 98 |
| **miRNA1246** |  |  |  |  |  |  | >330 copies/µL | 0.84 (0.766-0.921) | 72 | 94 |
| **miRNA451a** | P.C. Ng et al. (15) | Prospective Cohort | n=152, Preterm infants, GA<34weeks | n=128, Preterm infants without NEC (GA matched within 2 weeks) | NEC Stage >II and Sepsis vs Controls | Diagnostic | 9.5 copies/µg feces | 0.680  (0.580-0.779) | 75 | 60 |
|  |  |  |  |  |  |  |  |  |  |  |
|  |  |  |  |  |  |  |  |  |  |  |
|  |  |  |  |  |  |  |  |  |  |  |
| **miRNA375** |  | Prospective Cohort | n=301 Preterm infants including NEC, Septicimea, Non-NEC, and Healthy infantss | Preterm Non-NEC and Healthy infants | NEC II/III vs Controls |  | >422 copies/µL | 0.87 (0.796-0.942) | 81 | 85 |
| **miRNA223** |  | Prospective Cohort | n=152, Preterm infants, GA<34weeks | n=128, Preterm infants without NEC (GA matched within 2 weeks) | NEC Stage >II and Sepsis vs Controls |  | 130 copies/µg feces | 0.663 (0.546-0.779) | 75 | 62 |
|  |  |  |  |  |  |  |  |  |  |  |
|  |  |  |  |  |  |  |  |  |  |  |
|  |  |  |  |  |  |  |  |  |  |  |
| **Urinary Caveolin-1** | Corebima et al. (2023) | Single Centre Cohort | n=34 Preterm and Term Neonates with 12 NEC II/III cases, 12 without NEC, and 10 Healthy controls | n=24 Preterm and Term Neonates without NEC or anyother disease | NEC II/III vs Controls | Post-diagnostic (d=3) | ≥ 17.81 ng/dl | NA | 87.5 | 75 |
| **Hept-2-enal** | Probert et al. (2020) | Multicentre Prospective | n=102, Preterm infants, GA<34weeks | n=70, matched for weight and age, without developing NEC | NEC Stage >II vs Controls | Diagnostic/Post-diagnostic | 37 | 0.76 (0.66-0.85) | 78 | 68 |
| **Pent-1-ene-3-one** |  |  |  |  |  |  | 36 | 0.76 (0.67-0.86) | 72 | 73 |
| **2-Ethylfuran** |  |  |  |  |  |  | 38 | 0.76  (0.65-0.86) | 71 | 73 |
| **Pentanal** |  |  |  |  |  |  | 36 | 0.75  (0.64-0.85) | 72 | 73 |
| **2-Pentylfuran** |  |  |  |  |  |  | 37 | 0.75  (0.65-0.85) | 73 | 71 |
| **Albumin** | Sharif et al. (27) | Single Centre Retrospective | n=152 Preterm and Term Infants with 132 NEC II cases and 19 NEC III cases, | n=152 Preterm and Term Infants with 132 NEC II cases and 19 NEC III cases, | NEC II vs NEC III | Diagnostic | ≤ 20 g/L | 0.587 (0.478-0.696) | 25 | 94.92 |
|  |  |  |  |  |  | Post-diagnostic (d=2) | ≤ 20 g/L | 0.656 (0.551-0.762) | 40.98 | 83.33 |
| **PA (Pre-albumin)** | Yang et al. (9) | Cross-sectional | n=161 Preterm Neonates with 41 NEC I, 34 NEC II, 28 NEC III cases, and 58 controls, GA<34 weeks | n=58 Preterm Neonates without NEC | NEC II/III vs Controls | Post-diagnostic (d=1) | NA | 0.743 (0.635-0.85) | 93 | 51.2 |
|  |  |  |  | n=34 Preterm Neonates with NEC II | NEC II vs NEC III |  | NA | 0.703 (0.572-0.835) | 94.7 | 42.5 |
| **Modified Albumin (MA)** | Yakut et al. (11) | Prospective Case Control | n= 73 Preterm infants with 37 NEC cases and 36 controls, GA<32 weeks, BW<1500g | n=20 Preterm NEC II cases | NEC II vs NECIII | Diagnostic | 252.57 (pmol/ml) | 0.815 | 89.5 | 64 |
|  |  |  |  |  |  | Post-diagnostic (d=3) | 278.68 (pmol/ml) | 0.933 | 94.7 | 84 |
|  |  |  |  |  |  | Post-diagnostic (d=7) | 268 (pmol/ml) | 0.935 | 94.7 | 92 |
| **VOC** | Meij et al. (28) | Multicentre Prospective | n=128 Preterm infants with 13 NEC cases, 31 Sepsis, and 84 controls. GA<32weeks | n=84 Preterm Healthy infants without NEC or Sepsis, Matched for GA and BW | NEC II/III vs Controls | Diagnostic | NA | 0.99 | 88.9 | 88.9 |
|  |  |  |  |  |  | Pre-diagnostic (d=1) | NA | 0.99 | 88.9 | 88.9 |
| **VOC** |  |  |  |  |  | Pre-diagnostic (d=2-3) | NA | 0.77 | 83.3 | 75 |
|  |  |  |  |  |  | Pre-diagnostic (d=4-5) | NA | 0.65 | 60 | 60 |
| **Sodium** | Zhang et al. (2024) | Retrospective Cohort | n=249 Preterm Neonates with 22 NEC I, 91 NEC II, and 136 NEC III cases, BW<2000g, GA<36weeks | n= 113 Preterm Neonates with NEC I/II | NEC II vs NEC III | Diagnostic | ≤135 mmol/L | 0.875 | 0.84 | 0.8 |
| **FTCD** | Mackay et al. (14) | Prospective | n=18 Preterm and Term infants with 12 NEC cases and 6 matched controls | n=6 Preterm and Term infants wihtout NEC | NEC II/III vs Controls | Diagnostic | NA | 0.793 (0.589–0.997) | NA | NA |
| **EFNA** |  |  |  |  |  |  | NA | 0.785 (0.589–0.981) | NA | NA |
| **GCG** |  |  |  |  |  |  | NA | 0.860 (0.6994–1.00) | NA | NA |
| **CGA CGB** |  |  |  |  |  |  | NA | 0.752 (0.539–0.966) | NA | NA |
| **HRV** | Al-Shargabi et al. (29) | Prospective Cohort | n=30 Preterm neonates with 16 NEC II and 14 NEC III cases | n=30 Preterm neonates with 16 NEC II and 14 NEC III cases | NEC II/NEC III vs | Pre-diagnostic (d=4) | NA | Drops below 0.7 threshold in around 48 hr | NA | NA |
|  |  |  |  | n=30 Preterm neonates with 16 NEC II and 14 NEC III cases | NEC II vs NEC III | Post-diagnostic (d=4) | NA | Jumps back to 0.7 in 60 hr | NA | NA |
|  |  |  |  | n=30 Preterm neonates with 16 NEC II and 14 NEC III cases | NEC II/NEC III vs | Pre-diagnostic (d=4) | NA | Drops below 0.8 in around 24 hr | NA | NA |
|  |  |  |  | n=30 Preterm neonates with 16 NEC II and 14 NEC III cases | NEC II/NEC III vs | Post-diagnostic (d=4) | NA | Jumps back to 0.7 in 60 hr | NA | NA |
|  | Doheny et al. (30) | Prospective Cohort | n=70, Preterm infants no longer requiring Ventilation past day 5 postnataly, GA (28-35weeks) | (n=70), 61 Preterm neonates, Mean GA (31.92) 28-35.30 weeks, Mean BW (1791g) 1070-2803g | NEC II/III vs Controls | Pre-diagnostic (d=5-7) | 4.68 ms^2 | 0.9  (0.8-0.992) | 88.9 | 86.9 |
| **rSO2 (Regional Tissue Oxygen Saturation)** | Schat et al. (31) | Prospective Observational Cohort | n=33 preterm infants including 20 NEC II/III and 13 Healthy infants without NEC, GA<35.9 weeks, BW<2400g | n=10 Preterm infants with NEC II | NEC II vs NECIII | Post-diagnostic | 71% | 0.88 (0.64-1.00) | 100 | 80 |
| **FTOE (Fractional Tissue Oxygenation Extraction)** |  |  |  |  |  |  | 59% | 1.00 (1.00-1.00) | 100 | 100 |
| **Panel (IL-8, IL-24, CCL20)** | Dong et al. (2023) | Prospective Case Contol | n=88, Preterm Neonates with 30 NEC cases (including NEC II (18) and NEC III (12), 29 Sepsis, and 29 controls, BW<2500g, GA<37weeks | n=29 Premterm Healthy neonates without NEC or other infectious disease | NEC II/III vs Controls | Diagnostic | NA | 0.909 (0.825-1.00) | 100 | 85 |
|  |  |  |  | n=30 Pretem Neonates with NEC II (18) or NEC III (10) | NEC II vs NEC III | Diagnostic | NA | 0.919 (0.824-1.00) | 100 | 50 |
| **Panel (L-FABP, I-FABP, TFF-3 (LIT Score))** | E.W.Y. Ng et al. (2013) | Case Control | n=100, Preterm infants, BW<1500g, GA<30weeks | n=80, with Septicimea (40) and Non-Sepsis/NEC (40) controls | NEC Stage >II vs Septicimea/Controls | Diagnostic | >4.5 | 0.81 (0.68-0.94) | 50 | 96 |
|  |  |  |  | n=40 with Non-Sepsis/non-NEC (40) controls | NEC Stage >II vs Controls |  | >4.5 | 0.81(0.68-0.95) | NA | NA |
|  |  |  |  | n=8, Infants with NEC II | NEC III vs NEC II |  | >4.5 | NA | 83 | 100 |
| **Panel (L-FABP, I-FABP, TFF-3 (LIT Score))** |  |  |  | n=80, with Septicimea (40) and Non-Sepsis/NEC (40) controls | NEC III vs Septicimea/Controls |  | >4.5 | NA | 83 | 96 |
|  |  |  |  |  |  |  |  |  |  |  |
|  |  |  |  |  |  |  |  |  |  |  |
|  |  |  |  |  |  |  |  |  |  |  |
| **Panel (CRP, miRNA223)** | P.C. Ng et al. (15) | Prospective Cohort | n=152, Preterm infants, GA<34weeks | n=128, Preterm infants without NEC (GA matched within 2 weeks) | NEC II/III vs Controls | Post-diagnosis (d=1) | 10mg/L and  130 copies/µg | NA | 58 | 84 |
|  |  |  |  |  |  |  |  |  |  |  |
|  |  |  |  |  |  |  |  |  |  |  |
|  |  |  |  |  |  |  |  |  |  |  |
|  |  |  |  |  |  |  |  |  |  |  |
| **Panel (CRP, miRNA451a)** |  |  |  |  |  |  | 10mg/L and  9.5 copies/µg | NA | 63 | 85 |
|  |  |  |  |  |  |  |  |  |  |  |
|  |  |  |  |  |  |  |  |  |  |  |
|  |  |  |  |  |  |  |  |  |  |  |
|  |  |  |  |  |  |  |  |  |  |  |
| **Panel (CRP and miRNA223 or CRP and mirRNA451a)** |  |  |  |  |  |  | 10mg/L and  130 copies/µg or  10mg/L and  9.5 copies/µg | NA | 67 | 81 |
|  |  |  |  |  |  |  |  |  |  |  |
|  |  |  |  |  |  |  |  |  |  |  |
|  |  |  |  |  |  |  |  |  |  |  |
|  |  |  |  |  |  |  |  |  |  |  |
| **Panel (CRP, S100A8/A9 (calprotectin))** |  |  |  |  |  |  | 10mg/L and  308 copies/µg | NA | 54 | 85 |
|  |  |  |  |  |  |  |  |  |  |  |
|  |  |  |  |  |  |  |  |  |  |  |
|  |  |  |  |  |  |  |  |  |  |  |
|  |  |  |  |  |  |  |  |  |  |  |
| **Panel (SAA, I-FABP)** | Coufal et al. (2020) | Retrospective | n=37, Preterm and Term infants admitted to Paediatric Surgery Department | n=8, Healthy Infants | NEC II/III vs Controls | Diagnostic or Post surgery | NA | 0.941 | NA | NA |
| **Panel (miRNA223, CRP)** | P.C. Ng et al. (15) | Prospective Cohort | n=152, Preterm infants, GA<34weeks | n=133, Preterm infants without NEC (GA matched within 2 weeks) | NEC Stage >II and Sepsis vs Controls | Diagnostic | 130 copies/µg feces and 10mg/L | NA | 58 | 84 |
|  |  |  |  |  |  |  |  |  |  |  |
|  |  |  |  |  |  |  |  |  |  |  |
|  |  |  |  |  |  |  |  |  |  |  |
| **Panel (miRNA451a, CRP)** |  |  |  |  |  |  | 9.5 copies/µg feces and 10mg/L | NA | 63 | 85 |
|  |  |  |  |  |  |  |  |  |  |  |
|  |  |  |  |  |  |  |  |  |  |  |
|  |  |  |  |  |  |  |  |  |  |  |
| **Panel (miRNA223 or miRNA451a and CRP)** |  |  |  |  |  |  | 130 copies/µg feces or 9.5 copies/µg feces and 10mg/L | NA | 67 | 81 |
|  |  |  |  |  |  |  |  |  |  |  |
|  |  |  |  |  |  |  |  |  |  |  |
|  |  |  |  |  |  |  |  |  |  |  |
| **Panel (Calprotectin (S100A8/A9), CRP)** |  |  |  |  |  |  | 308 copies/µg  feces and 10mg/L | NA | 54 | 85 |
|  |  |  |  |  |  |  |  |  |  |  |
|  |  |  |  |  |  |  |  |  |  |  |
|  |  |  |  |  |  |  |  |  |  |  |
|  |  |  |  |  |  |  |  |  |  |  |
|  |  |  |  |  |  |  |  |  |  |  |
| **Panel (RELMb, Platelet)** | Luo et al. (2019) | Case Control | n=58, Preterm infants | n=29, preterm infants matached for GA and GW | NEC II/III vs Controls | Diagnostic | 378.3 (ng/L)/157 (x10^9/L) | NA | 82.89 | 93.21 |
|  |  |  |  |  |  |  |  |  |  |  |
| **Panel (RELM-b with Abdominal Tenderness or Gurading)** | Xiao Chen Leu et al. (2022) | Prospective Cohort | n=49, Preterm and Term Neonates with 26 NEC II and 23 NEC III cases | n=26 Preterm infants with NEC II | NEC II vs NEC III | Diagnostic | NA | 0.943 (0.891–1.000) | 82.60% | 92.3 |
|  |  |  |  |  |  |  |  |  |  |  |
| **Panel (ASML1, CD14, CST3, PEDF, RET4, VASN)** | Sylvester et al. (2014) | Multiyear Prospective | n=119, Premature infants | n=26, NEC III diagnosied premature infants | NEC II vs NEC III | Diagnostic | 89 | 0.984 | 89 | 90 |
| **Panel (RIPK3, Lactic Acid, CRP)** | Shen et al. (2024) | Prospective | n=108 Preterm Neonates with 42 NEC cases (inlcuding NEC (24) and NEC III (18) and 66 controls, BW<1500g, GA<30weeks | n=66 Preterms matached for GA and BW | NEC II/III vs controls | Diagnostic | NA | 0.925 (0.88-0.97) | 90 | 80 |
| **Panel (SAA, Platelet)** | Reisinger et al. (3) | Cohort | n=29, Preterm infants with 13 NEC II and 16 NEC III cases, BW<1500g, GA<32 weeks | n=13 Preterm infants with diaognosed NEC II | NEC II vs NEC III | Diagnostic followed by surgery | NA | 0.93 (0.81–1.04) | 94 | 83 |
|  |  |  |  |  |  |  |  |  |  |  |
| **Panel (rintSO2, PCT)** | Meng et al. (4) | Retrospective Cohort | n=122 Preterm Neonates with 79 NEC I/II cases and 43 NEC III cases | n=79 Preterm infants with NEC I/II | NEC II vs NEC III | Pre-diagnostic | 0.459 | 0.97 | 93 | 90.7 |
|  |  |  |  |  |  |  |  |  |  |  |
| **Panel (rintSO2, MPV)** |  |  |  |  |  |  | 0.424 | 0.892 | 83.7 | 83.7 |
|  |  |  |  |  |  |  |  |  |  |  |
| **Panel (PCT, MPV)** |  |  |  |  |  |  | 0.221 | 0.935 | 84.8 | 93 |
|  |  |  |  |  |  |  |  |  |  |  |
| **Panel (rintSO2, PCT, MPV)** |  |  |  |  |  |  | 0.731 | 0.986 | 97.7 | 90.7 |
| **Panel (HBD2, Claudin-3)** | Xiao Chen et al. (2022) | Prospective | n=60, Preterm and Term neonates with 33 NEC II and 27 NEC III cases | n=33 Preterm and Term neonates with NEC II | NEC II vs NEC III | Diagnostic | NA | 0.805 (0.689-0.922) | 90 | 80 |
| **Panel (WBC, Platelet)** | Yu et al. (2) | Retrospective | n=84 Preterm and Term Neonates with 43 NEC II and 41 NEC III cases | n=84 Preterm and Term Neonates with 43 NEC II and 41 NEC III cases | NEC II vs NEC III | Post-diagnostic | NA | 0.84 (0.77-0.92) | 85 | NA |
|  |  |  |  |  |  |  |  |  |  |  |
| **Panel (Normogram: PVG, IMV, PLT, PH,-7.35) Training. Set** | Yixian Chen et al. (2024) | Retrospective | n=282 Preterm and Term Neonates | n=282 Preterm and Term Neonates | NEC II vs NEC III | Diagnostic | NA | 0.852 (0.794–0.910) | NA | NA |
|  |  |  |  |  |  |  |  |  |  |  |
| **Panel (Normogram: PVG, IMV, PLT, PH,-7.35) Validation Set** |  |  |  |  |  |  | NA | 0.873 (0.792–0.955) | NA | NA |
|  |  |  |  |  |  |  |  |  |  |  |
| **Panel (Model: ultrasound markers, respiratory and hemodynamic instability, abdominal wall cellulitis, and C- reactive protein > 16 mg/L)** | El Sayed et al. (32) | Case Control | n=111 Preterm neonates with 20 NEC II cases, 37 NEC III, and 54 Controls, GA<35weeks, | n=54 Preterm infants without NEC | NEC II vs NEC III | Post-Diagnostic | NA | 0.89 (0.83-0.94) | NA | NA |
|  |  |  |  |  |  |  |  |  |  |  |
| **Metabolite Panel (Tyrosine, Arginine, Riboflavin)** | Thomaidou A et al. (33) | Prospective Case Control | n=30 Preterm neonates with 15 NEC cases (including 5 NEC I and 10 NEC II/III) and 15 matched controls | n=15 Preterm healthy infants without NEC, Sepsis, or anyother condition | NEC II/III vs Controls | Diagnostic | NA | 0.963 (0.812–1.00) | NA | NA |
|  |  |  |  |  |  |  |  |  |  |  |
| **Panel (A2ML1, CD14, CST3, PEDF, RET4, VASN)** | Sylvester et al. (2014) | Multicentre Prospective | n=119 Preterm Neoantes with 85 NEC cases (inlclduding NEC II (59) NEC III (26)), 17 Sepsis cases, and 17 controls | n=59 Preterm infants with NEC II | NEC II vs NECIII | Diagnostic | NA | 0.984 | 89 | 90 |
|  |  |  |  |  |  |  |  |  |  |  |
| **Panel (A2ML1, CD14, CST3, PEDF, RET4, VASN)** | Sylvester et al. (2014) | Multicentre Prospective | n=119 Preterm Neoantes with 85 NEC cases (inlclduding NEC II (59) NEC III (26)), 17 Sepsis cases, and 17 controls | n=17 Preterm Healthy controls with matched GA and BW | NEC II/III vs Controls | Diagnostic | NA | 0.997 | 96 | 90 |
|  |  |  |  |  |  |  |  |  |  |  |
| **Panel (Isoleucine, Lysine, Ethanolamine, Tryptophan, Ornithine)** | Deinova et al. (34) | Prospective Case Control | n=62 Preterm infants with 31 NEC III cases and 31 matched controlsGA<30weeks | n=31 Preterm healthy infants without NEC III | NEC III vs Controls | Pre-diagnostic (d=1-3) | NA | 0.67 | NA | NA |
| **Panel (miRNA, CRP)** | Ng et al. (16) | Prospective Cohort | n=301 Preterm infants including NEC, Septicimea, Non-NEC, and Healthy infantss | Preterm Non-NEC and Healthy infants | NEC II/III vs Controls | Post-Diagnostic (d=1) | miRNA >650 copies/µL OR >220 to 650 copies AND CRP >15.8mg/L | NA | 83 | 96 |
| **MDAS Score** | Fijas et al. (35) | Retrospective Cohort | n= 64 VLBW preterm neonates with 44 NEC II cases and 20 NEC III cases | n=44 Preterm infants with NEC II | NEC II vs NECIII | Diagnostic | 2 | 0.77 (0.65–0.89) | 70 | 66 |
|  | Ibáñez et al. (36) | Retrospective Cohort | n=99 Preterm infants with 61 NEC II cases and 14 NEC III cases | n=99 Preterm infants with 61 NEC II cases and 14 NEC III cases | NEC II vs NECIII | Diagnostic | NA | 0.59 (0.47–0.71) | NA | NA |
|  |  |  |  | n=99 Preterm infants with 61 NEC II cases and 14 NEC III cases |  | Post-diagnostic | NA | 0.64 (0.52–0.77) | NA | NA |
| **SNAPPE II Score** | Fijas et al. (32) | Retrospective Cohort | n= 64 VLBW preterm neonates with 44 NEC II cases and 20 NEC III cases | n=44 Preterm infants with NEC II | NEC II vs NECIII | Diagnostic | 29.04 | 0.71 (0.57–0.85) | 70 | 60 |
|  | Ibáñez et al. (33) | Retrospective Cohort | n=99 Preterm infants with 61 NEC II cases and 14 NEC III cases | n=99 Preterm infants with 61 NEC II cases and 14 NEC III cases | NEC II vs NECIII | Diagnostic | NA | 0.69 (0.57–0.80) | NA | NA |
|  |  |  |  | n=99 Preterm infants with 61 NEC II cases and 14 NEC III cases |  | Post-Diagnostic | NA | 0.67 (0.55–0.80) | NA | NA |
|  |  |  |  |  |  |  |  |  |  |  |
| **Doppler flowmetry of Superior Mesenteric Artery (Resistivity Index RI)** | Urboniene et al. (37) | Prospective | n=62 Preterm and term infants with 29 NEC cases (including NEC I (13) NEC II (12) and NEC III (4)) and 33 controls | n=33 Preterm and term controls without NEC | NEC II/III vs Controls | Diagnostic | >0.75 | 0.93 | 96.3 | 90.9 |
|  |  |  |  |  |  |  |  |  |  |  |
| **Doppler flowmetry of Superior Mesenteric Artery (Pulsatility Index PI)** |  |  |  |  |  |  | >1.85 | 0.85 | 88.9 | 78.8 |
|  |  |  |  |  |  |  |  |  |  |  |
| **Doppler flowmetry of Portal Vein (Systolic Velocity/SV)** |  |  |  |  |  |  | 17.5 cm/s | 0.65 | 75.9 | 66.7 |
|  |  |  |  |  |  |  |  |  |  |  |
| **Doppler flowmetry of Portal Vein (Mean Flow Velocity/MFV)** |  |  |  |  |  |  | 10.8 cm/s | 0.6 | 69 | 63.6 |
|  |  |  |  |  |  |  |  |  |  |  |
| **Doppler flowmetry of Portal Vein (Volumetric Blood Flow/VBF)** |  |  |  |  |  |  | 37 ml/min | 0.75 | 89.7 | 57.6 |

References

Luo, J., Li, H. P., Xu, F., Wu, B. Q., & Lin, H. C. (2019). Early diagnosis of necrotizing enterocolitis by plasma RELMβ and thrombocytopenia in preterm infants: A pilot study. *Pediatrics and neonatology*, *60*(4), 447–452. <https://doi.org/10.1016/j.pedneo.2019.01.006>

Yu, M., Liu, G., Feng, Z., & Huang, L. (2018). Combination of plasma white blood cell count, platelet count and C-reactive protein level for identifying surgical necrotizing enterocolitis in preterm infants without pneumoperitoneum. *Pediatric surgery international*, *34*(9), 945–950. <https://doi.org/10.1007/s00383-018-4305-6>

Reisinger, K. W., Kramer, B. W., Van der Zee, D. C., Brouwers, H. A., Buurman, W. A., van Heurn, E., & Derikx, J. P. (2014). Non-invasive serum amyloid A (SAA) measurement and plasma platelets for accurate prediction of surgical intervention in severe necrotizing enterocolitis (NEC). *PloS one*, *9*(6), e90834. <https://doi.org/10.1371/journal.pone.0090834>

Meng, W., Wang, Q., Xu, Q., Gao, H., Zhou, Y., & Shao, W. (2024). Biomarkers in the Severity of Necrotizing Enterocolitis in Preterm Infants: A Pilot Study. *International journal of general medicine*, *17*, 1017–1023. <https://doi.org/10.2147/IJGM.S446378>

Chen, Y., Lan, C., Zhong, W., Song, K., Ma, Z., Huang, L., Zhu, Y., & Xia, H. (2023). Plasma anti-myosin autoantibodies in the diagnosis of necrotizing enterocolitis. *European journal of pediatrics*, *182*(11), 5203–5210. <https://doi.org/10.1007/s00431-023-05188-6>

Moroze, M., Morphew, T., Sayrs, L. W., Eghbal, A., Holmes, W. N., Shafer, G., & Mikhael, M. (2024). Blood absolute monocyte count trends in preterm infants with suspected necrotizing enterocolitis: an adjunct tool for diagnosis?. *Journal of perinatology : official journal of the California Perinatal Association*, *44*(12), 1768–1773. <https://doi.org/10.1038/s41372-024-02070-7>

Desiraju, S., Bensadoun, J., Bateman, D., & Kashyap, S. (2020). The role of absolute monocyte counts in predicting severity of necrotizing enterocolitis. *Journal of perinatology : official journal of the California Perinatal Association*, *40*(6), 922–927. <https://doi.org/10.1038/s41372-020-0596-2>

Remon, J., Kampanatkosol, R., Kaul, R. R., Muraskas, J. K., Christensen, R. D., & Maheshwari, A. (2014). Acute drop in blood monocyte count differentiates NEC from other causes of feeding intolerance. *Journal of perinatology : official journal of the California Perinatal Association*, *34*(7), 549–554. <https://doi.org/10.1038/jp.2014.52>

Yang, Y., Cao, Z. L., Zhou, X. Y., Chen, X. Q., Pan, J. J., & Cheng, R. (2019). Does neutrophil/lymphocyte ratio have good diagnostic value in neonatal necrotizing colitis?. *The journal of maternal-fetal & neonatal medicine : the official journal of the European Association of Perinatal Medicine, the Federation of Asia and Oceania Perinatal Societies, the International Society of Perinatal Obstetricians*, *32*(18), 3026–3033. <https://doi.org/10.1080/14767058.2018.1455182>

Wisgrill, L., Weinhandl, A., Unterasinger, L., Amann, G., Oehler, R., Metzelder, M. L., Berger, A., & Benkoe, T. M. (2019). Interleukin-6 serum levels predict surgical intervention in infants with necrotizing enterocolitis. *Journal of pediatric surgery*, *54*(3), 449–454. <https://doi.org/10.1016/j.jpedsurg.2018.08.003>

Yakut, I., Tayman, C., Oztekin, O., Namuslu, M., Karaca, F., & Kosus, A. (2014). Ischemia-modified albumin may be a novel marker for the diagnosis and follow-up of necrotizing enterocolitis. *Journal of clinical laboratory analysis*, *28*(3), 170–177. <https://doi.org/10.1002/jcla.21661>

Benkoe, T., Reck, C., Pones, M., Weninger, M., Gleiss, A., Stift, A., & Rebhandl, W. (2014). Interleukin-8 predicts 60-day mortality in premature infants with necrotizing enterocolitis. *Journal of pediatric surgery*, *49*(3), 385–389. <https://doi.org/10.1016/j.jpedsurg.2013.05.068>

Almonaem, E. R. A., Almotaleb, G. S. A., Alhameed, M. H. A., & El-Shimi, O. S. (2022). Utility of transforming growth factor beta-1 in diagnosis of neonatal necrotizing enterocolitis. *Journal of neonatal-perinatal medicine*, *15*(4), 795–801. <https://doi.org/10.3233/NPM-210973>

Mackay S, Frazer LC., Bailey GK., Miller CM., Gong Q, Dewitt ON., Singh DK. and Good M (2023) Identification of serum biomarkers for necrotizing enterocolitis using aptamer-based proteomics. Front. Pediatr. 11:1184940. doi: 10.3389/fped.2023.1184940

Ng, P. C., Chan, K. Y. Y., Lam, H. S., Wong, R. P. O., Ma, T. P. Y., Sit, T., Leung, K. T., Chan, L. C. N., Pang, Y. L. I., Cheung, H. M., Chu, W. C. W., & Li, K. (2020). A Prospective Cohort Study of Fecal miR-223 and miR-451a as Noninvasive and Specific Biomarkers for Diagnosis of Necrotizing Enterocolitis in Preterm Infants. *Neonatology*, *117*(5), 555–561. <https://doi.org/10.1159/000511655>

Ng, P. C., Chan, K. Y. Y., Yuen, T. P., Sit, T., Lam, H. S., Leung, K. T., Wong, R. P. O., Chan, L. C. N., Pang, Y. L. I., Cheung, H. M., Chu, W. C. W., & Li, K. (2019). Plasma miR-1290 Is a Novel and Specific Biomarker for Early Diagnosis of Necrotizing Enterocolitis-Biomarker Discovery with Prospective Cohort Evaluation. *The Journal of pediatrics*, *205*, 83–90.e10. <https://doi.org/10.1016/j.jpeds.2018.09.031>

Barekatain B, Saneian H, Ebrahimi A, Mahaki B. Evaluation and comparison of stool calprotectin level in necrotizing enterocolitis infected and noninfected neonates of &#60;1500 g [Original Article]. *Journal of Clinical Neonatology*. 2019 April 1, 2019;8(2):90-95. doi:10.4103/jcn.JCN_106_18.

1. Terrin G, Passariello A, De Curtis M, Paludetto R, Berni Canani R. S100 A8/A9 protein as a marker for early diagnosis of necrotising enterocolitis in neonates. *Arch Dis Child*. 2012 Dec;97(12):1102. eng. Epub 2012/10/23. doi:10.1136/archdischild-2012-302698. Cited in: Pubmed; PMID 23087190.

Pickering, A., White, R., & Davis, N. L. (2016). Routine fecal occult blood testing does not predict necrotizing enterocolitis in very low birth weight neonates. *Journal of neonatal-perinatal medicine*, *9*(2), 171–178. <https://doi.org/10.3233/NPM-16915120>

Gómez-Chaparro Moreno, J. L., Rodríguez Torronteras, A., Ruiz González, M. D., Izquierdo Palomares, L., Bonilla Valverde, D., Ruiz Laguna, J., Delgado Rubio, A., & López-Barea, J. (2016). The β-glucosidase assay: a new diagnostic tool for necrotizing enterocolitis. Sensitivity, specificity, and predictive values. *European journal of pediatrics*, *175*(7), 931–941. <https://doi.org/10.1007/s00431-016-2724-8>

Gregory, K. E., Winston, A. B., Yamamoto, H. S., Dawood, H. Y., Fashemi, T., Fichorova, R. N., & Van Marter, L. J. (2014). Urinary intestinal fatty acid binding protein predicts necrotizing enterocolitis. *The Journal of pediatrics*, *164*(6), 1486–1488. <https://doi.org/10.1016/j.jpeds.2014.01.057>

Shaaban, A. I. E., Alfqy, O. A. E., Shaaban, H. M. K., A-Maqsoud, Y. H., & Assar, E. H. (2021). Potential Role of Serum Intestinal Fatty Acid-Binding Protein as a Marker for Early Prediction and Diagnosis of Necrotizing Enterocolitis in Preterm Neonates. *Journal of Indian Association of Pediatric Surgeons*, *26*(6), 393–400. <https://doi.org/10.4103/jiaps.JIAPS_218_20>

Al-banna, E. A., Abd El-Rehman, H. M., Kamel, L. M. M., & Ahmed, A. E. M. (2020). Intestinal Fatty-Acid-Binding Protein in Neonatal Necrotizing Enterocolitis. *The Egyptian Journal of Hospital Medicine*, *80*(2), 809-814. doi: 10.21608/ejhm.2020.98912

Jawale, N., Prideaux, M., Prasad, M., Miller, M., Rastogi, S., & for Maimonides Neonatal Group (2021). Plasma Citrulline as a Biomarker for Early Diagnosis of Necrotizing Enterocolitis in Preterm Infants. *American journal of perinatology*, *38*(13), 1435–1441. <https://doi.org/10.1055/s-0040-1713406>

Celik, I. H., Demirel, G., Canpolat, F. E., & Dilmen, U. (2013). Reduced plasma citrulline levels in low birth weight infants with necrotizing enterocolitis. *Journal of clinical laboratory analysis*, *27*(4), 328–332. <https://doi.org/10.1002/jcla.21607>

1. Du T-T, Liu X-C, He Y, Gao X, Liu Z-Z, Wang Z-L and Li L-Q (2023) Changes of gut microbiota and tricarboxylic acid metabolites may be helpful in early diagnosis of necrotizing enterocolitis: A pilot study. Front. Microbiol. 14:1119981. doi: 10.3389/fmicb.2023.1119981

Sharif, S. P., Friedmacher, F., Amin, A., Zaki, R. A., Hird, M. F., Khashu, M., & Phelps, S. R. (2020). Low serum albumin concentration predicts the need for surgical intervention in neonates with necrotizing enterocolitis. *Journal of pediatric surgery*, *55*(12), 2625–2629. <https://doi.org/10.1016/j.jpedsurg.2020.07.003>

de Meij, T. G., van der Schee, M. P., Berkhout, D. J., van de Velde, M. E., Jansen, A. E., Kramer, B. W., van Weissenbruch, M. M., van Kaam, A. H., Andriessen, P., van Goudoever, J. B., Niemarkt, H. J., & de Boer, N. K. (2015). Early Detection of Necrotizing Enterocolitis by Fecal Volatile Organic Compounds Analysis. *The Journal of pediatrics*, *167*(3), 562–7.e1. <https://doi.org/10.1016/j.jpeds.2015.05.044>

Al-Shargabi, T., Reich, D., Govindan, R. B., Shankar, S., Metzler, M., Cristante, C., McCarter, R., Sandler, A. D., Said, M., & Plessis, A. D. (2018). Changes in Autonomic Tone in Premature Infants Developing Necrotizing Enterocolitis. *American journal of perinatology*, *35*(11), 1079–1086. <https://doi.org/10.1055/s-0038-1639339>

Doheny, K. K., Palmer, C., Browning, K. N., Jairath, P., Liao, D., He, F., & Travagli, R. A. (2014). Diminished vagal tone is a predictive biomarker of necrotizing enterocolitis-risk in preterm infants. *Neurogastroenterology and motility*, *26*(6), 832–840. <https://doi.org/10.1111/nmo.12337>

Schat, T. E., Schurink, M., van der Laan, M. E., Hulscher, J. B., Hulzebos, C. V., Bos, A. F., & Kooi, E. M. (2016). Near-Infrared Spectroscopy to Predict the Course of Necrotizing Enterocolitis. *PloS one*, *11*(5), e0154710. <https://doi.org/10.1371/journal.pone.0154710>

Elsayed, Y., & Seshia, M. (2022). A new intestinal ultrasound integrated approach for the management of neonatal gut injury. *European journal of pediatrics*, *181*(4), 1739–1749. <https://doi.org/10.1007/s00431-021-04353-z>

Thomaidou, A., Chatziioannou, A. C., Deda, O., Benaki, D., Gika, H., Mikros, E., Agakidis, C., Raikos, N., Theodoridis, G., & Sarafidis, K. (2019). A pilot case-control study of urine metabolomics in preterm neonates with necrotizing enterocolitis. *Journal of chromatography. B, Analytical technologies in the biomedical and life sciences*, *1117*, 10–21. <https://doi.org/10.1016/j.jchromb.2019.04.019>

Deianova N, El Manouni El Hassani S, Struijs EA, Jansen EEW, Bakkali A, van de Wiel MA, de Boode WP, Hulzebos CV, van Kaam AH, Kramer BW, d'Haens E, Vijlbrief DC, van Weissenbruch MM, de Jonge WJ, Benninga MA, Niemarkt HJ, de Boer NKH, de Meij TGJ. Fecal amine metabolite analysis before onset of severe necrotizing enterocolitis in preterm infants: a prospective case-control study. Sci Rep. 2022 Jul 19;12(1):12310. doi: 10.1038/s41598-022-16351-8. PMID: 35853977; PMCID: PMC9296556.

Fijas, M., Vega, M., Xie, X., Kim, M., & Havranek, T. (2023). SNAPPE-II and MDAS scores as predictors for surgical intervention in very low birth weight neonates with necrotizing enterocolitis. *The journal of maternal-fetal & neonatal medicine : the official journal of the European Association of Perinatal Medicine, the Federation of Asia and Oceania Perinatal Societies, the International Society of Perinatal Obstetricians*, *36*(1), 2148096. <https://doi.org/10.1080/14767058.2022.2148096>

Ibáñez, V., Couselo, M., Marijuán, V., Vila, J. J., & García-Sala, C. (2012). Could clinical scores guide the surgical treatment of necrotizing enterocolitis?. *Pediatric surgery international*, *28*(3), 271–276. <https://doi.org/10.1007/s00383-011-3016-z>

Urboniene, A., Palepsaitis, A., Uktveris, R., & Barauskas, V. (2015). Doppler flowmetry of the superior mesenteric artery and portal vein: impact for the early prediction of necrotizing enterocolitis in neonates. *Pediatric surgery international*, *31*(11), 1061–1066. <https://doi.org/10.1007/s00383-015-3792-y>
