# Supplementary material for: Necrotising enterocolitis biomarkers: a systematic review
Source: Front Pediatr. 2026 Jan 12;13:1652566. doi: 10.3389/fped.2025.1652566 (PMC12833235; doi:10.3389/fped.2025.1652566)
Supplement: Supplementary file 2 [file Table2.docx]

**Supplementary Table 1.2: Comprehensive Biomarker Profiles**

| Biomarker | Molecule Type | Trend (Stage II vs III) | Timing |
| --- | --- | --- | --- |
| Platelets | Cell count | Lower in III | Post-diagnosis (12h, diagnostic) |
| Mean Platelet Volume (MPV) | Cell measurement | Higher in III | Pre-diagnostic |
| Platelet to Lymphocyte Ratio (PLR) | Cell ratio | Higher in III | Post-diagnosis |
| White Blood Cell Count (WBC) | Cell count | Lower/variable in III | Post-diagnosis (d1) |
| Absolute Monocyte Count (AMC) | Cell count | Higher in III | Pre-/post-diagnostic (d3) |
| Neutrophil | Cell count | Higher in III | Diagnostic |
| Absolute Neutrophil Count (ANC) | Cell count | Higher in III | Post-diagnosis (d1) |
| Neutrophil CD64 | Surface marker | Higher in III | Pre-/diagnostic |
| Neutrophil to Lymphocyte Ratio (NL) | Cell ratio | Higher in III | Post-diagnostic (d1) |
| Prothrombin Time (PT) | Coagulation factor | Prolonged in III | Post-diagnostic (0.5d) |
| PT-INR | Coagulation index | Higher in III | Post-diagnostic (0.5d) |
| Activated Partial Thromboplastin Time (APTT) | Coagulation factor | Prolonged in III | Post-diagnostic (0.5d) |
| Fibrinogen | Protein | Lower in II (higher threshold in III) | Diagnostic/post-diagnostic (0.5d) |
| Fibrinogen Peptides (FGA) | Protein fragment | Higher in III | Diagnostic |
| Coagulant Factor XIII | Protein | Lower in III | Pre-diagnostic |
| TRAIL | Cytokine | Higher in III/II combined vs controls | Diagnostic |
| TSLP | Cytokine | Higher in III/II | Diagnostic |
| MCP-4 | Chemokine | Higher in III | Diagnostic |
| TNFSF14 | Cytokine | Higher in III/II | Diagnostic |
| LIF | Cytokine | Higher in III | Diagnostic |
| CCL20 | Chemokine | Higher in III | Diagnostic |
| OPG | Protein | Higher in III | Diagnostic |
| IL-6 | Cytokine | Higher in III | Diagnostic/post-diagnostic (d3,d7) |
| IL-8 | Cytokine | Higher in III | Diagnostic |
| IL-24 | Cytokine | Higher in III | Diagnostic |
| IL-33 | Cytokine | Higher over time in III | Diagnostic/post-diagnostic (d3,d7) |
| TGF-beta 1 | Cytokine | Higher in II vs controls, variable III | Diagnostic/post-diagnostic (d7) |
| RELMβ | Protein | Higher in III/II | Diagnostic |
| CD14 | Surface marker | Higher in III | Diagnostic |
| CCL16 | Chemokine | Higher in III/II | Diagnostic |
| CXCL (general) | Chemokine | Higher in III/II | Diagnostic |
| CXCL6 | Chemokine | Higher in III/II | Diagnostic |
| COLEC12 | Protein | Higher in III/II | Diagnostic |
| MICA | Protein | Higher in III/II | Diagnostic |
| CRP | Acute phase protein | Higher in III | Pre-/post-diagnostic (d1) |
| Procalcitonin (PCT) | Peptide | Higher in III | Pre-/diagnostic |
| IαIp | Protein | Higher in III | Diagnostic |
| SAA | Acute phase protein | Higher in III | Diagnostic/post-surgery^[^ |
| Fecal Calprotectin | Protein | Higher in III | Pre-diagnostic |
| Calprotectin S100A8/A9 | Protein complex | Higher in III | Diagnostic |
| TFF-3 | Protein | Higher in III | Diagnostic |
| CBG | Protein | Higher in III/II | Diagnostic |
| Gal-4 | Lectin | Higher in III | Post-diagnostic (d1-2) |
| FABP (general) | Protein | Higher in III | Pre-diagnostic (d3,d7) |
| L-FABP | Protein | Higher in III | Diagnostic |
| I-FABP | Protein | Higher in III | Diagnostic/pre-diagnostic |
| I-FABPμ | Protein variant | Higher in III | Diagnostic |
| HBD2 | Antimicrobial peptide | Higher in III | Diagnostic/pre-diagnostic |
| UDCA, GCDCA, GCA, Secondary BAs, TCA, TCDCA, DCA | Bile acids/metabolites | Altered ratio (lower primary/secondary in III) | Diagnostic |
| Claudin-3 | Protein | Higher in III | Diagnostic |
| Citrulline | Amino acid | Lower in III | Diagnostic |
| Arginine | Amino acid | Lower in III | Diagnostic |
| IAP (content/activity) | Enzyme | Lower in III | Diagnostic |
| Acetic/Propionic/Butyric Acid (SCFAs) | Metabolites | Altered (lower in III) | Pre-diagnostic |
| Succinate/L-Malic Acid/Oxaloacetate (TCA) | Metabolites | Higher in III | Prior to diagnosis |
| IGHA1/IGHA2 | Immunoglobulin | Higher in III/II | Diagnostic |
| Haptoglobin | Protein | Higher in III | Pre-diagnostic (d10) |
| Lipocalin-2 | Protein | Higher in III | Pre-diagnostic (d10) |
| Calgranulin-C (S100A12) | Protein | Higher in III | Diagnostic/pre-diagnostic (d7) |
| Lactic Acid (LA) | Metabolite | Higher in III | Diagnostic |
| Endocan | Protein | Higher in III | Diagnostic/post-diagnostic (d3,d7) |
| RIPK3 | Protein | Higher in III | Diagnostic |
| MMP10 | Metalloproteinase | Higher in III/II | Diagnostic |
| MMP13 | Metalloproteinase | Higher in III/II | Diagnostic |
| A2ML1 | Protein | Higher in III | Diagnostic |
| CST3 | Protein | Higher in III | Diagnostic |
| PEDF | Protein | Higher in III | Diagnostic |
| RET4 | Protein | Higher in III | Diagnostic |
| VASN | Protein | Higher in III | Diagnostic |
| AFP | Protein | Higher in III/II | Diagnostic |
| miRNA1290, miRNA1246, miRNA451a, miRNA375, miRNA223 | microRNA | Higher in III/II | Diagnostic/post-diagnosis (d1) |
| Urinary Caveolin-1 | Protein | Higher in III/II | Post-diagnostic (d3) |
| Hept-2-enal, Pent-1-ene-3-one, 2-Ethylfuran, Pentanal, 2-Pentylfuran | VOCs | Higher in II/III | Diagnostic/post-diagnostic |
| Albumin | Protein | Lower in III | Diagnostic/post-diagnostic (d2) |
| PA Pre-albumin | Protein | Lower in III | Post-diagnostic (d1) |
| Modified Albumin (MA) | Protein variant | Higher in III | Diagnostic/post-diagnostic (d3,d7) |
| VOC (general) | Metabolites | Altered profile in III | Pre-/diagnostic (d1-5) |
| Sodium | Electrolyte | Lower in III | Diagnostic |
| FTCD, EFNA, GCG, CGA/CGB | Proteins | Higher in III/II | Diagnostic |
| HRV | Physiologic index | Drops longer/more in III | Pre-/post-diagnostic (d4) |
| rSO2/FTOE | Oxygenation metrics | Lower extraction in III | Post-diagnostic |
| Panels (e.g., IL-8/IL-24/CCL20, L-FABP/I-FABP/TFF-3, CRP/miRNAs) | Composite | Superior discrimination (AUC>0.9) for III | Diagnostic |
