# Supplementary material for: Necrotising enterocolitis biomarkers: a systematic review
Source: Front Pediatr. 2026 Jan 12;13:1652566. doi: 10.3389/fped.2025.1652566 (PMC12833235; doi:10.3389/fped.2025.1652566)

## Slide 1
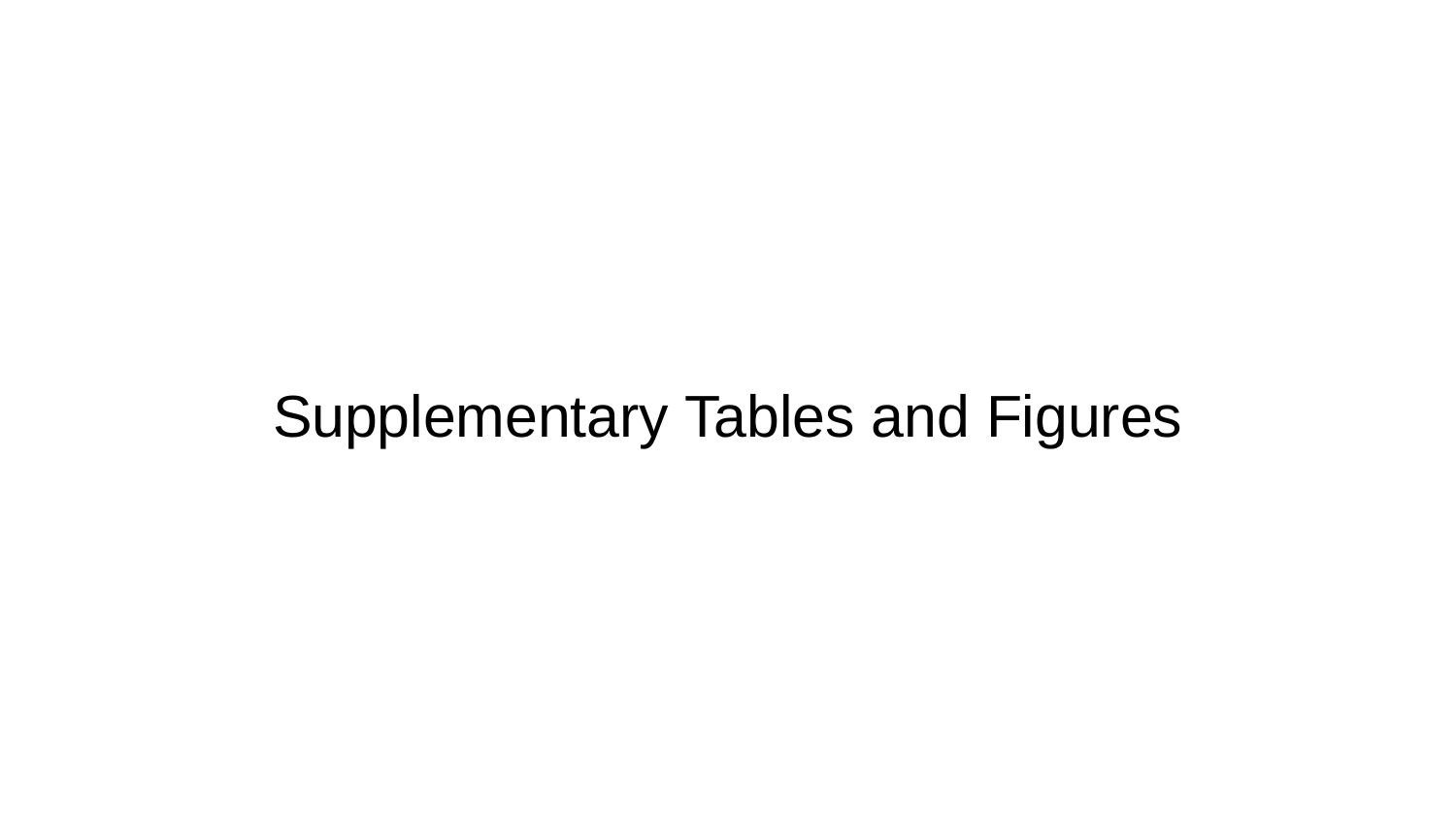

# Supplementary Tables and Figures

## Slide 2
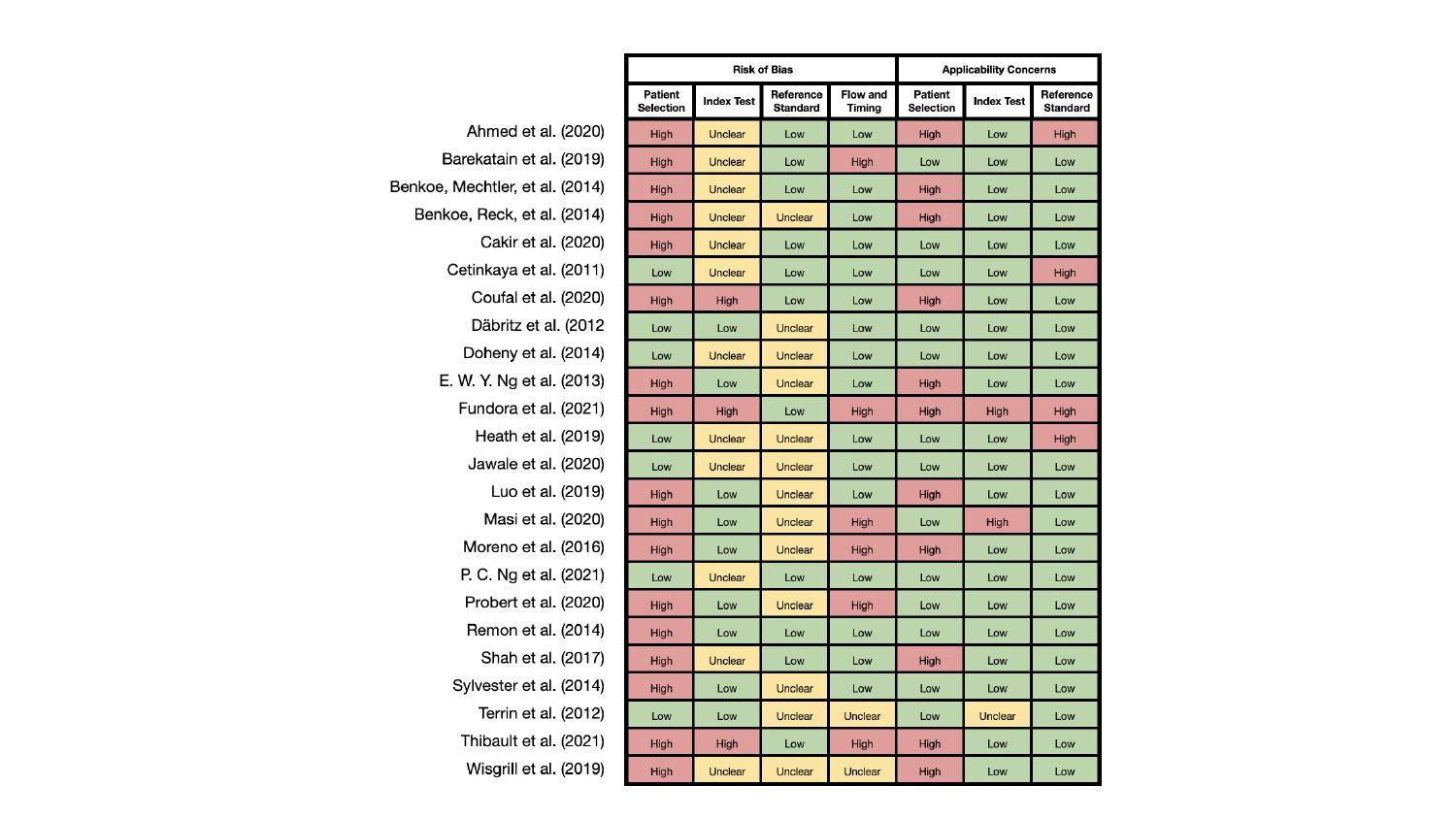

## Slide 3
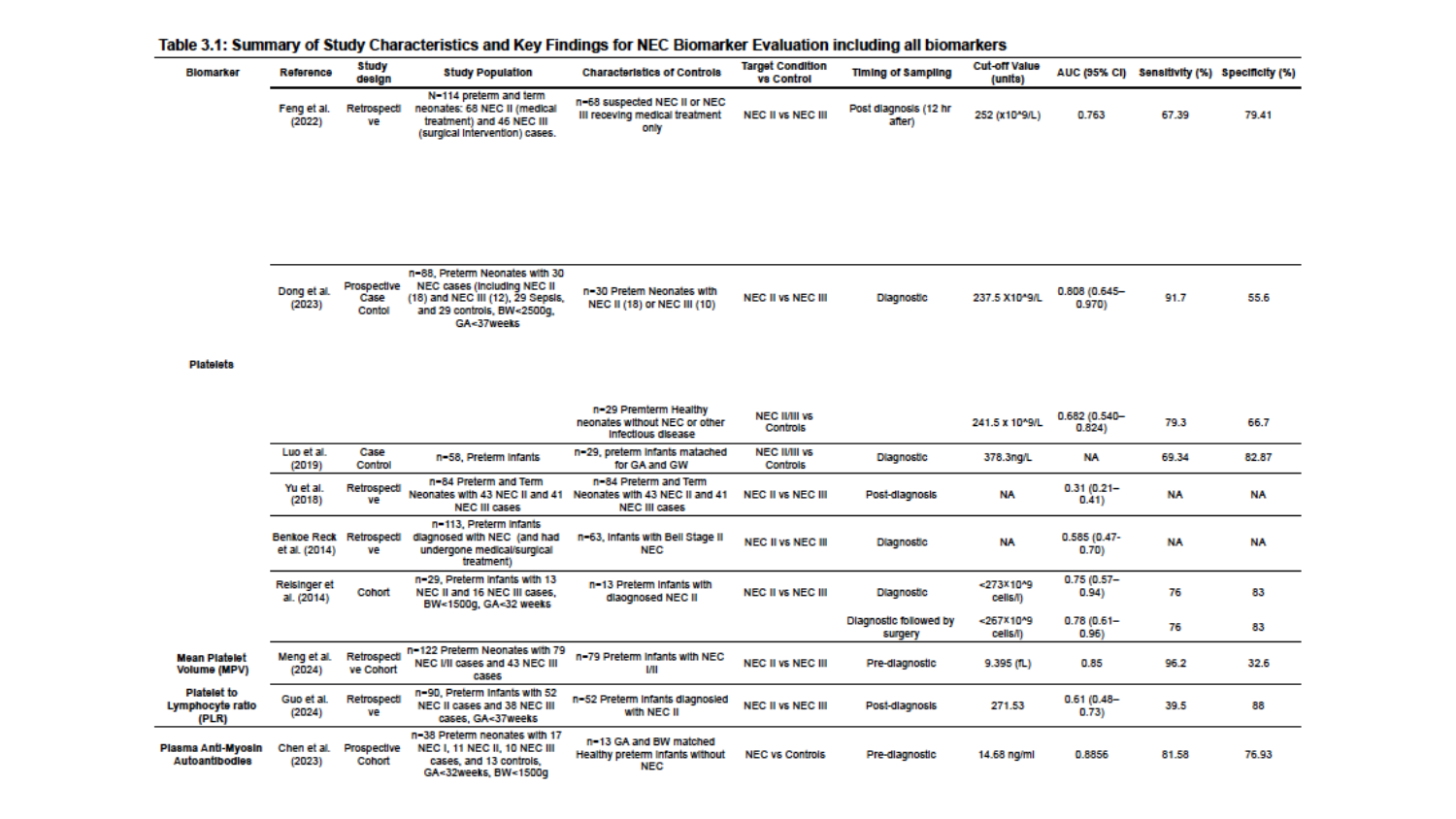

## Slide 4
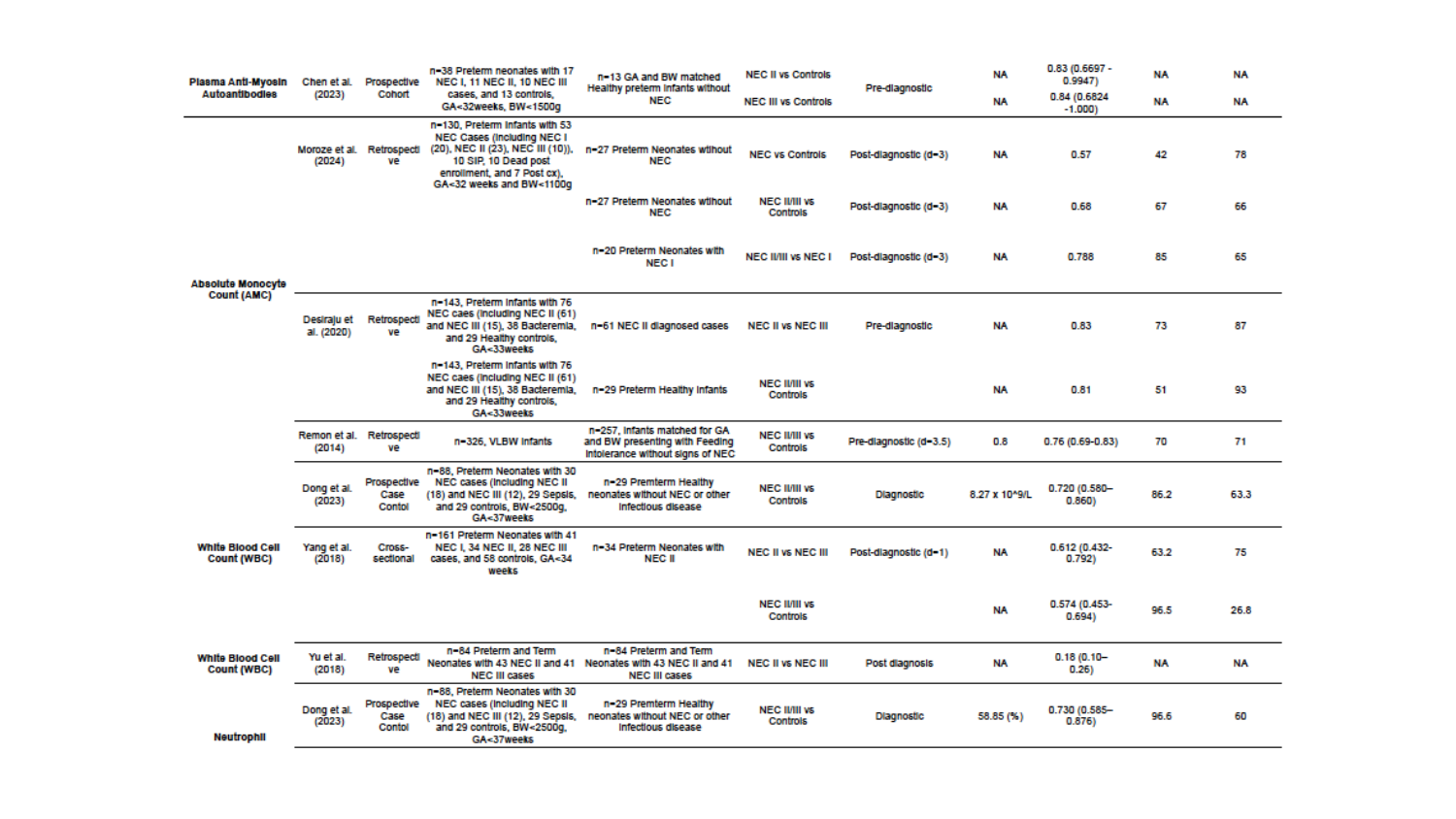

## Slide 5
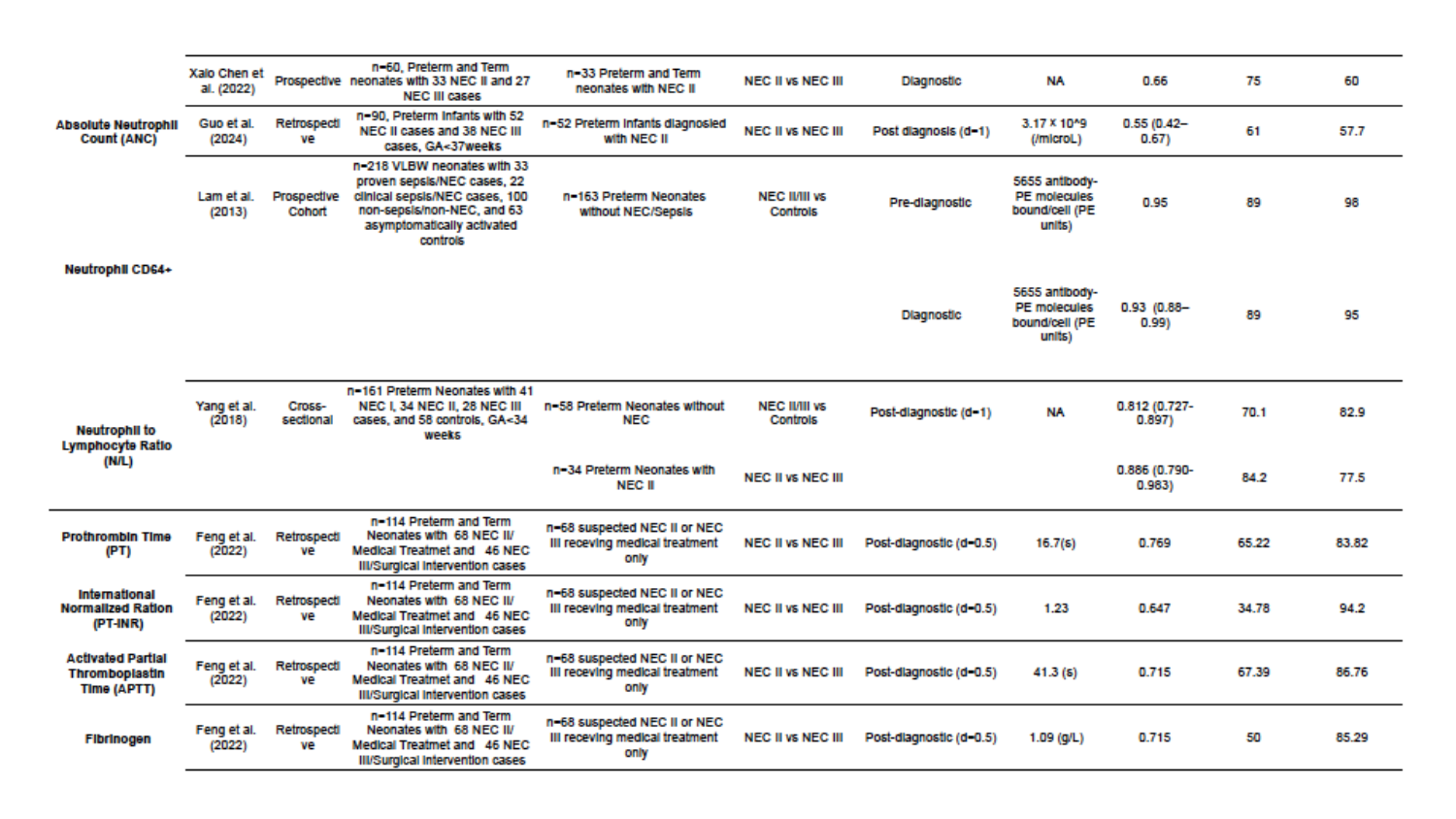

## Slide 6
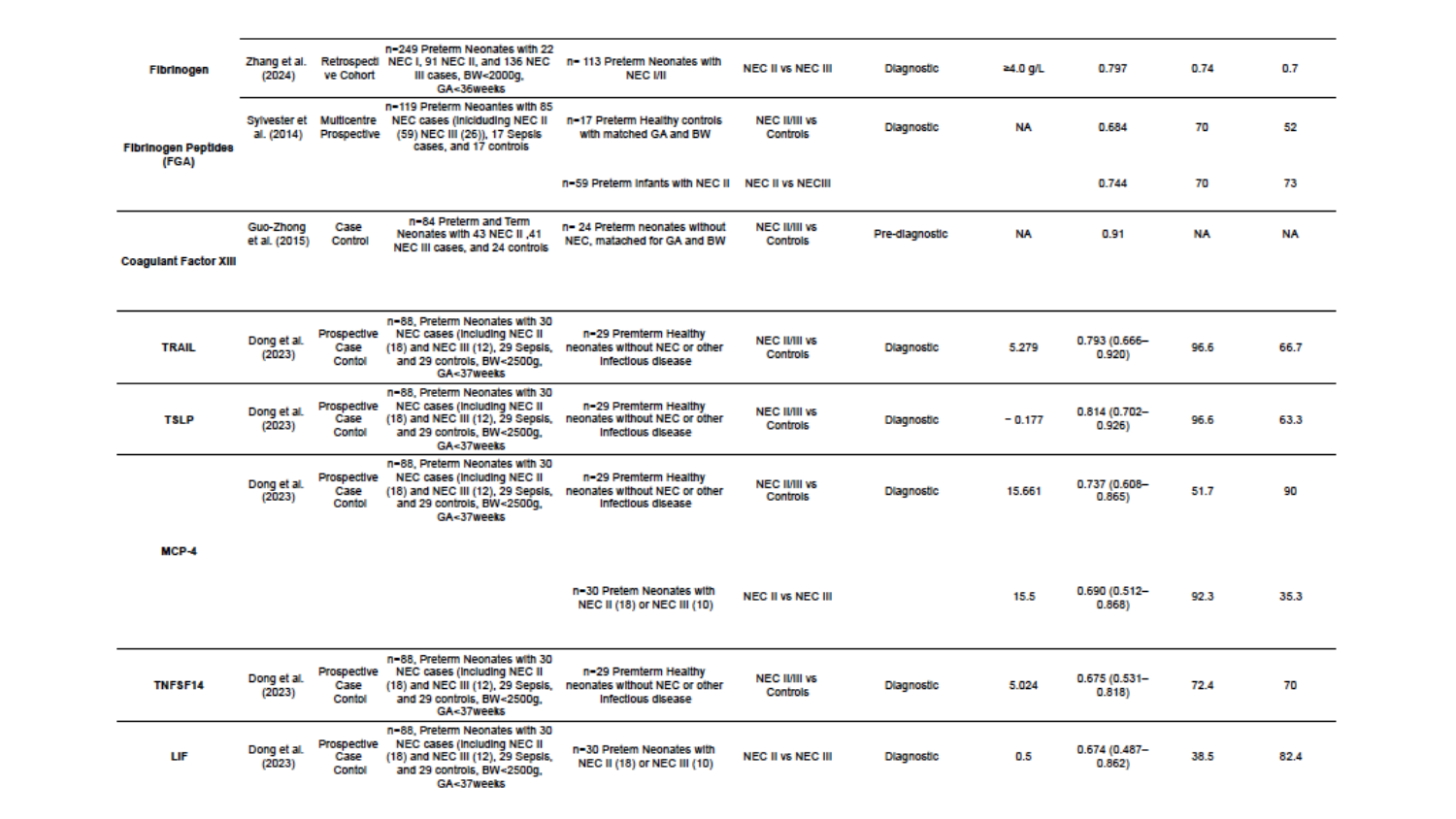

## Slide 7
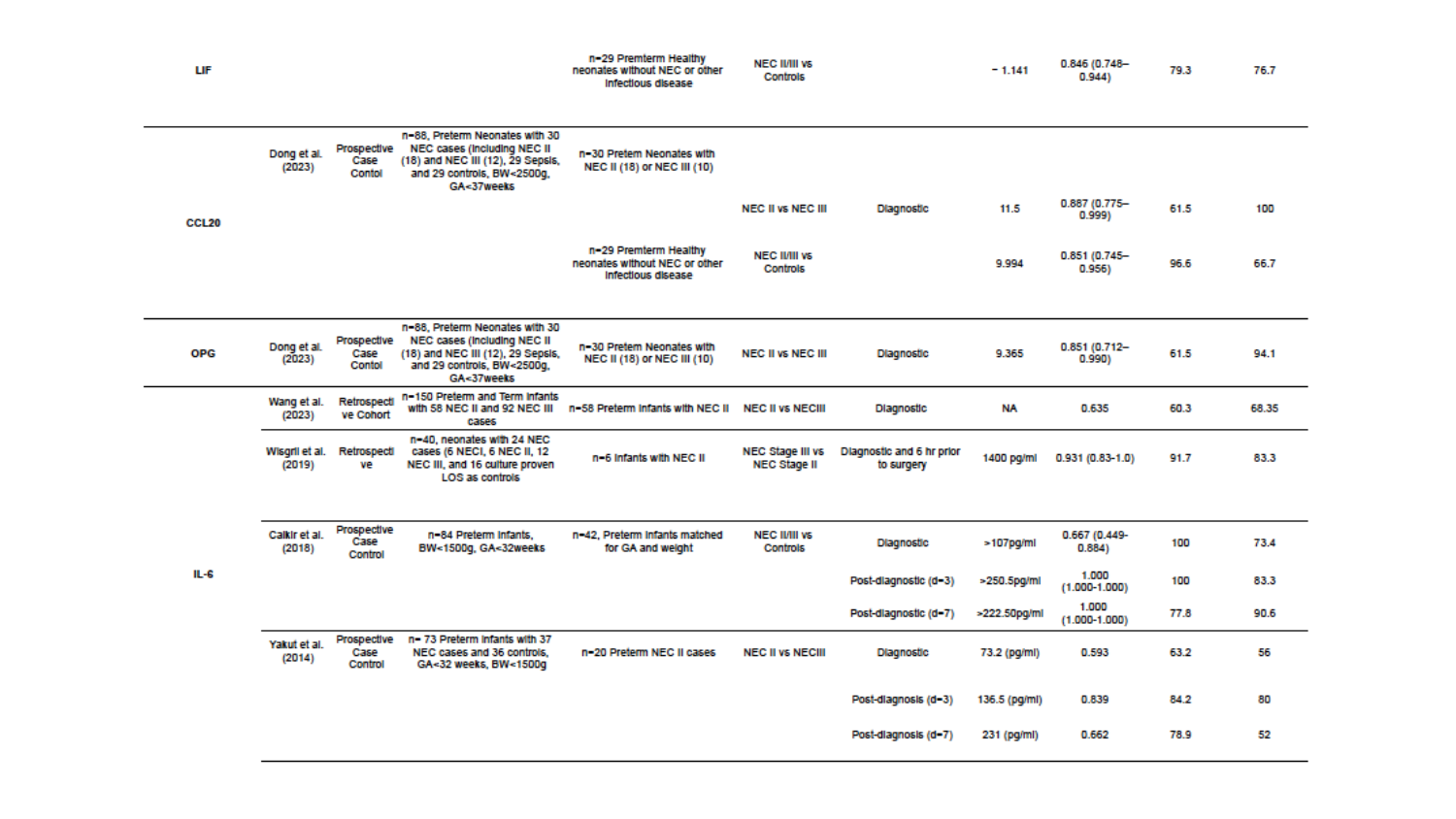

## Slide 8
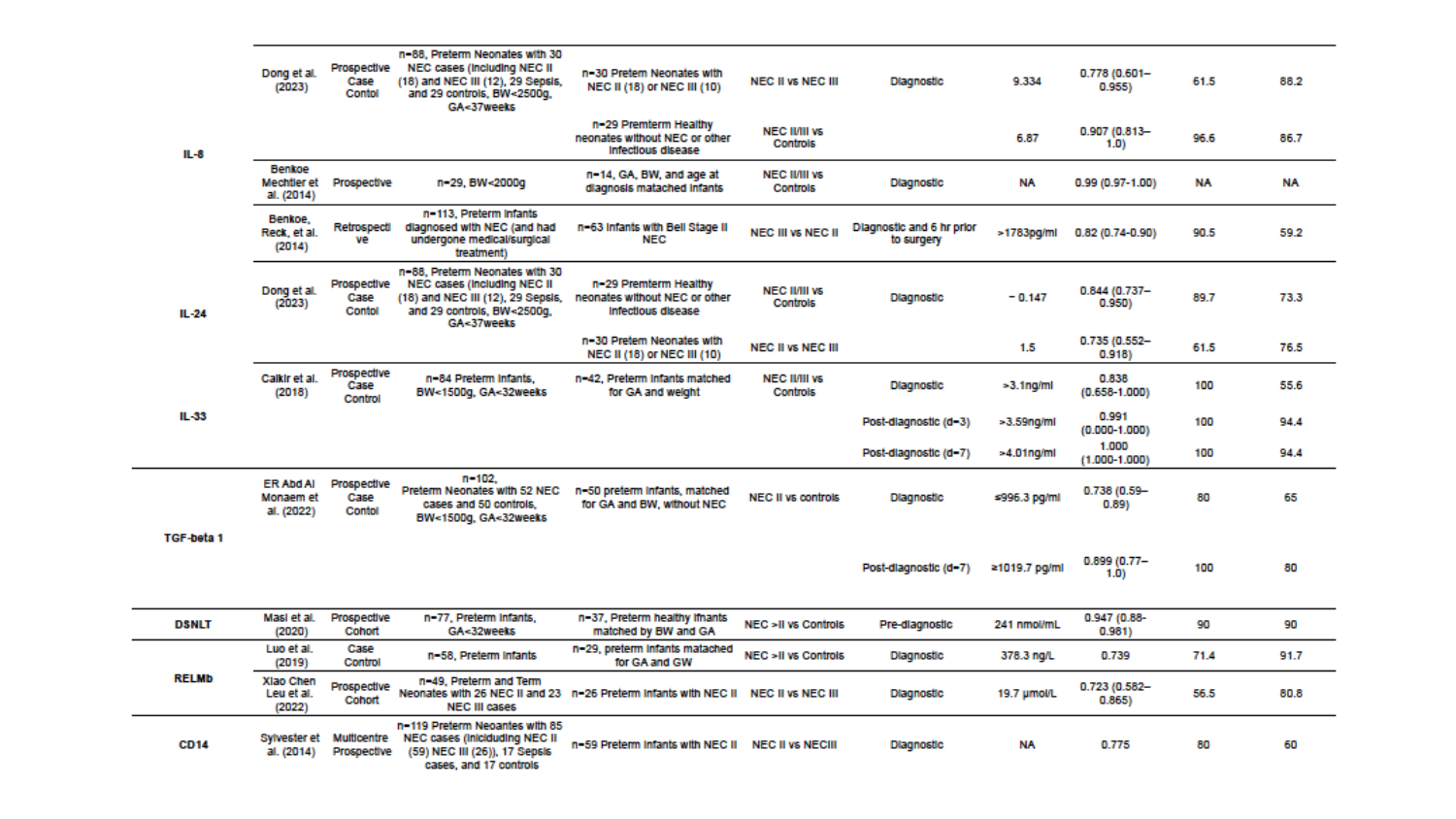

## Slide 9
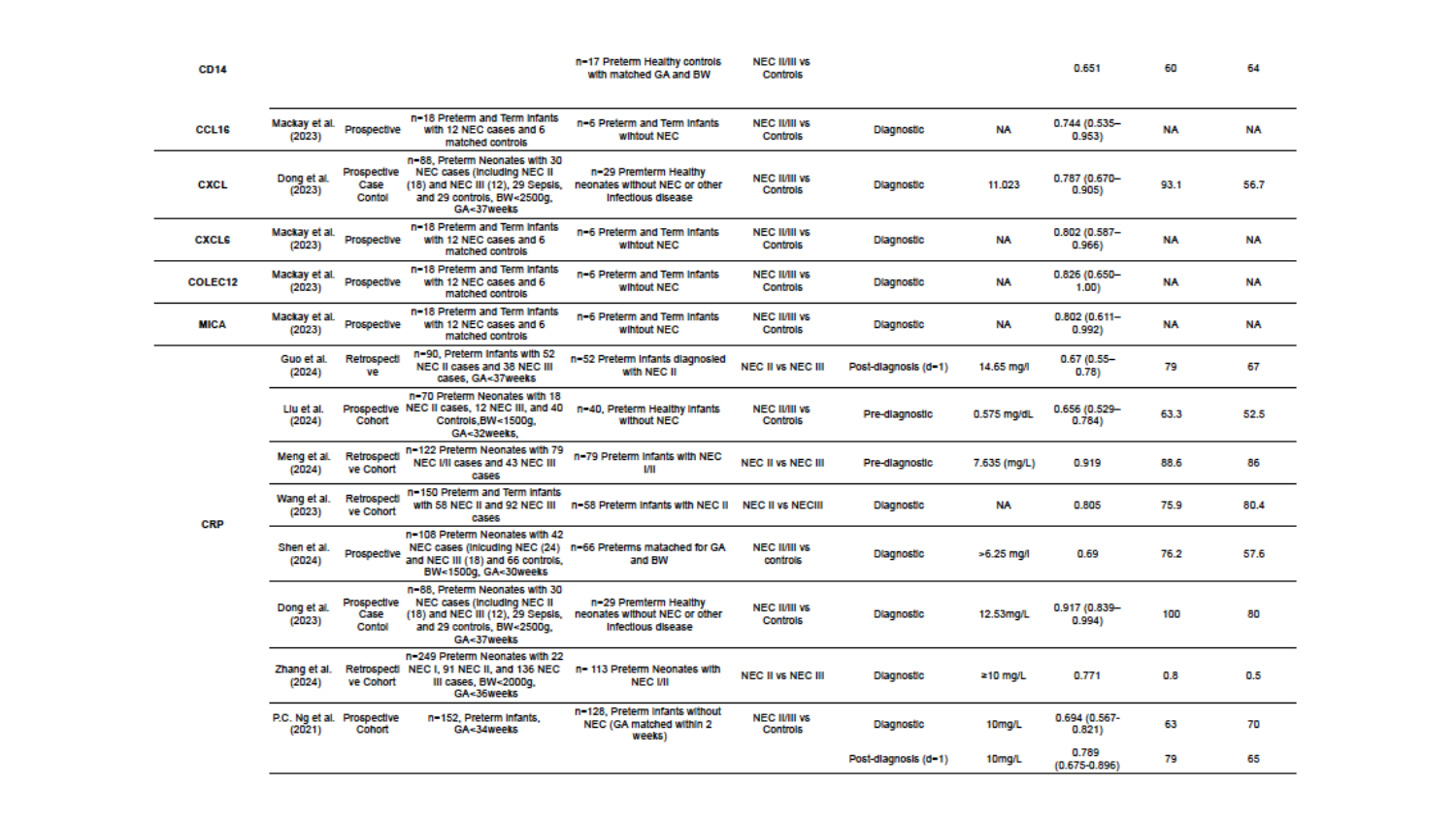

## Slide 10
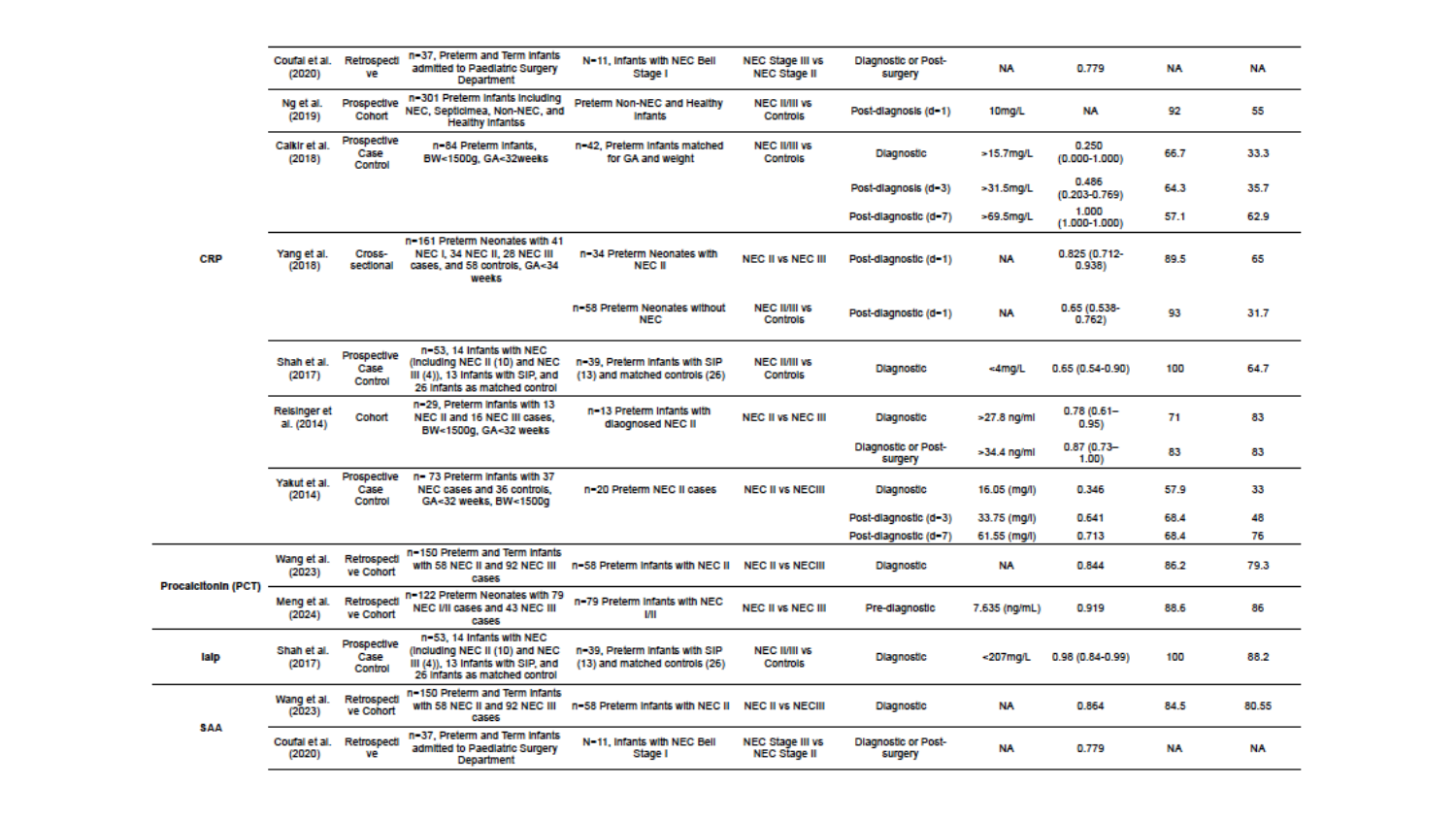

## Slide 11
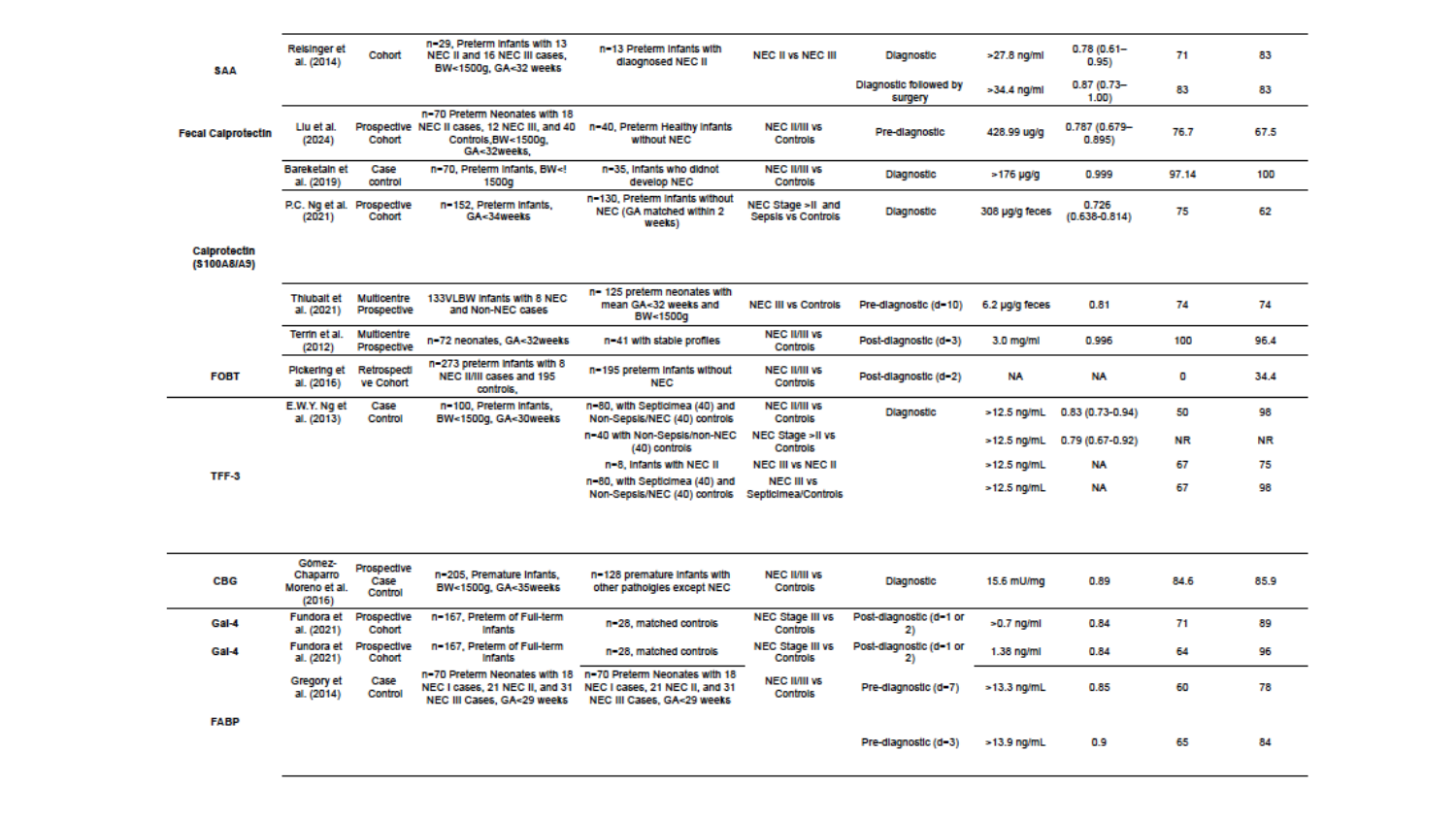

## Slide 12
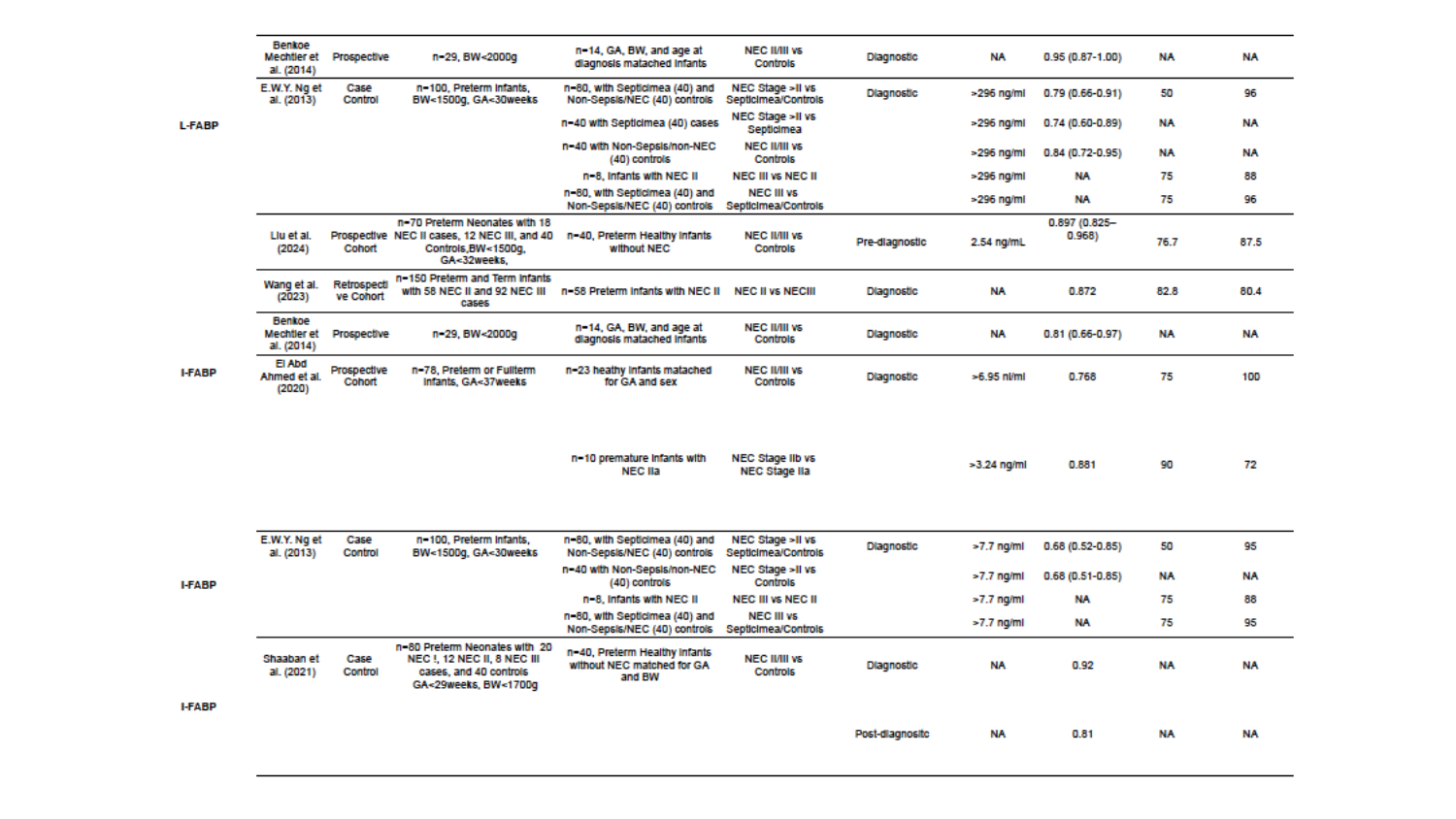

## Slide 13
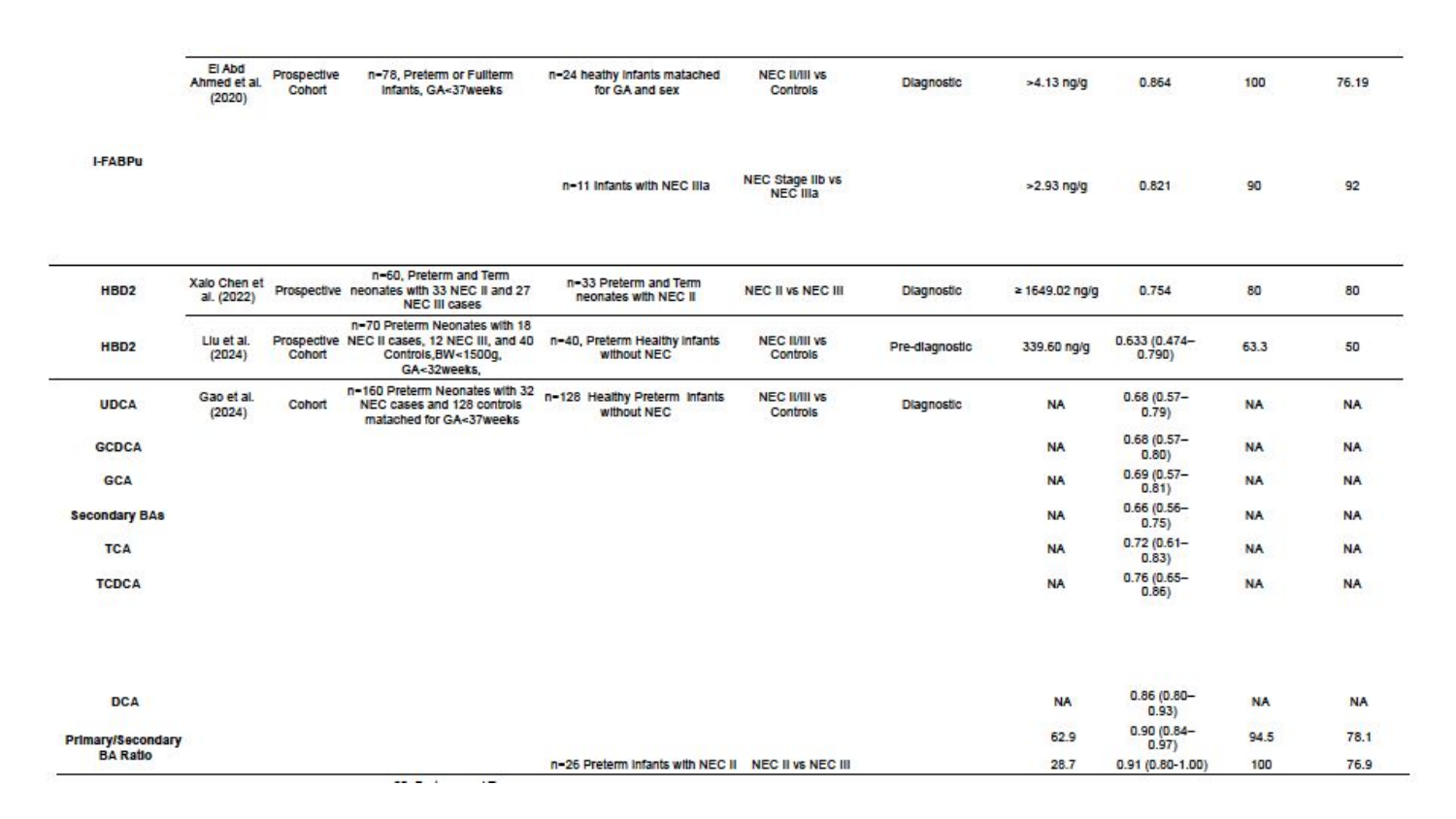

## Slide 14
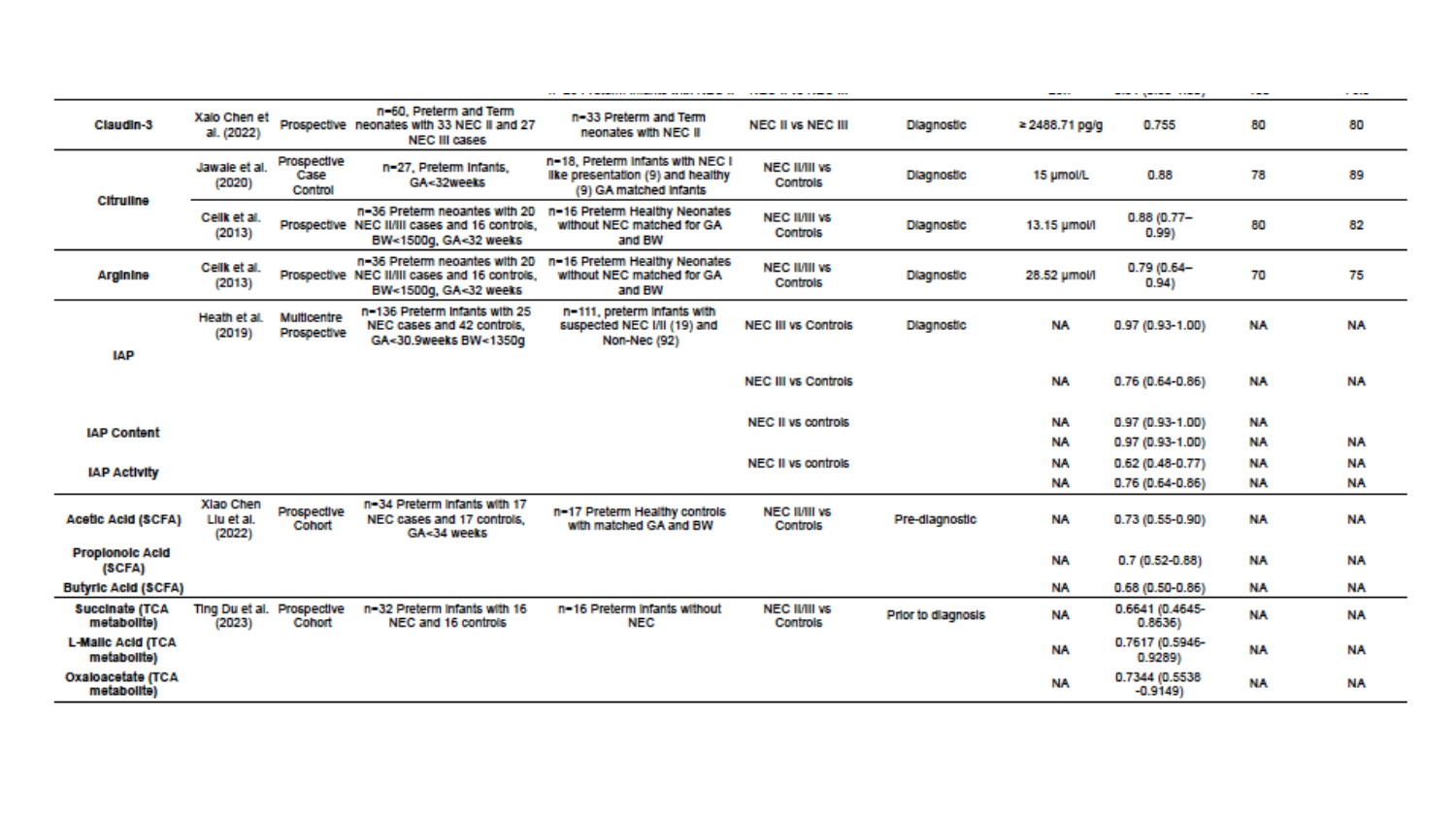

## Slide 15
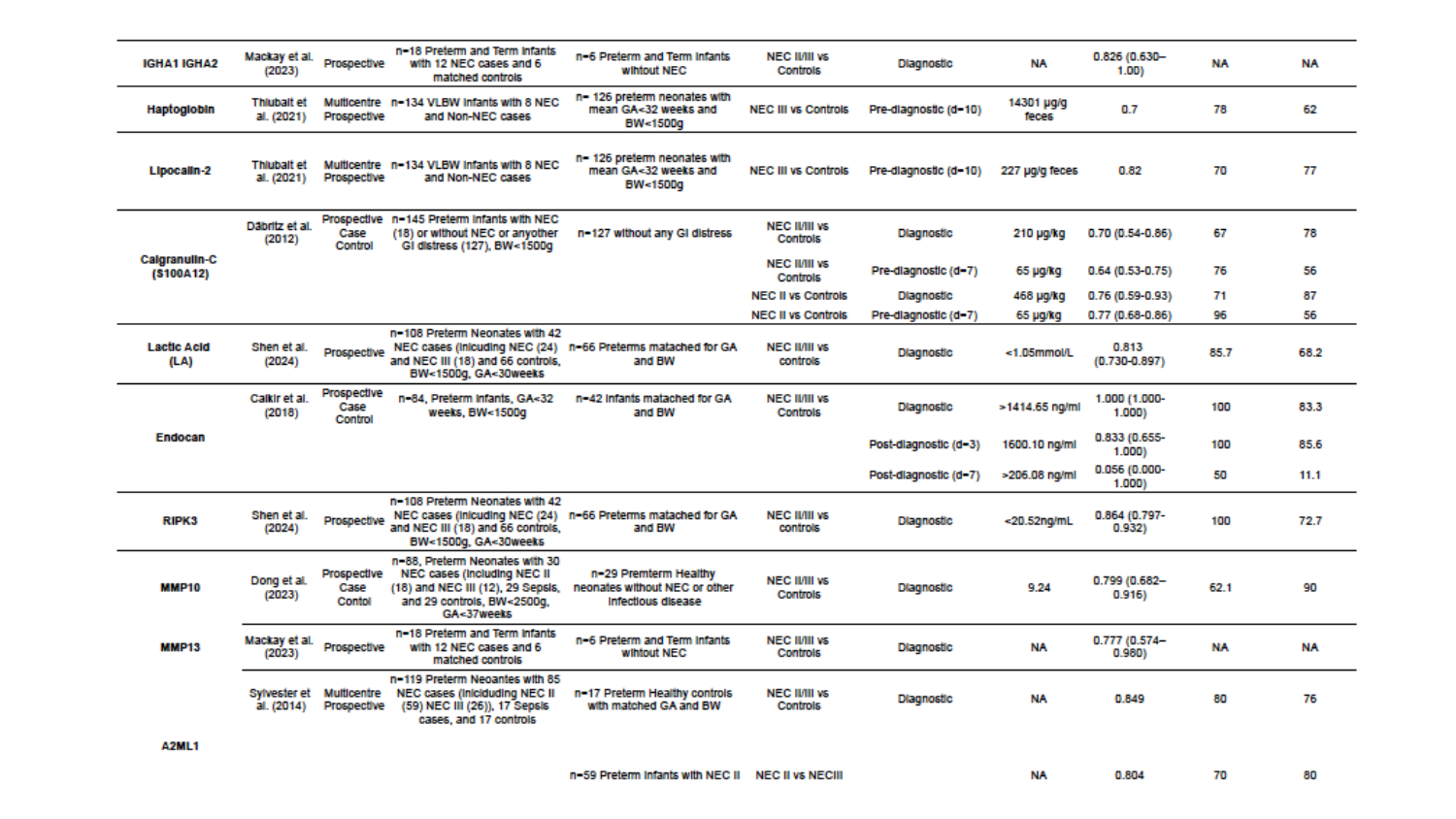

## Slide 16
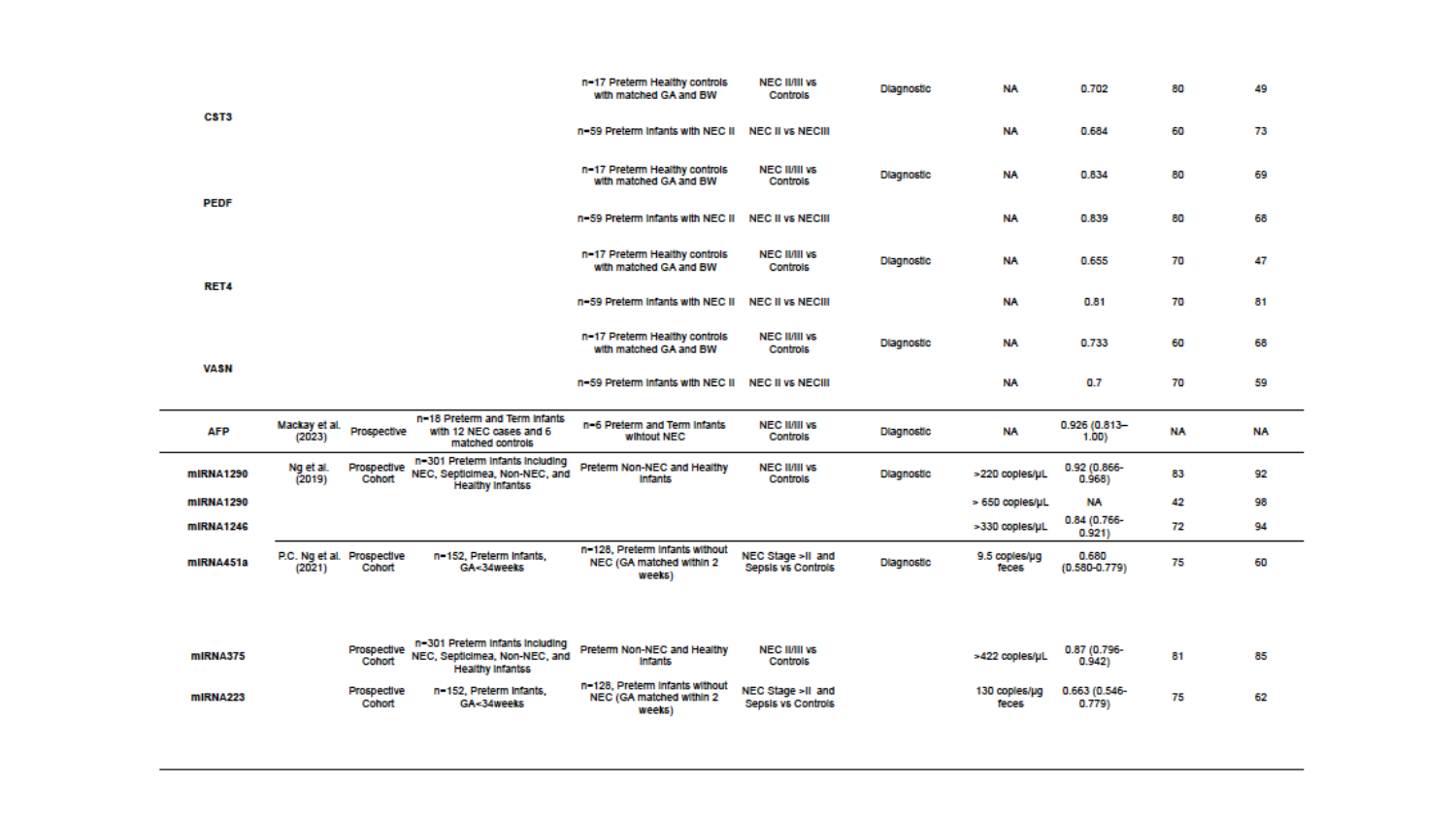

## Slide 17
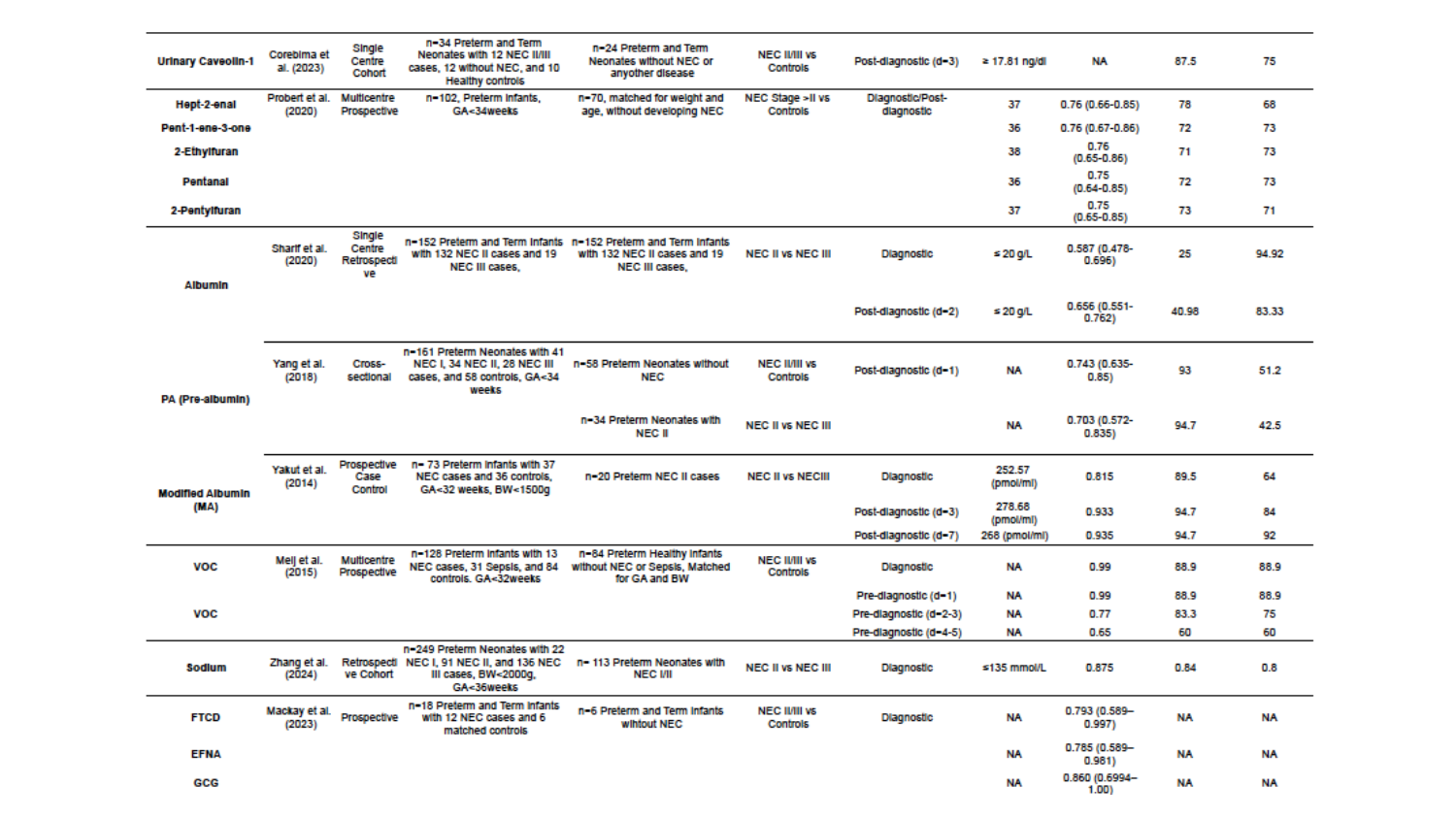

## Slide 18
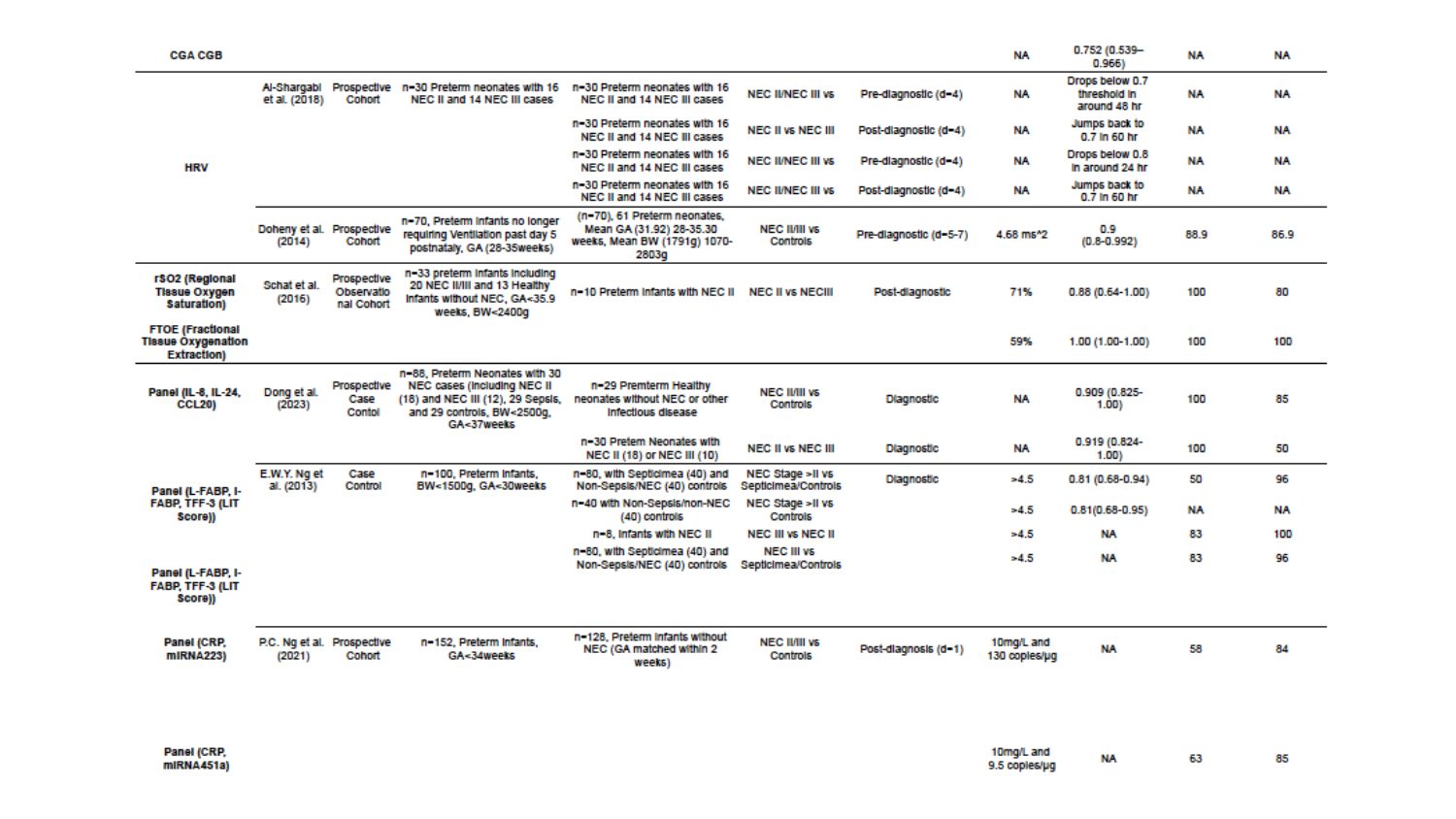

## Slide 19
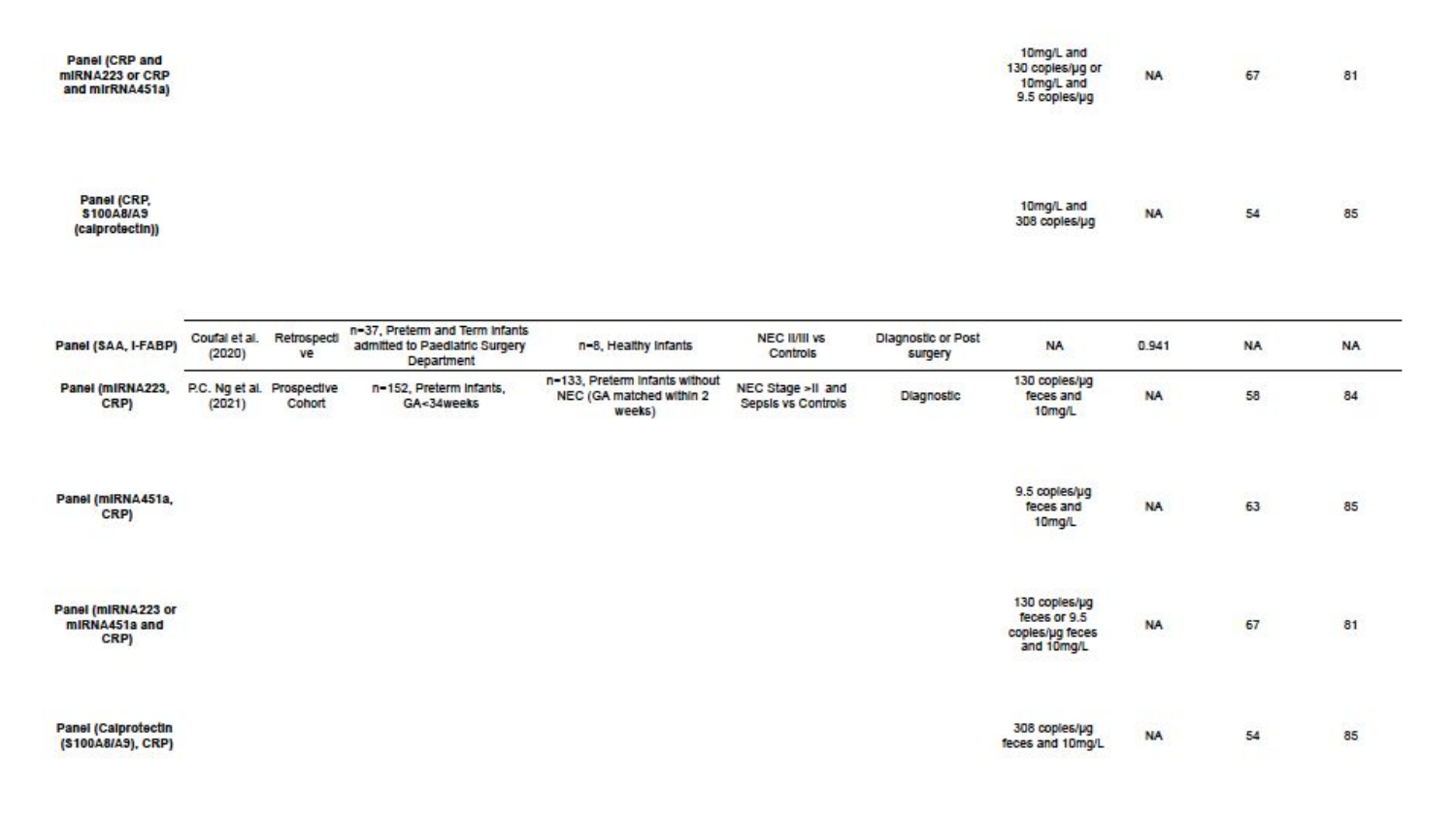

## Slide 20
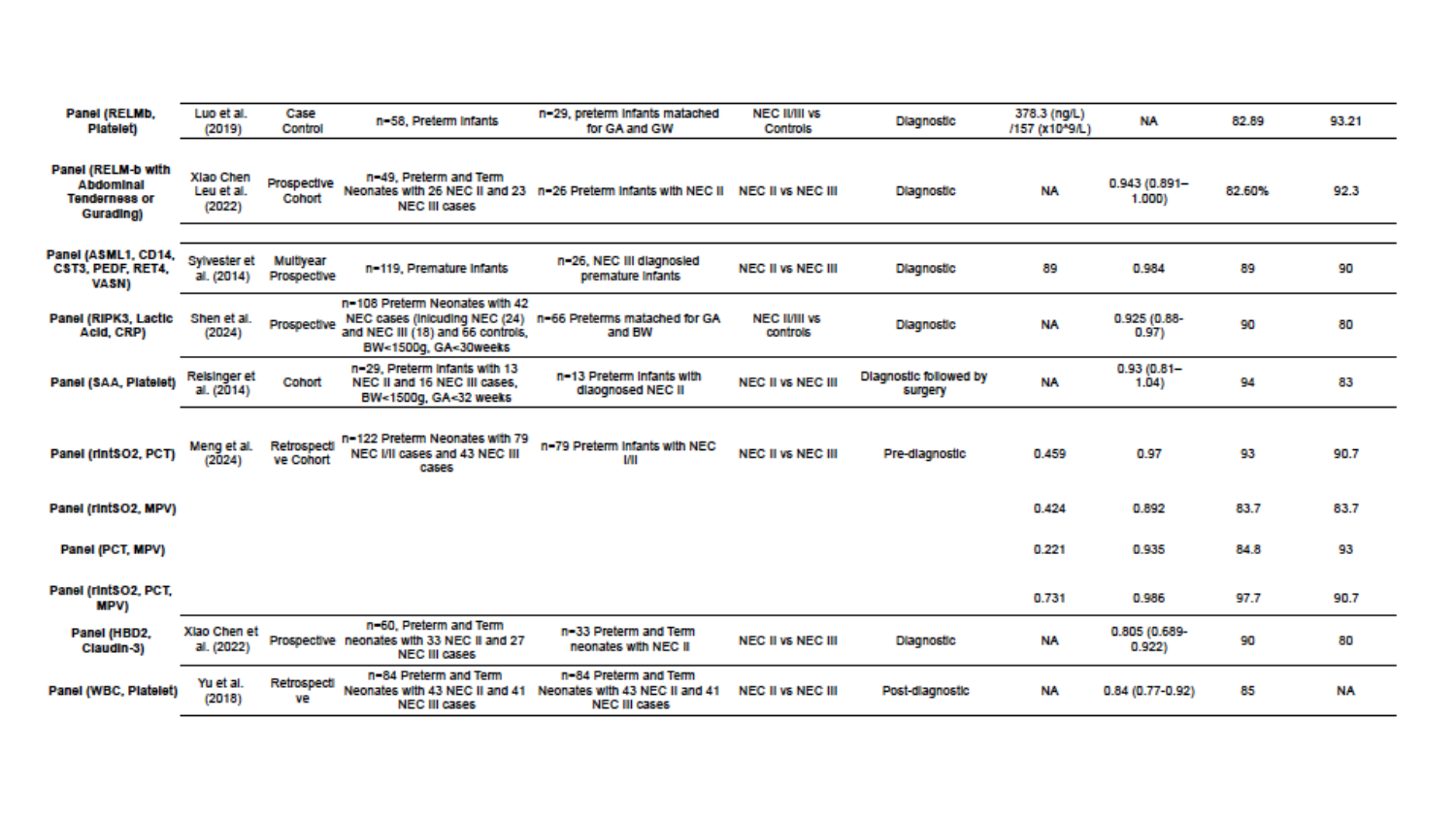

## Slide 21
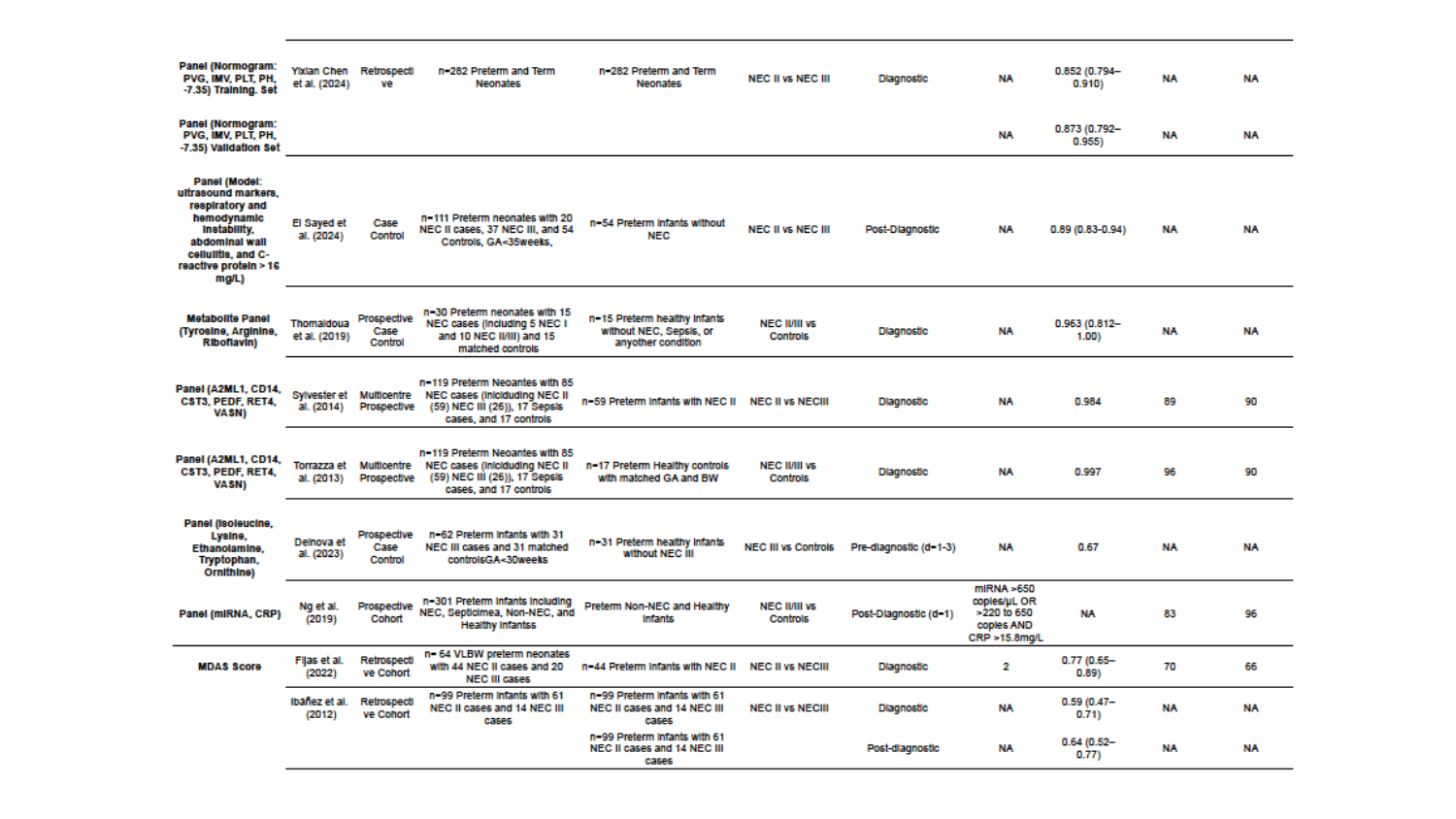

## Slide 22
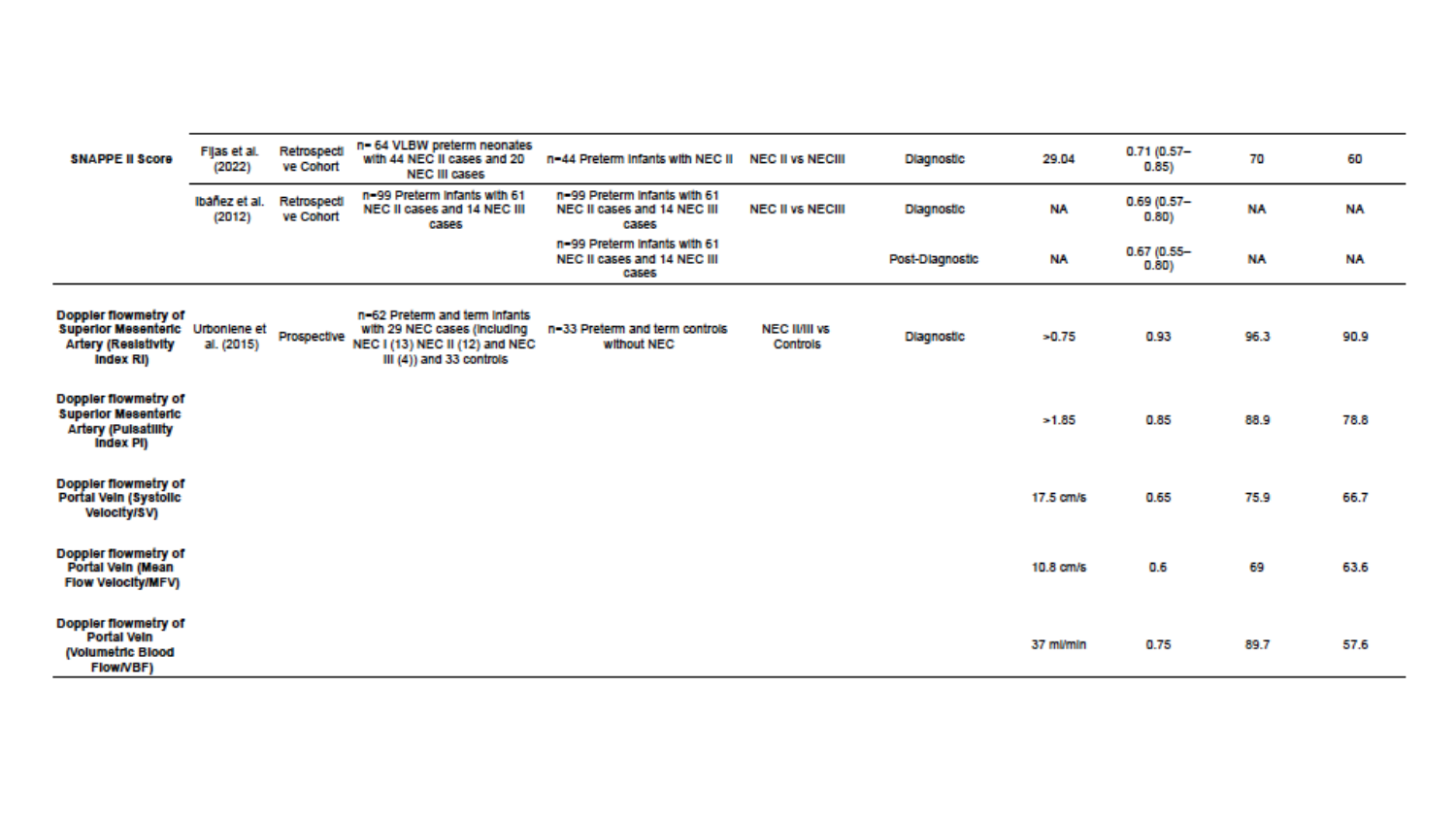

Supplement: Supplementary file 3 [file Presentation1.pptx]
